# A Comparative Study of the Impact of Chemical Debridement Products on the Proteomic Profile of the Salivary Pellicle and Cell Adhesion on OsseoSpeed Titanium Dental Implant Surfaces

**DOI:** 10.1021/acsami.5c14570

**Published:** 2025-09-18

**Authors:** Angela De Lauretis, Qiang Wang, Marco Santacroce, Bernd Thiede, Qianli Ma, Ståle Petter Lyngstadaas, Jan Eirik Ellingsen, Dirk Linke, Håvard Jostein Haugen

**Affiliations:** † Department of Biomaterials, Institute of Clinical Dentistry, Faculty of Dentistry, University of Oslo, 0455 Oslo, Norway; ‡ Corticalis AS, Oslo Science Park, Gaustadalléen 21, 0349 Oslo, Norway; § Department of Biomedical and Dental Sciences and Morphofunctional Imaging, University of Messina, Via Consolare Valeria, 1, 98125 Messina, Italy; ∥ Department of Biosciences, University of Oslo, 0316 Oslo, Norway; ⊥ Department of Prosthetics and Oral Function, Institute of Clinical Dentistry, University of Oslo, 0455 Oslo, Norway

**Keywords:** peri-implantitis, chemical decontamination, salivary pellicle, proteomics, cell adhesion

## Abstract

*Background and objectives:* Dental implants are commonly used for tooth replacement, with excellent success rates over 10 years. However, exposure to oral cavity bacteria makes them susceptible to biofilm accumulation, which can lead to peri-implant diseases. The salivary pellicle, a protein-rich layer formed on implant surfaces, mediates bacterial adhesion and cell attachment. Effective decontamination is essential for managing bacterial infections, yet no consensus exists on the best chemical agent. Understanding how chemical agents affect the pellicle proteomic profile may guide the selection of optimal treatments. *Methods:* We investigate the impact of chemical decontamination agents on the salivary pellicle on OsseoSpeed-like titanium dental implant surfaces using a BCA assay and proteomics, and on cell adhesion using human gingival fibroblasts (HGF) and human bone marrow mesenchymal stem cells (hBMSC). We compare the effects of hydrogen peroxide (H_2_O_2_), Poloxamer 407 (P407), P407 + H_2_O_2_ and sodium hypochlorite + amino acids (NaOCl + AA) using the salivary pellicle and a clean titanium surface as controls. By quantifying and identifying proteins on titanium surfaces after decontamination, this study reveals how chemical agents affect the salivary pellicle. *Results*: All products reduced the total protein on the surfaces, with the P407 + H_2_O_2_ hydrogel demonstrating lower protein variety and superior effectiveness in preventing surface recontamination. Not all proteins were removed during decontamination procedures, and unique proteins were detected in each experimental condition, suggesting protein readsorption depends on surface chemistry. P407 + H_2_O_2_ shows the highest enrichment in integrin- and cadherin-binding proteins upon recontamination. P407 and P407 + H_2_O_2_ promoted HGFs and hBMSCs attachment. Conversely, NaOCl + AA and H_2_O_2_ showed lower HGFs and hBMSCs counts. *Conclusion:* The P407 + H_2_O_2_ hydrogel had the strongest decontamination effect and limited surface recontamination. Each chemical agent produced a distinct proteomic profile. P407 and P407 + H_2_O_2_ promoted initial cell adhesion.

## Graphical abstract

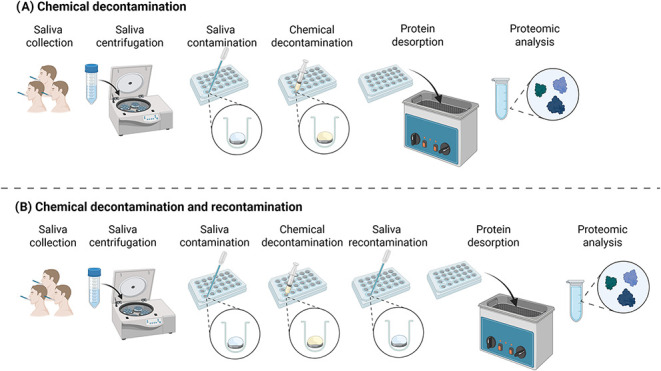

## 1 Introduction

Dental implants have become a widespread treatment modality for replacing missing teeth, with an estimated global market of $13 billion in 2023.^1^ Given the trend of an aging population,^1^ dental implant use is expected to increase significantly.^2^ A major factor behind their success is osseointegration, a biological process where a direct interface forms between the implant and bone, providing enhanced support and preserving the surrounding bone.^3,4^ This has contributed to the excellent performance of dental implants, which achieve an average success rate of 95% over 10 years.^4,5^ However, dental implants are constantly exposed to bacteria in the oral cavity, and biofilm infections on implanted biomaterials remain one of the leading causes of implant failure.^6,7^ Oral microorganisms colonize the implant surface by interacting with the salivary pellicle layer that is deposited on the metal surface, and that constitutes a protein-rich substrate favorable for bacterial adhesion.^8–12^ The ability to modulate this initial protein layer is crucial as it mediates bacterial attachment and, once the bacteria adhere to the surface, they rapidly form a biofilm, which reduces the access of the host immune system and the efficacy of antibiotics.^13^ Furthermore, implant surfaces contaminated by saliva have been shown to negatively affect cell viability, growth and osseointegration.^11,14–16^ While studies have focused on biofilm removal, there is limited understanding of how different decontamination agents alter the protein composition of the salivary pellicle and subsequent cell attachment.

If left untreated, the bacterial biofilms can progress to local infections and result in peri-implant diseases, such as peri-implant mucositis - a reversible inflammatory condition of the soft tissues surrounding an implant - and peri-implantitis, an irreversible inflammatory disease characterized by progressive soft tissue and bone destruction around the implant.^17,18^ These conditions may necessitate interventions, ranging from implant cleaning to complete removal.^19^ The high reported prevalence of peri-implant mucositis (50%) and peri-implantitis (12 to 43%) in dental implant sites underscores the urgent need for effective prevention and treatment strategies.^20^

Treatment methods for periodontal diseases have also been implemented for treating peri-implant diseases due to the similarity between the two conditions.^21^ Unfortunately, these methods have proven less effective for peri-implantitis, which also progresses more severely than periodontitis.^9,17,21^ This can be attributed to differences in the bacterial spectrum between the two conditions, which are influenced by the unique surface properties of dental implants.^9,22^ Characteristics such as surface energy, topography, wettability, and electrochemical charges differ from those of natural teeth:^9^ these factors, along with the salivary proteins and gingival crevicular fluid, influence the composition of the acquired pellicle and subsequently determine the bacterial species that colonize the implant surface^9,23^ and cell attachment.^11^ In addition, collagen fibers of dental implants are parallel to the implant surface, unlike the perpendicular insertion into the cementum seen in natural teeth.^24^ Combined with the reduced vascularity of peri-implant connective tissue, this contributes to the low resilience of the implants to bacterial contamination.^24^ Hence, addressing peri-implant infections by decontaminating the implant surface is essential.^21,25–27^ Despite the importance of decontamination in peri-implant diseases, little is known about how different decontamination methods influence pellicle formation and recontamination.

Implant decontamination is typically achieved through mechanical and chemical debridement methods or antimicrobial agents.^28–31^ Ideally, decontamination should minimize surface damage while avoiding a more conducive environment for bacterial colonization.^32^ Chemical decontamination is widely used for prevention and treatment; however, clinical results for its use as an adjunctive to mechanical debridement vary significantly.^33^ Therefore, there is no consensus on the best approach, highlighting the need for further investigation.^34–38^

This work addresses a critical gap by providing a comparative proteomic analysis of salivary pellicle formation and recontamination and its effect on cell attachment when using different chemical decontamination agents. Specifically, by investigating the impact of hydrogen peroxide (H_2_O_2_), Poloxamer 407 (P407), P407 + H_2_O_2_ and sodium hypochlorite + amino acids (NaOCl + AA), the study aims to identify the most efficient product for reducing pellicle formation and limiting recontamination on OsseoSpeed-like titanium surfaces while promoting cell attachment. P407 was chosen for its thermoreversible properties and micellar structure in hydrogel form,^39^ which offers a detergent effect that facilitates debris entrapment while maintaining a moist environment on the surface. The combination of P407 with H_2_O_2_, a common agent for tooth-whitening and oral hygiene procedures,^40^ represents a novel approach to achieve a synergistic effect between the two components. If one can demonstrate that a decontamination agent reduces protein adsorption, limits surface recontamination and promotes cell attachment, this would help optimize implant maintenance strategies, ultimately contributing to the prevention of peri-implant diseases and enhancing the longevity of dental implants.

## 2 Experimental Section

### 2.1 Materials

Hydrogen peroxide (H_2_O_2_), Poloxamer 407, sodium chloride (NaCl), nitric acid (HNO_3_), ammonium bicarbonate, Triton X-100, Dulbecco’s Phosphate Buffered Saline, Dulbecco’s Modified Eagle’s Medium, d-glucose, paraformaldehyde and mouse monoclonal anti-Vinculin antibody were purchased from Merck Life Sciences AS, Oslo, Norway. Sodium hydroxide (NaOH), hydrofluoric acid (HF), ethanol (EtOH), acetone, urea, dithiothreitol (DTT), tris­(hydroxymethyl)­aminomethane hydrochloride (Tris-HCl), iodoacetamide, formic acid and acetonitrile were purchased from Avantor AS, Oslo, Norway. The radioimmunoprecipitation assay (RIPA) buffer, Gibco Penicillin-Streptomycin (P/S), Gibco Fetal Bovine Serum (FBS; Cat.No A5256701), Dulbecco’s Modified Eagle’s Medium/F-12 Glutamax, Alexa Fluor 568 goat antimouse secondary antibody IgG (H + L), Alexa Fluor 568 Phalloidin, and Hoechst 33342 were purchased from Thermo Fisher Scientific AS, Oslo, Norway. Platelet lysate (PIPL) was sourced from a blood bank in Reykjavik, Iceland. Heparin was purchased from LEO Pharma AS, Ballerup, Denmark. Human gingival fibroblasts (HGFs; Passage 5–7, HFIB-G) were purchased from Provitro, Berlin, Germany. Human bone marrow-derived mesenchymal stem cells (hBMSCs; Passage 6, Cat. No PT-2501) were purchased from Lonza, Basel, Switzerland.

### 2.2 Chemical Decontamination Products

Four decontamination products were used in this study and compared to a clean titanium surface and the salivary pellicle serving as controls (Table 1):

**Table 1:** Decontamination Products Used in the Present Study

**product**	**description**	**abbreviation**
MucoPrep (Corticalis AS, Oslo, Norway)	Poloxamer 407	P407
hydrogen peroxide	3%v/v H_2_O_2_ in water	H_2_O_2_
PeriPrep (Corticalis AS, Oslo, Norway)	Poloxamer 407 + H_2_O_2_	P407 + H_2_O_2_
**Perisolv** **(Regedent AG**, **Zurich**, **Switzerland)**	carboxymethyl cellulose hydrogel containing 0.5% sodium hypochlorite (NaOCl), titanium dioxide and amino acids (glutamic acid, leucine, lysine)	NaOCl + AA

### 2.3 Titanium Disc Surface Treatment

Thirty machined grade 2 titanium discs with a diameter of 6.2 mm and a height of 2 mm were provided by a mechanical workshop at the Faculty of Medicine, University of Oslo (Oslo, Norway). The discs were grit-blasted with TiO_2_ (180–220 μm) and washed with 40%v/v NaOH, 50%v/v HNO_3_ and Milli-Q water in an ultrasonic bath; upon reaching a neutral pH, the discs were acid-etched in 0.2%v/v HF at pH 2.1 for 2 min, according to a previously described procedure.^41^ The resulting surfaces mimic the commercial OsseoSpeed dental implant surface (Dentsply Sirona, Charlotte, NC). The discs were stored at room temperature in EtOH until utilization.

### 2.4 Dental Salivary Pellicle Model and Chemical Decontamination

Saliva was collected during masticatory stimulation with paraffin pellets (GC Europe N.V., Leuven, Belgium) from three voluntary, nonsmoker donors who abstained from eating and drinking for at least 2 h before collection. Saliva was pooled and centrifuged at 12,000 rpm for 4 min at 4 °C (Multifuge X1R, Thermo Fisher Scientific, Waltham). To ensure a comprehensive proteomics analysis, the saliva was neither sterilized through filtration nor pasteurized, as such processes have been shown to reduce the number of identified proteins.^42^

The discs were placed in a well plate, and 20 μL of saliva were added on top of each disc, ensuring coverage was restricted to the surface while preventing protein adsorption on the lateral walls of the discs. This volume was selected to maintain a stable saliva droplet, minimizing the risk of overflow and allowing for the precise evaluation of proteins exclusively present on the OsseoSpeed-like surface, thereby reducing the potential protein overestimation. The well plate was placed in a TS8056 series incubator (Termarks, Fjärås, Sweden) at 37 °C for 2 h to acquire the salivary pellicle, aligning with the parameters used in previous studies.^43–45^

Three discs per chemical decontamination agent were used in two parallel experiments: the first set was decontaminated and analyzed, while the second set was decontaminated, recontaminated with saliva and analyzed (Figure 1). Specifically, chemical decontamination was performed by applying 20 μL of product on the disc surface for 2 min and then rinsing for 12 s with 0.9%w/v saline solution. Recontamination was similar to the original salivary pellicle formation procedure above.

**Figure 1:**
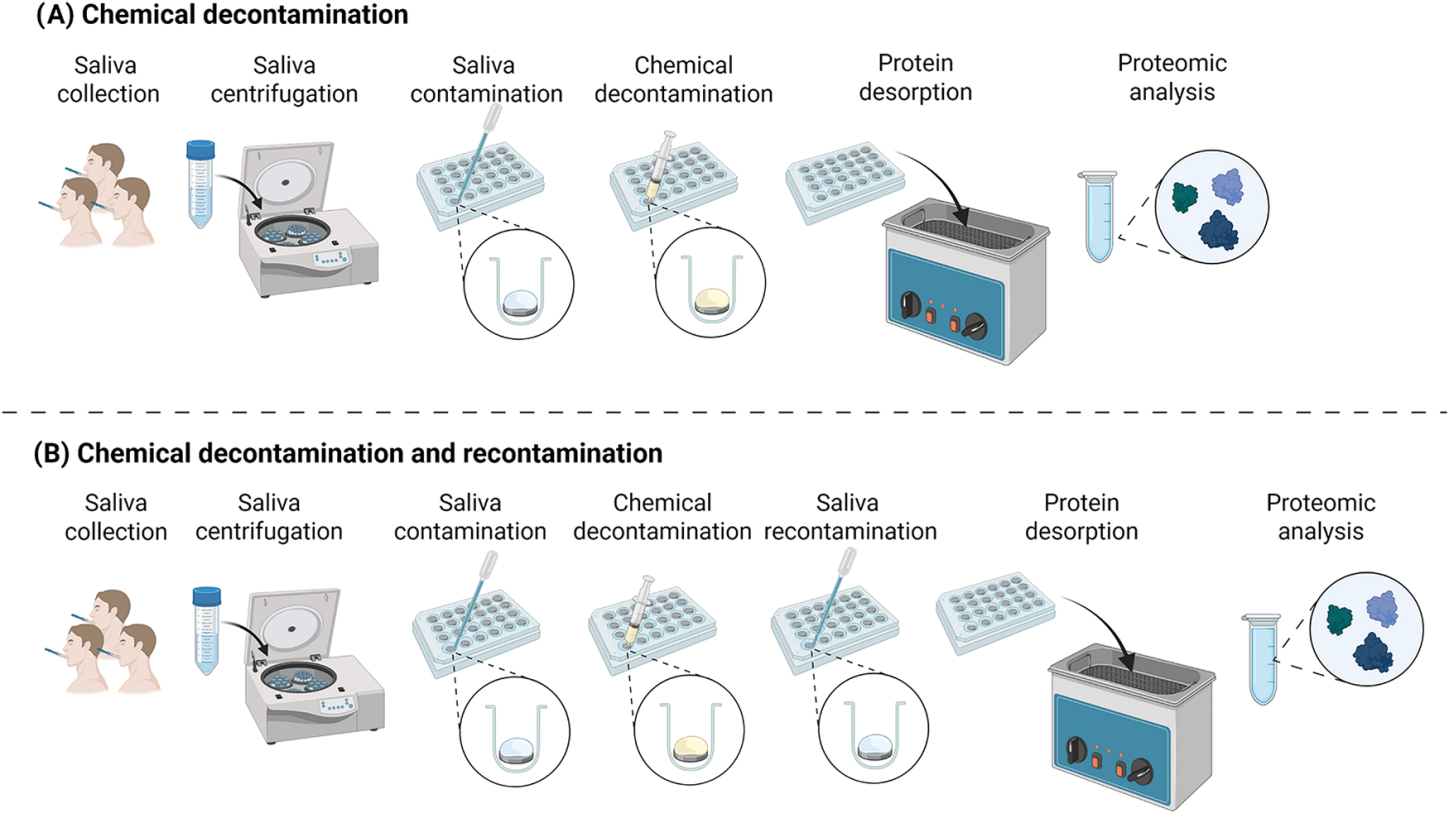
Schematic representation of the experimental setup of the dental pellicle model. (A) Chemical decontamination and proteomic analysis; (B) Chemical decontamination, recontamination with saliva and proteomic analysis.

### 2.5 Protein Desorption and Quantification

The adsorbed salivary proteins on the surface of the titanium discs were desorbed through ultrasonication in a water bath at room temperature for 30 min using 150 μL of RIPA buffer per well.

The concentration of the salivary proteins detached from the disc surfaces was determined using the Pierce MicroBCA Protein Assay Kit (Thermo Fisher Scientific AS, Oslo, Norway), conducting the experiment in triplicates. The protein concentrations were expressed in (μg/mL). Bovine serum albumin (BSA) was serially diluted in Milli-Q water to prepare a set of standards following the manufacturer’s instructions, while the samples of desorbed proteins in RIPA buffer were diluted 1:10 with Milli-Q water. The assay was performed according to the microplate procedure.

### 2.6 In-Solution Digestion

The proteins were precipitated by adding four volumes of ice-cold acetone, vortexing, and incubating at −20 °C overnight. Subsequently, the samples were centrifuged at 16,000 rpm for 20 min at 4 °C (Centrifuge 5415R, Eppendorf, Hamburg, Germany), and the supernatant was discarded. The pellet containing the precipitated proteins was dissolved in 50 μL 6 M urea and 100 mM ammonium bicarbonate, pH 7.8. For the reduction and alkylation of cysteines, 2.5 μL of 200 mM DTT in 100 mM Tris-HCl, pH 8, was added, and the samples were incubated at 37 °C for 30 min followed by the addition of 7.5 μL 200 mM iodoacetamide for 1 h at room temperature in the dark. The alkylation reaction was quenched by adding 10 μL 200 mM DTT at 37 °C for 30 min. Subsequently, 200 μL 50 mM ammonium bicarbonate, pH 7.8, was added, and the proteins were digested with 5 μg trypsin GOLD (Promega, Madison, WI) for 16 h at 37 °C. The digestion was stopped by adding 5 μL 50%v/v formic acid, and the generated peptides were purified using a 10 μL OMIX C18 micro-SPE pipet tip (Agilent, Santa Clara, CA) and dried using a Speed Vac concentrator (Concentrator Plus, Eppendorf, Hamburg, Germany).

### 2.7 Liquid Chromatography–Mass Spectrometry Analysis

The samples were analyzed by liquid chromatography–mass spectrometry (LC-MS) using a timsTOF Pro (Bruker Daltonik, Bremen, Germany), which was coupled online to a nanoElute nanoflow liquid chromatography system (Bruker Daltonik, Bremen, Germany) via a CaptiveSpray nanoelectrospray ion source. The dried peptides were dissolved in 4 μL 0.1%v/v formic acid, and 2 μL of the sample were injected. The peptides were separated on a reversed phase Aurora Elite CSI UHPLC column (C18, 15 cm x 75 μm, 1.7 μm - IonOpticks, Fitztroy, VIC, Australia). Mobile phase A contained water with 0.1%v/v formic acid, and acetonitrile with 0.1%v/v formic acid was used as mobile phase B. The peptides were separated by a gradient from 0 to 35% mobile phase B over 30 min at a flow rate of 200 nL/min at a column temperature of 50 °C. MS acquisition was performed in DDA-PASEF mode. The capillary voltage was set to 1.5 kV with a mass range of 100 to 1700 *m*/*z*. The number of PASEF ranges was set to 20 with a total cycle time of 1.16 s, a charge up to 5, a target intensity of 20000, an intensity threshold of 1750, and active exclusion with release after 0.4 min. An inversed reduced TIMS mobility (1/*k*_0_) of 0.85–1.40 Vs/cm^2^ was used with a range time of 100 ms, an accumulation time of 100 ms, a duty cycle of 100%, and a ramp rate of 9.51 Hz. Precursors for data-dependent acquisition were fragmented with an ion mobility-dependent collision energy, which was linearly increased from 20 to 59 eV.

### 2.8 Database Search

The LC/MS data were searched against the UniProt database of *Homo sapiens* (20429 entries), using FragPipe (v22.0) with MSFragger (v4.1).^46^ The following search parameters were used: a precursor mass tolerance of 15 ppm, a fragment mass tolerance of 0.03 Da, trypsin (strict) as enzyme, a peptide length of 8–50, and a peptide mass range of 500–5000. Methionine oxidation and N-terminal protein acetylation were set as variable modifications, while cysteine carbamidomethylation was a fixed modification.

### 2.9 Label-Free Quantification

FragPipe-Analyst^47^ was used for label-free quantification (LFQ). The following parameters were applied: a MaxLFQ intensity as intensity type, a minimum percentage of nonmissing values in at least one condition of 50, a DE adjusted p-value cutoff of 0.05, a DE Log2 fold change cutoff of 1, a variance stabilizing normalization as normalization type, a Perseus-type as imputation type, and the Benjamini-Hochberg as type of FDR correction.

### 2.10 Gene Ontology and Pathway Enrichment Analysis

The UniProt accession numbers of the identified proteins were used as input into the Database for Annotation, Visualization, and Integrated Discovery (DAVID).^48,49^ Gene Ontology (GO) analysis was conducted to categorize proteins based on their molecular function (MF) terms. Additionally, pathway enrichment analysis was performed using the Kyoto Encyclopedia of Genes and Genomes (KEGG) and REACTOME databases to identify biological pathways significantly associated with the pellicles.

### 2.11 Cell culture, Immunofluorescent Staining and Confocal Microscopy

To investigate cell adhesion, human gingival fibroblasts (HGFs) and human bone marrow mesenchymal stem cells (hBMSCs) were analyzed using immunofluorescence staining, followed by confocal microscopy.

HGFs were cultured in a low-glucose Dulbecco’s Modified Eagle’s Medium supplemented with 10% FBS, 1% P/S and 5 mM d-glucose. hBMSCs were cultured in a Dulbecco’s Modified Eagle’s Medium/F12 with Glutamax supplemented with 10% platelet lysate, 1% P/S, and 2 IU/mL heparin.

A total of 1.02 × 10^6^ cells were resuspended in 18 mL of medium and transferred to cell culture wells, using 0.3 mL per well. Cells were seeded onto the surface of titanium discs, using three discs per experimental condition, and cultured at 37 °C with 5% CO_2_ for 30, 60, and 120 min, respectively. After incubation, the medium was gently removed, and the cells were rinsed once with PBS and fixed in 4% (w/v) paraformaldehyde (PFA) for 15 min at room temperature. PFA was then removed, and the cells were washed in cold PBS for 10 min.

Cells were permeabilized with 0.1% Triton X-100 in PBS for 5 min at room temperature. The permeabilization solution was removed, and the cells were rinsed again with PBS. To block nonspecific antibody binding, cells were incubated with 1% bovine serum albumin (BSA) in PBS for 1 h at room temperature.

Primary staining was performed by incubating the cells with mouse monoclonal anti-Vinculin antibody diluted 1:400 in 1% BSA/PBS overnight at 4 °C, using 150 μL per well. Following incubation, cells were washed three times with PBS for 5 min each.

The samples were stained with goat antimouse secondary antibody IgG (H + L), Alexa Fluor 568 diluted 1:500 in 1% BSA/PBS for 1 h at room temperature in the dark. The cells were washed once with PBS for 5 min and then incubated with Alexa Fluor 488 Phalloidin (1:400) for 1 h at room temperature. Afterward, cells were washed twice with PBS for 5 min each.

Cells were counterstained with Hoechst 33342 (10 mg/L in PBS) for 15 min at room temperature, followed by one final PBS rinse.

The samples were imaged using a laser scanning confocal microscope (SP8, Leica, Wetzlar, Germany). *Z*-stack images were acquired, and three-dimensional reconstructions were generated using the Fiji software (NIH). The cell number and the vinculin (yellow channel) integrated density (IntDen) were measured using ImageJ (NIH). Dead cells, identified by the lack of cytoskeleton, were manually counted and subtracted from the computed cell number to obtain the viable cell number.

### 2.12 Statistic Analysis

A Kruskal–Wallis rank-sum test followed by a Dunn’s multiple comparison test (ranked test) was performed with GraphPad Prism version 10.4 (GraphPad Software Inc., San Diego, CA) to identify any statistically significant differences between the experimental conditions.

## 3 Results

### 3.1 Protein Quantification

The amount of adsorbed proteins per area of the titanium surface following chemical decontamination and subsequent recontamination with saliva was measured using the Pierce MicroBCA Protein Assay Kit. Quantification was performed by subtracting the blank values obtained from Milli-Q water in the MicroBCA Assay and converting the adsorbed protein concentration in (μg/mL) to adsorbed protein amount per area (μg/mm^2^) using the total volume of the samples and the area of the titanium disc. The results shown in Figure 2 indicate that a clean titanium surface shows a significantly lower protein amount per area compared to a titanium disc coated with salivary pellicle (*p* = 0.0076). Particularly, decontamination with H_2_O_2_ (*p* = 0.0201), P407 (*p* = 0.0007) and P407 + H_2_O_2_ (*p* = 0.0006) significantly lowered the amount of adsorbed proteins per area compared to the salivary pellicle, while the reduction was not significant when using NaOCl + AA. After recontamination, none of the chemical agents significantly reduced the amount of adsorbed proteins per area compared to the salivary pellicle. However, H_2_O_2_ (*p* = 0.0136), P407 (*p* = 0.0101), and NaOCl + AA (*p* = 0.0003) showed a significant increase in the amount of adsorbed proteins per area compared to the clean titanium surface, while this was not observed when using the P407 + H_2_O_2_ hydrogel.

**Figure 2:**
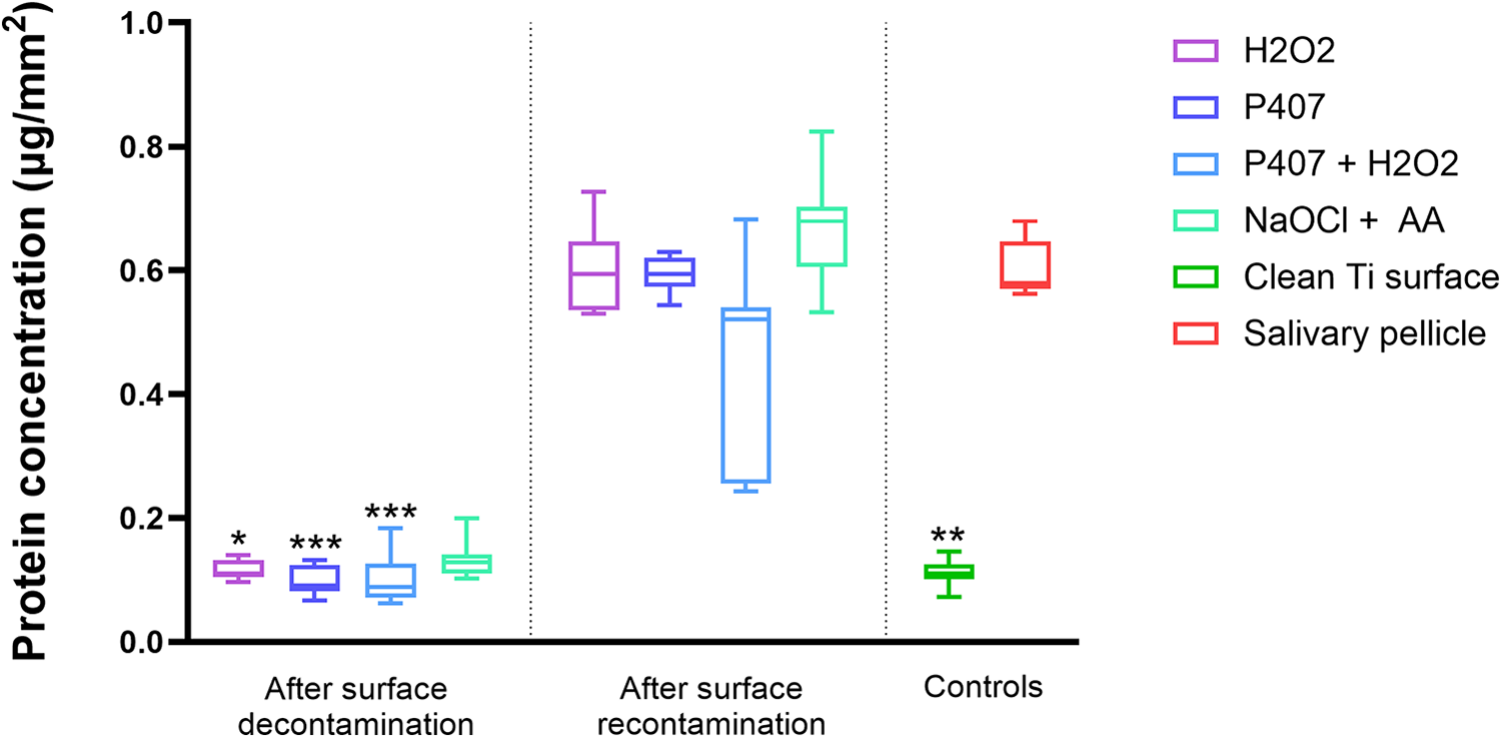
Protein amount per area of the titanium disc (μg/mm^2^) following chemical decontamination and subsequent recontamination using H_2_O_2_, P407, P407 + H_2_O_2_, and NaOCl + AA, compared to the salivary pellicle acquired on the OsseoSpeed-like titanium surface and to a clean titanium surface. The asterisks represent a statistically significant difference compared to the salivary pellicle according to a Kruskal–Wallis rank-sum test followed by a Dunn’s multiple comparison test (*< 0.5, **< 0.01, *** *p* < 0.001).

### 3.2 Proteomics Analysis

We identified the diversity of proteins in pooled saliva, the salivary pellicle, and the experimental conditions following decontamination and subsequent recontamination, including only proteins consistently identified in three independent replicates. Keratins, which are common contaminants from sample handling, were excluded from the analysis. Proteins found in the positive control, i.e., clean titanium surface treated with the RIPA buffer, have been subtracted from the data sets. A total of 926 different proteins were identified in pooled saliva, while 410 proteins were adsorbed onto the OsseoSpeed-like surface, forming the salivary pellicle (Figure 3A). Not all proteins found in pooled saliva were present in the salivary pellicle. The two groups shared 363 proteins, and 47 proteins were uniquely detected in the salivary pellicle. Presumably, these are proteins that are present in the saliva in very low concentrations but that are enriched in the pellicle.

**Figure 3:**
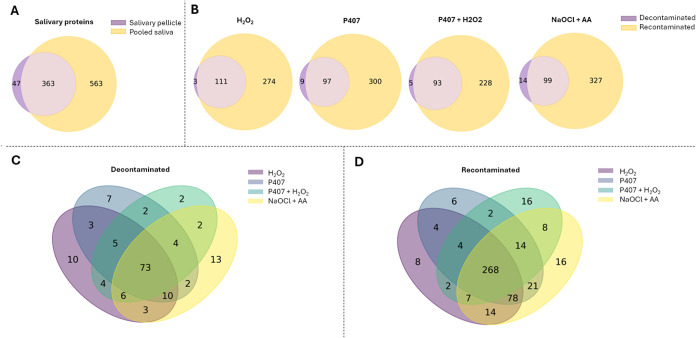
Venn diagrams displaying the number of identified proteins: (A) Comparison between the salivary pellicle and pooled saliva; (B) Comparison after decontamination and after recontamination with the same chemical agent; (C) Comparison after decontamination among the experimental conditions; (D) Comparison after recontamination among the experimental conditions.

All experimental conditions exhibited a higher diversity of proteins after recontamination compared to after decontamination (Figure 3B). While some proteins were uniquely detected either postdecontamination or postrecontamination, only a few were exclusive to the decontaminated state. Additionally, decontamination was not entirely successful in removing all proteins from the surface. When comparing the proteins identified after decontamination across the experimental conditions, most proteins (73) were commonly identified on the surfaces (Figure 3C). Still, some proteins were also uniquely present in each test group, particularly in the NaOCl + AA treated surface. Figure 3D compares protein adsorption among the experimental conditions following recontamination with saliva. In this case, salivary proteins tend to readsorb, with 268 proteins commonly identified across experimental conditions. However, a few unique proteins were identified in each condition, particularly with P407 + H_2_O_2_ and NaOCl + AA.

Tables S1 to S11 (Supporting Information) list the proteins identified in pooled saliva, the salivary pellicle, the positive control (i.e., RIPA buffer) and the experimental conditions after decontamination and after recontamination, respectively. As it is typical in saliva proteins,^10,11^ mucins, amylase, serum albumin, cystatins, and immunoglobulins were consistently identified in all samples.

The Principal Component Analysis (PCA) plot (Figure 4) shows that after decontamination, the experimental conditions exhibit a broader clustering at high PC1 and low-to-medium PC2, indicating more variability in the proteomic profile of the remaining proteins adsorbed onto the surface. Upon recontamination, all the experimental conditions tightly cluster at low PC1 and PC2, aligning with the salivary pellicle, thus suggesting a partial restoration of the initial pellicle proteomic profile. The pooled saliva samples cluster closely at low PC1 and high PC2, demonstrating a diverse protein composition than the other experimental conditions.

**Figure 4:**
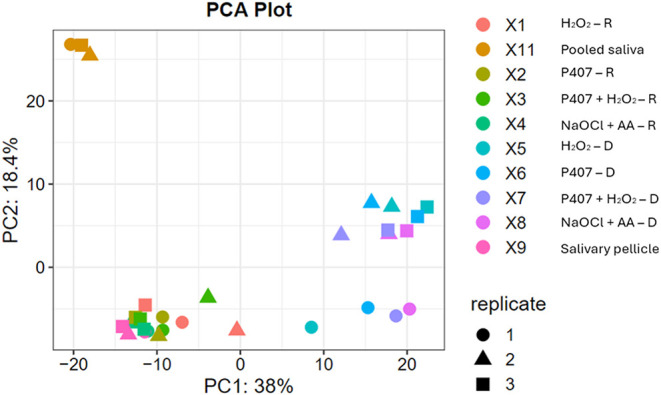
PCA plot illustrating the clustering patterns of protein profiles across the different experimental conditions, compared to the salivary pellicle acquired on the OsseoSpeed-like titanium surface and the whole saliva sample – D: after chemical decontamination; R: after saliva recontamination.

Figure 5 presents an overview of the significant differentially adsorbed proteins among the samples (n = 3/group). Red shades indicate high protein abundance, while blue shades indicate low protein abundance. Similar trends were observed between the salivary pellicle and the experimental conditions after recontamination. In contrast, experimental conditions following chemical decontamination exhibit a different proteomic profile.

**Figure 5:**
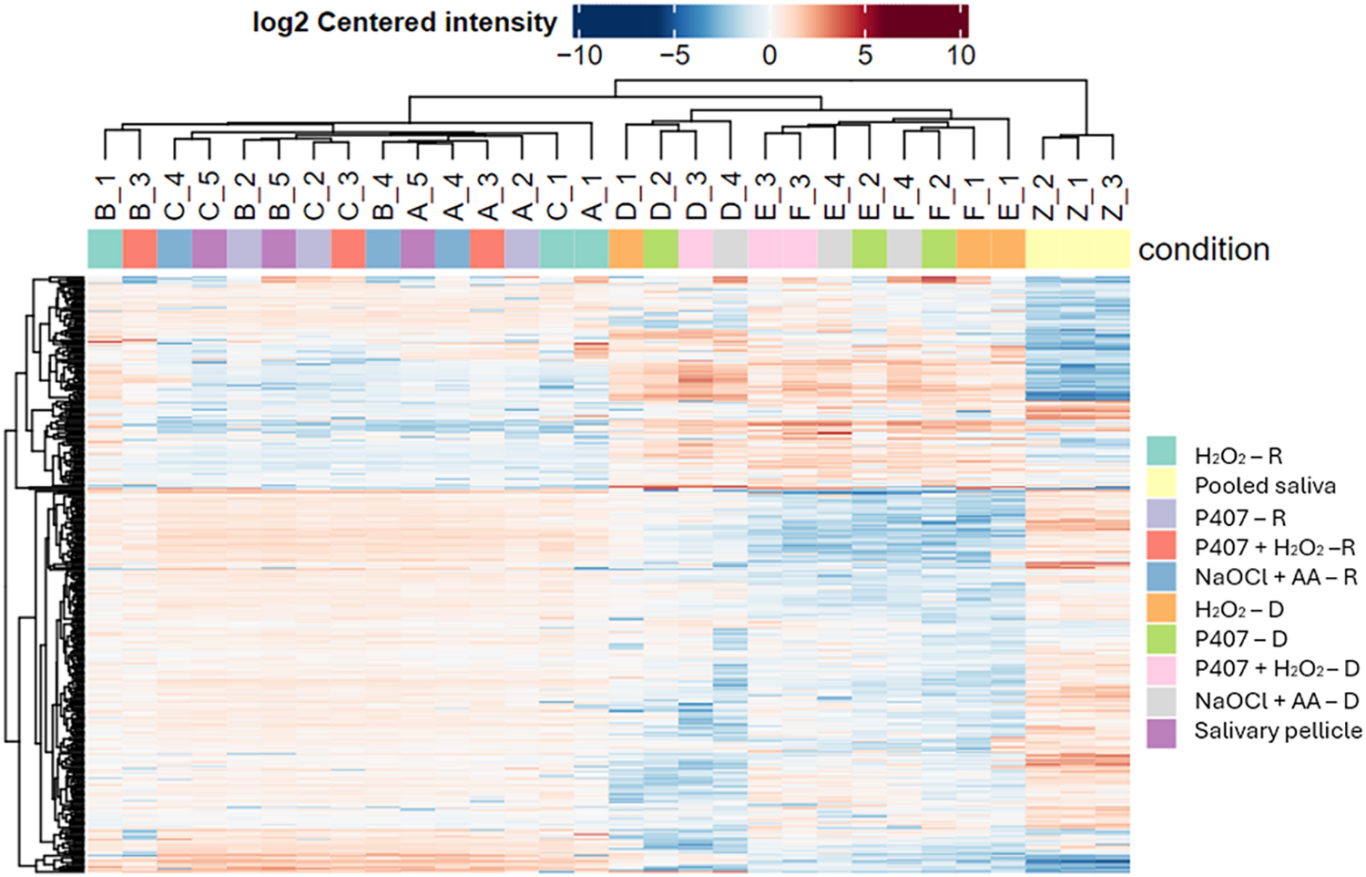
Heat map showing an overview of all significantly differentially adsorbed proteins among experimental conditions –D: after chemical decontamination; R: after saliva recontamination. *n* = 3.

Volcano plots were used to identify proteins differentially adsorbed after chemical decontamination and subsequent recontamination with saliva for each chemical agent (Figure 6). The analysis revealed clear shifts in protein profiles between postdecontamination and postrecontamination states. Recontaminated surfaces consistently showed an increased number of significantly differentially adsorbed proteins compared to their decontaminated states, although this effect was less marked in the NaOCl + AA treatment.

**Figure 6:**
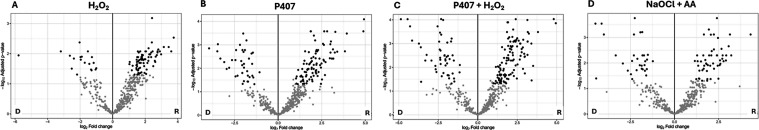
Volcano plots showing differential protein abundance between decontaminated (D) and recontaminated (R) experimental conditions. Proteins with significant changes in abundance are highlighted in black. (A) H_2_O_2_; (B) P407; (C) P407 + H_2_O_2_; (D) NaOCl + AA.

Table 2 presents a list of selected proteins known to be involved in the biointegration of dental implants, as identified by Zuanazzi et al,^50^ and those affecting bacterial adhesion, as established from previous research,^51–55^ specifying their presence in the different experimental conditions analyzed in this study. Several proteins exhibit multiple biological functions. For instance, apolipoprotein A-I and α-1-antitrypsin are key players in both tissue regeneration and immune response. Similarly, desmoglein-3 contributes to biological adhesion and tissue regeneration. Following decontamination, each agent exhibited a distinct reduction in protein diversity compared to the salivary pellicle. The NaOCl + AA hydrogel removed the most proteins, including apolipoprotein A-I, cystatin-B, cystatin-C, myeloperoxidase, desmoglein-3, transketolase, α-1-antitrypsin, small proline-rich protein 3, peptidyl-prolyl cis–trans isomerase, and leukocyte elastase inhibitor. The H_2_O_2_ solution and the P407 and P407 + H_2_O_2_ hydrogels also removed a similar range of proteins but maintained cystatin-B, cystatin-C and leukocyte elastase inhibitor. Additionally, P407 treatment allowed for the retention of α-1-antitrypsin. Concerning the identified tissue regeneration, immune response, biological adhesion and biomineralization proteins, the NaOCl + AA hydrogel removed the highest number of proteins, while Poloxamer 407 had the mildest impact.

**Table 2:** List of Selected Proteins Involved in the Biointegration of Dental Implants and in Mediating Bacterial Adhesion, and Their Presence in Experimental Conditions Analyzed in This Study

involvement	accession number	protein name	pooled saliva	salivary pellicle	H2O2 D	H2O2 R	P407 D	P407 R	P407 + H2O2 D	P407 + H2O2 R	NaOCl + AA D	NaOCl + AA R
tissue regeneration	P02647	apolipoprotein A-I	yes	yes	no	yes	no	yes	no	yes	no	yes
	P68871	hemoglobin subunit β	yes	yes	yes	yes	yes	yes	yes	yes	yes	yes
	P02788	lactotransferrin	yes	yes	yes	yes	yes	yes	yes	yes	yes	yes
	P05109	protein S100-A8	yes	yes	yes	yes	yes	yes	yes	yes	yes	yes
	P06702	protein S100-A9	yes	yes	yes	yes	yes	yes	yes	yes	yes	yes
	P25311	zinc-α-2-glycoprotein	yes	yes	yes	yes	yes	yes	yes	yes	yes	yes
	P01034	cystatin-C	yes	yes	yes	yes	yes	yes	yes	yes	no	yes
	P05164	myeloperoxidase	yes	yes	no	yes	no	yes	no	yes	no	yes
	P01833	polymeric immunoglobulin receptor	yes	yes	yes	yes	yes	yes	yes	yes	yes	yes
	P32926	desmoglein-3	yes	yes	no	yes	no	yes	no	yes	no	yes
	P29401	transketolase	yes	yes	no	yes	no	yes	no	yes	no	yes
	P01009	α-1-antitrypsin	yes	yes	no	no	yes	no	no	no	no	no
	Q9UBC9	small proline-rich protein 3	yes	yes	no	yes	no	yes	no	yes	no	yes
	P23284	peptidyl-prolyl cis–trans isomerase	yes	yes	no	yes	no	yes	no	no	no	yes
	P02647	apolipoprotein A-I	yes	yes	no	yes	no	yes	no	yes	no	yes
	Q96DR5	BPI fold-containing family A member 2	yes	yes	yes	yes	yes	yes	yes	yes	yes	yes
	Q8TDL5	BPI fold-containing family B member 1	yes	yes	yes	yes	yes	yes	yes	yes	yes	yes
	P04080	cystatin-B	yes	yes	yes	yes	yes	yes	yes	yes	no	yes
	P04406	glyceraldehyde-3-phosphate dehydrogenase	yes	yes	yes	yes	yes	yes	yes	yes	yes	yes
	P68871	hemoglobin subunit β	yes	yes	yes	yes	yes	yes	yes	yes	yes	yes
	P22079	lactoperoxidase	yes	yes	yes	yes	yes	yes	yes	yes	yes	yes
	P61626	lysozyme C	yes	yes	yes	yes	yes	yes	yes	yes	yes	yes
	P05109	protein S100-A8	yes	yes	yes	yes	yes	yes	yes	yes	yes	yes
immune response	P06702	protein S100-A9	yes	yes	yes	yes	yes	yes	yes	yes	yes	yes
	P02788	lactotransferrin	yes	yes	yes	yes	yes	yes	yes	yes	yes	yes
	Q8N4F0	BPI fold-containing family B member 2	yes	yes	yes	yes	yes	yes	yes	yes	yes	yes
	P01034	cystatin-C	yes	yes	yes	yes	yes	yes	yes	yes	no	yes
	P05164	myeloperoxidase	yes	yes	no	yes	no	yes	no	yes	no	yes
	P01833	polymeric immunoglobulin receptor	yes	yes	yes	yes	yes	yes	yes	yes	yes	yes
	P01876	Ig α-1 chain C region	yes	yes	yes	yes	yes	yes	yes	yes	yes	yes
	P01009	α-1-antitrypsin	yes	yes	no	no	yes	no	no	no	no	no
	P30740	leukocyte elastase inhibitor	yes	yes	yes	yes	yes	yes	yes	yes	no	yes
	P02647	apolipoprotein A-I	yes	yes	no	yes	no	yes	no	yes	no	yes
	P06702	protein S100-A9	yes	yes	yes	yes	yes	yes	yes	yes	yes	yes
cell adhesion	P25311	zinc-α-2-glycoprotein	yes	yes	yes	yes	yes	yes	yes	yes	yes	yes
	P60709	actin, cytoplasmic 1	yes	yes	yes	yes	yes	yes	yes	yes	yes	yes
	P32926	desmoglein-3	yes	yes	no	yes	no	yes	no	yes	no	yes
	P02788	lactotransferrin	yes	yes	yes	yes	yes	yes	yes	yes	yes	yes
	P04080	cystatin-B	yes	yes	yes	yes	yes	yes	yes	yes	no	yes
	P05109	protein S100-A8	yes	yes	yes	yes	yes	yes	yes	yes	yes	yes
biomineralization	P06702	protein S100-A9	yes	yes	yes	yes	yes	yes	yes	yes	yes	yes
	P01034	cystatin-C	yes	yes	yes	yes	yes	yes	yes	yes	no	yes
	P23284	peptidyl-prolyl cis–trans isomerase B	yes	yes	no	yes	no	yes	no	no	no	yes
bacterial adhesion	P01876	immunoglobulin heavy constant α 1	yes	yes	yes	yes	yes	yes	yes	yes	yes	yes
	P01877	immunoglobulin heavy constant α 2	yes	yes	yes	yes	yes	yes	yes	yes	yes	yes
	P01857	immunoglobulin heavy constant γ 1	yes	yes	yes	yes	yes	yes	yes	yes	yes	yes
	P01859	immunoglobulin heavy constant γ 2	yes	yes	yes	yes	yes	yes	yes	yes	yes	yes
	P01860	immunoglobulin heavy constant γ 3	yes	yes	no	no	no	no	yes	yes	yes	yes
	P01861	immunoglobulin heavy constant γ 4	yes	yes	no	no	no	no	yes	yes	yes	yes
	P0DTE7	α-amylase 1B	yes	yes	yes	yes	yes	yes	yes	yes	yes	yes
	P19961	α-amylase 2B	yes	yes	yes	no	no	no	yes	yes	yes	yes
	Q8TAX7	mucin-7	yes	no	no	no	no	no	no	no	no	no
	P01036	cystatin-S	yes	yes	yes	yes	yes	yes	yes	yes	yes	yes
	P09228	cystatin-SA	yes	yes	yes	yes	yes	yes	yes	yes	yes	yes
	P28325	cystatin-D	yes	yes	yes	yes	yes	yes	yes	yes	yes	yes

After recontamination with saliva, treatment with chemical agents did not significantly hinder protein readsorption to the new salivary pellicle, except for α-1-antitrypsin. Notably, this was initially retained after decontamination with the P407 hydrogel but was absent after recontamination, suggesting a dynamic adsorption–desorption process. Moreover, decontamination with P407 + H_2_O_2_ hindered the readsorption of peptidyl-prolyl cis–trans isomerase. Overall, the P407 + H_2_O_2_ hydrogel had the highest impact, inhibiting the readsorption of both α-1-antitrypsin and peptidyl-prolyl cis–trans isomerase, whereas the other chemical agents only limited α-1-antitrypsin readsorption.

Proteins like immunoglobulins A and G, mucin-7, amylase, and cystatins have been associated with bacterial adhesion.^51–55^ Immunoglobulins A and G were consistently present across all groups, including experimental conditions after decontamination and subsequent recontamination. However, immunoglobulins heavy constant γ 3 and 4 were removed by all chemical decontamination agents but readsorbed following recontamination with saliva. In the case of amylase, α-amylase 1B was detected in all conditions, while α-amylase 2B was removed by all chemical agents except H_2_O_2_, although it reappeared in all experimental conditions after recontamination. Interestingly, mucin-7 was exclusively detected in pooled saliva.

Gene ontology (GO) and pathway enrichment (KEGG and REACTOME) analyses were conducted in DAVID to investigate the molecular functions and biological pathways associated with the early stages of cell adhesion. Four selected terms for gene ontology molecular functions and pathways concerning cell adhesion were analyzed. Key terms for cell adhesion included integrin binding (GO:0005178), cadherin binding (GO:0045296), focal adhesion (hsa04510) and extracellular matrix organization (R-HSA-1474244). The results for the experimental conditions following recontamination with saliva are reported in Figure 7.

**Figure 7:**
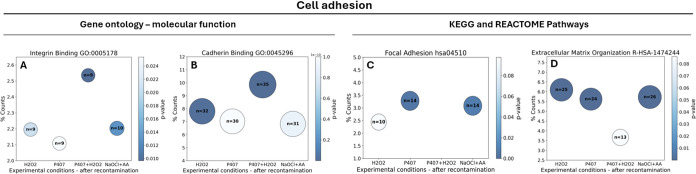
Percentage counts (%), total number of counts (bubble size) and p-value of selected gene ontology - molecular function, KEGG and REACTOME pathway terms related to cell adhesion – (A) Integrin Binding GO:0005178; (B) Cadherin binding GO:0045296; (C) Focal adhesion hsa04510; (D) Extracellular matrix organization R-HSA-1474244.

All experimental conditions exhibited the presence of integrin- and cadherin-binding proteins, with the P407 + H_2_O_2_ hydrogel-treated surface demonstrating the highest level of enrichment. Focal adhesion-related proteins were detected in all groups except for the P407 + H_2_O_2_ condition. Proteins associated with extracellular matrix (ECM) organization were present across all conditions; however, their abundance and enrichment were low with the P407 + H_2_O_2_ treatment.

### 3.3 Viable Cells Count and Vinculin Intensity

The number of viable adherent cells and the normalized vinculin intensity per cell were quantified for HGFs (Figure 8) and hBMSCs (Figure 9) after 30, 60, and 120 min to assess initial cellular responses under the experimental conditions following recontamination with saliva. These were compared to a clean titanium surface and a titanium surface coated with a salivary pellicle.

**Figure 8:**
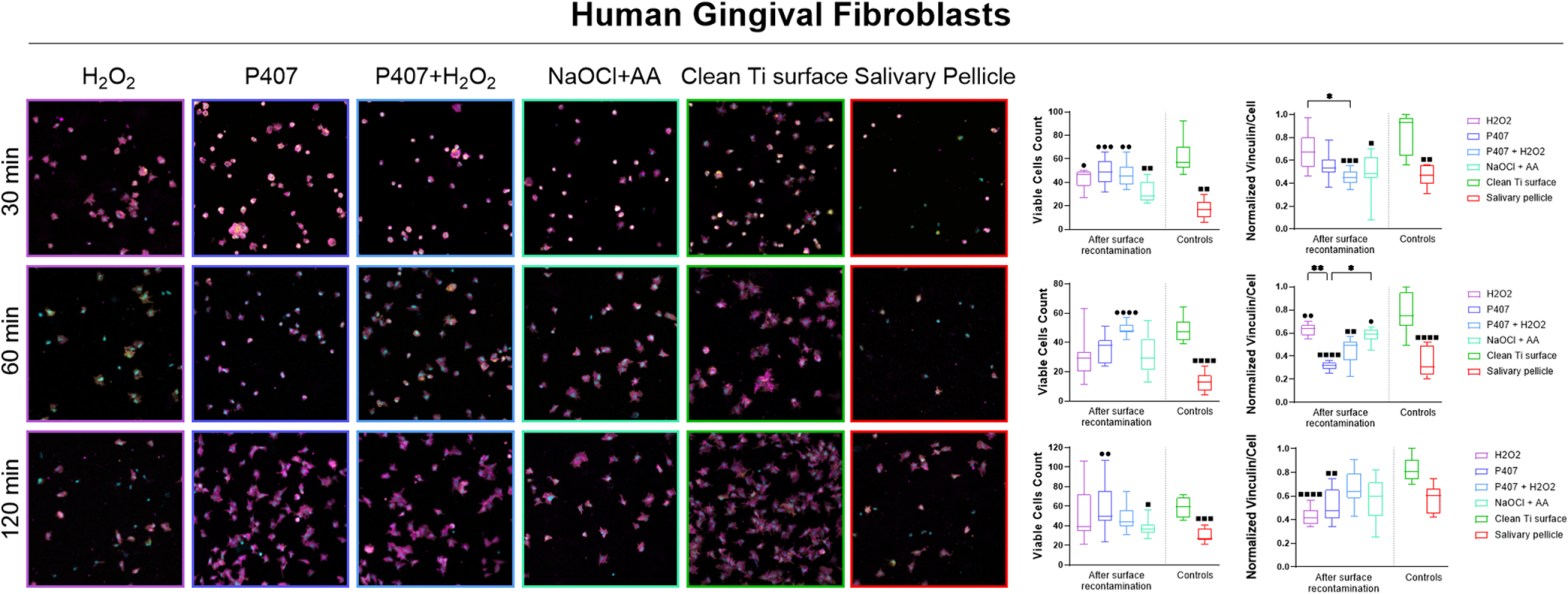
Confocal images at ×20 magnification, viable cells count, and normalized vinculin intensity per cell of Human Gingival Fibroblasts on the OsseoSpeed-like titanium surface following chemical decontamination using H_2_O_2_, P407, P407+ H_2_O_2_, and NaOCl + AA and recontamination with saliva, compared to a clean titanium surface and the acquired salivary pellicle at 30, 60, and 120 min. *, ■ and ● represent a statistically significant difference among the groups, compared to the clean titanium surface and the salivary pellicle, respectively, according to a Kruskal–Wallis rank-sum test followed by a Dunn’s multiple comparison test (*<0.5, **< 0.01, *** *p* < 0.001, **** *p* < 0.0001).

**Figure 9:**
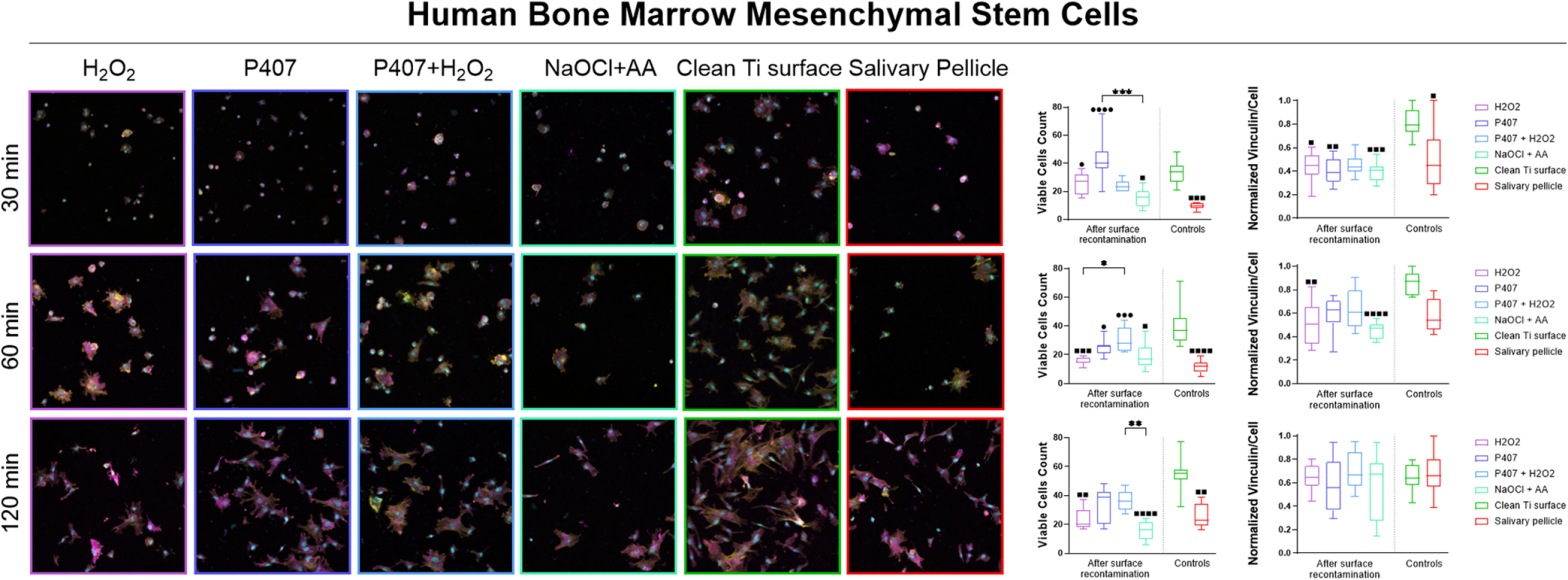
Confocal images at ×20 magnification, viable cells count, and normalized vinculin intensity per cell of human Bone Marrow-Derived Mesenchymal Stem Cells on the OsseoSpeed-like titanium surface following chemical decontamination using H2O2, P407, P407+ H2O2, and NaOCl + AA and recontamination with saliva, compared to a clean titanium surface and the acquired salivary pellicle at 30, 60, and 120 min. *, ■ and ● represent a statistically significant difference among the groups, compared to the clean titanium surface and the salivary pellicle, respectively, according to a Kruskal–Wallis rank-sum test followed by a Dunn’s multiple comparison test (*<0.5, **< 0.01, *** *p* < 0.001, **** *p* < 0.0001).

For HGFs, the number of viable cells was significantly higher on the clean titanium surface than on the salivary pellicle over time, and vinculin intensity was greater up to 60 min. At the 30 min time point, the NaOCl + AA treatment was the only condition to show a significantly lower cell number compared to the clean titanium surface (*p* = 0.0018), while H_2_O_2_ (*p* = 0.0408), P407 (*p* = 0.0009), and P407 + H_2_O_2_ (*p* = 0.0078) resulted in significantly higher HGF numbers compared to the salivary pellicle-coated surface. After 60 min, only P407 + H_2_O_2_ (*p* < 0.0001) demonstrated a significantly increased HGF number compared to the salivary pellicle. At 120 min, P407 (*p* = 0.0070) was the only experimental condition associated with a significantly higher cell count compared to the salivary pellicle. Similar to the 30 min time point, NaOCl + AA (*p* = 0.0426) remained the only treatment yielding a significantly reduced HGF number compared to the clean titanium surface.

Regarding normalized vinculin intensity per cell, at 30 min, intensities were lower for both P407 + H_2_O_2_ (*p* = 0.0004) and NaOCl + AA (*p* = 0.0320) than the clean titanium surface. At 60 min, normalized vinculin intensity was significantly reduced for P407 (*p* < 0.0001) and P407 + H_2_O_2_ (*p* = 0.0068) compared to the clean titanium, whereas H_2_O_2_ (*p* = 0.0025) and NaOCl + AA (*p* = 0.0452) showed higher intensities than the salivary pellicle. By 120 min, H_2_O_2_ (*p* < 0.0001) and P407 (*p* = 0.0051) maintained significantly lower vinculin signals compared to the clean titanium, with no significant differences observed for P407 + H_2_O_2_ and NaOCl + AA.

For hBMSCs, the clean titanium surface consistently displayed a higher number of viable cells over time compared to the salivary pellicle, and vinculin intensity was higher at 30 min (*p* = 0.0320), which was balanced out by 120 min at the 30 min time point, the NaOCl + AA treatment was the only chemical agent that resulted in a significantly lower cell number compared to the clean titanium surface (*p* = 0.0120). In contrast, both H_2_O_2_ (*p* = 0.0436) and P407 (*p* < 0.0001) led to significantly higher cell numbers compared to the salivary pellicle-coated surface. The combination P407 + H_2_O_2_ did not produce any statistically significant difference. At 60 min, only P407 (*p* = 0.0157) and P407 + H_2_O_2_ (*p* = 0.0006) showed a significantly increased number of hBMSCs compared to the salivary pellicle-coated surface. Conversely, H_2_O_2_ (*p* = 0.0007) and NaOCl + AA (*p* = 0.0108) continued to result in significantly lower cell numbers compared to the clean titanium surface. At 120 min, this trend persisted, with both H_2_O_2_ (*p* = 0.0021) and NaOCl + AA (*p* < 0.0001) showing significantly reduced hBMSC numbers relative to the clean titanium surface. Notably, P407 + H_2_O_2_ yielded a significantly higher hBMSC number than NaOCl + AA (*p* = 0.0087). As far as normalized vinculin intensity per cell, at 30 min, the intensities were significantly lower after all treatments, except P407 + H_2_O_2_, compared to the clean titanium surface. At 60 min, a significant reduction in vinculin intensity was observed only for H_2_O_2_ (*p* = 0.0025) and NaOCl + AA (*p* < 0.0001) compared to the clean titanium surface. By 120 min, no statistically significant differences were observed among the groups.

## 4 Discussion

In this study, we aimed to assess the impact of various chemical decontamination products (Table 1) on the acquired salivary pellicle on OsseoSpeed-like dental implant surfaces using a dental pellicle model (Figure 1), focusing on the effectiveness of these products in protein removal and their impact on protein composition. By quantifying the total amount of adsorbed proteins per area of the titanium surface and identifying the specific proteins affected by different decontamination treatments, this study provides insights into how chemical agents interact with the salivary pellicle. We also demonstrate that the decontamination method has a direct impact on the composition of a recontaminating pellicle, with possible consequences for bacterial adhesion and, possibly, subsequent reinfections. Therefore, selecting a decontamination strategy requires careful consideration of its impact on both the pellicle and implant surface properties, which can aid in optimizing peri-implant disease prevention by providing a surface that supports favorable cell responses and resists contamination. Pooled saliva (Table S1) primarily contains proteins such as mucins, amylase, serum albumin, cystatins and immunoglobulins in high concentrations, which were also commonly detected in the salivary pellicle (Table S2) and in the experimental conditions both after decontamination and after recontamination with saliva (Tables S4 to S11). *In vitro* studies by Dorkhan et al. demonstrated that a salivary pellicle containing secretory immunoglobulins A, α-amylase, and cystatin as the main proteins on dental implant surfaces promotes increased metabolic activity and enhanced adhesion of the early colonizer *S. oralis*.^51,52^ Furthermore, proteins such as mucin-7, proline-rich glycoproteins, and amylase have been reported to mediate the adhesion of oral streptococci,^53^ while *S. aureus* specifically binds to mucin-7and immunoglobulins A and G.^54,55^ Comparatively, the adsorption of lactotransferrin on implant surfaces may prevent infection as it inhibits the growth of *P. gingivalis* and *P. intermedia*.^56,57^ Other proteins with antimicrobial properties include lactoperoxidase, lysozyme and antimicrobial peptides.^58^ In this study, serum albumin, mucins, immunoglobulins, amylase, lactotransferrin and lactoperoxidase have been consistently identified in all experimental conditions.

Specifically, a total of 926 proteins were identified in the pooled saliva, whereas 410 were adsorbed onto the OsseoSpeed-like surface (Figure 3A), meaning not all salivary proteins are present in the pellicle. In addition to the complex biological interactions that guide protein adsorption (e.g., protein charge and structure, affinity for the surface, specific protein–protein interactions between the pellicle proteins), another possible explanation for this is the limited time for pellicle acquisition, which was set at 2 h. Remarkably, 47 proteins were uniquely detected on the salivary pellicle and absent in pooled saliva. A systematic review comparing the proteomes of saliva and the salivary pellicle found similar results, identifying proteins uniquely present in the pellicle in a preliminary data set.^59^ However, by setting a stringent criterion of requiring at least three independent experimental identifications for inclusion, the final data set identified the pellicle proteome strictly as a subset of the saliva one.^59^ Proteins with a high affinity for the titanium surface may preferentially accumulate, reaching a detectable concentration only in the salivary pellicle. Additionally, surface interactions may induce conformational changes and promote protein–protein interactions, causing proteins to unfold or expose previously masked domains, thereby altering their detectability. Furthermore, enzymatic cleavage may occur at the surface, likely mediated by proteases from saliva or microbial activity, and might generate peptides uniquely present in the pellicle. Hence, these factors might explain the discrepancy between the pellicle and the pooled saliva proteome in this study.

The protein quantification (Figure 2) revealed that all chemical agents but NaOCl + AA significantly reduced the total amount of surface-adsorbed proteins compared to the salivary pellicle following chemical decontamination. Notably, only the P407 + H_2_O_2_ hydrogel did not significantly increase the amount of proteins per area after recontamination with saliva compared to a clean titanium surface, indicating its superior efficacy in maintaining surface cleanliness. Accordingly, the protein variety was also lower in the P407 + H_2_O_2_ treatment compared to the other chemical agents (Figure 3B and Table 2). As expected, there is a higher number of proteins after recontamination compared to the postdecontamination condition within each test group (Figure 3B). Some proteins were uniquely identified either postdecontamination or postrecontamination, plausibly due to varied biological interactions during protein readsorption on chemically modified surfaces with residual proteins and potential remnants of the chemical agents. When comparing the identified proteins in the experimental conditions after decontamination and after recontamination (Figures 3C,D, respectively), the majority of the proteins were shared among the conditions, but each group also had unique proteins, likely caused by the different chemical agents and surface chemistry. The ability of a chemical agent to selectively modulate the proteomic profile has also been observed in Table 2, showing the specific proteins affected by each treatment. Particularly, the chemical agents exhibited a similar effect on the selected proteins associated with bacterial adhesion, with the exception of α-amylase 2B, which was detected after decontamination with H_2_O_2_ but not with the other tested agents.

The PCA plot (Figure 4) and heat map (Figure 5) revealed distinct patterns for decontaminated surfaces, recontaminated surfaces and salivary pellicle, and pooled saliva. Notably, decontaminated surfaces exhibit broader clustering, plausibly influenced by variations in cleaning efficiency and the chemical agents used. However, after recontamination, the experimental conditions cluster closely with the salivary pellicle, suggesting consistent protein readsorption behavior. In contrast, the pooled saliva presents a different proteomic profile compared to both the experimental conditions and the salivary pellicle. Distinct differences in the proteomic profiles of the salivary pellicle and pooled saliva have been previously observed.^60^ Additionally, the volcano plots in Figure 6 highlighted that while a subset of proteins was more abundantly present after decontamination, most proteins were significantly enriched postrecontamination, aligning with the higher protein concentration following recontamination (Figure 2). Particularly, a lower number of differentially adsorbed proteins was observed in the NaOCl + AA experimental condition, indicating that most features remain at stable levels.

Despite efforts to limit pellicle recontamination on a conditioned surface in a clinical setting, saliva will most likely reintroduce proteins to the treated site. Hence, to better understand the potential biological response of the surface, in Figure 7, we analyzed the data after recontamination with saliva. The P407 hydrogel exhibited the highest enrichment in focal adhesion-related proteins, suggesting a strong potential for supporting cell attachment. The P407 + H_2_O_2_ hydrogel showed notable enrichment in integrin and cadherin binding, but it lacked proteins related to focal adhesion. This was consistent with the trend of lower protein adsorption per area (μg/mm^2^) observed with the P407 + H_2_O_2_ hydrogel after recontamination (Figure 2), which likely caused more individual proteins to fall below the detection threshold in mass spectrometry. These results align with previous findings^61^ where an OsseoSpeed-like titanium surface treated with a P407 hydrogel supported the adhesion and proliferation of MC3T3-E1 preosteoblast, human gingival fibroblast (HGF), and bone marrow mesenchymal stem cell (hBMSC). The beneficial effect of P407 was reduced by the addition of H_2_O_2_, particularly toward hBMSCs, while NaOCl + AA inhibited all the three tested cell types. However, the model of the study did not include a salivary pellicle. Hence, we have conducted cell adhesion experiments using HGFs (Figure 8) and hBMSCs (Figure 9) across the experimental conditions after recontamination with saliva. HGFs are key to produce and reorganize collagen fibers during the wound healing process, functioning together with epithelial cells to support soft tissue integration at the abutment level.^62^ Mesenchymal stem cells are recruited to the implant site in response to local cytokines and growth factors, where they initiate early bone healing by upregulating osteogenic markers.^63^ With both HGFs and hBMSCs, a pellicle-coated surface significantly reduced the number of viable cells and their adhesion compared to a clean titanium surface.

NaOCl + AA consistently showed a lower number of HGFs than the other experimental conditions, while vinculin intensity remained consistent over time, indicating poor initial but stable adhesion.

At first, P407 and P407 + H_2_O_2_ both promoted a higher HGF number compared to the salivary pellicle. However, vinculin intensity decreased over time when using P407, whereas it increased with P407 + H_2_O_2_. This indicates that P407 + H_2_O_2_ provides rapid and progressively more stable attachment. At 120 min, HGFs in the P407 and P407 + H_2_O_2_ groups spread similarly to those on the clean titanium surface. Comparatively, H_2_O_2_ alone supported initial cell adhesion with a strong vinculin signal at 30 min, which was lowered by 120 min. This decrease in vinculin intensity over time was accompanied by a reduction in viable cells, as evidenced by an increasing number of nuclei lacking surrounding cytoskeletal structures. This suggests that while H_2_O_2_ may initially promote attachment, it may also induce cytotoxic effects that compromise cell viability and stability over time.

With human bone marrow mesenchymal stem cells, H_2_O_2_ initially resulted in a higher cell number compared to the salivary pellicle group; however, its cell count declined significantly over time relative to the clean titanium surface. Similarly, NaOCl + AA consistently showed a lower number of hBMSCs compared to the other conditions. In contrast, P407 and P407 + H_2_O_2_ led to significantly higher hBMSC numbers - P407 at both 30 and 60 min, and P407 + H_2_O_2_ at 60 min only. By 120 min, no significant differences were observed between these treatments and either control group. Regarding vinculin intensity, all treatment groups except P407 + H_2_O_2_ showed significantly lower values than the clean titanium surface at early time points. However, vinculin intensity increased across all groups over time, resulting in no statistically significant differences by 120 min, indicating improved adhesion.

Overall, treatment with H_2_O_2_ and NaOCl appears to impair early attachment of both HGFs and hBMSCs, whereas Poloxamer 407-based hydrogels exhibit a beneficial effect. These findings also highlight that biological responses cannot be reliably predicted solely through KEGG and REACTOME pathway enrichment analyses, which suggested that P407 + H_2_O_2_ lacked proteins related to focal adhesion. While such analyses provide valuable indications, numerous additional factors, such as surface chemistry, surface charge, and hydrophilicity, among others, also significantly influence cell adhesion beyond the composition of pellicle proteins.

The present findings offer insights into potential surface responses to the chemical treatment, but the biological outcome ultimately depends on multiple factors, including patient variability, implant surface properties, and clinical handling techniques. Hence, *in vivo* and clinical studies are necessary to validate these *in vitro* observations. The saliva of patients affected by periodontal and peri-implant diseases has a different composition than that of healthy individuals. For instance, interleukin 1β (IL-1β) and matrix metalloproteinase-8 (MMP-8) have been identified as biomarkers of periodontal diseases.^64^ Additionally, increased serum albumin and immunoglobulin levels and a lower amount of cystatin have been found in the saliva of periodontal patients.^65^ Moreover, previous research has reported that protein adsorption depends on the titanium dental implant surface composition and properties.^43,44,66^ In clinical practice, maintaining effective suction of the treated peri-implant site is crucial to minimizing saliva recontamination. Research has demonstrated modified surface properties, particularly on originally hydrophilic surfaces, and decreased local cell viability after exposure to saliva,^11,15,67^ aligning with our findings. Moreover, saliva containing pathogenic bacteria has been shown to increase bacterial metabolism,^11^ which is particularly alarming as patients with periodontal and peri-implant diseases experience dysbiosis of the oral microbiota. Overall, our results demonstrate that the P407 + H_2_O_2_ hydrogel exhibits the strongest decontamination effect among the tested chemical agents, limiting contamination by saliva and promoting cell attachment. This is consistent with previous studies showing that, compared to other chemical decontamination products such as a P407 hydrogel alone, hydrogen peroxide, or a NaOCl + AA hydrogel, the P407 + H_2_O_2_ combination results in reduced carbon contamination, improved hydrophilicity, and lower bacterial count in a biofilm model comprising *S. oralis*, *F. nucleatum*, and *A. actinomycetemcomitans*.^68,69^

The present results also indicate that the choice of decontamination agent affects the proteomic profile of the acquired salivary pellicle and cell adhesion. Such changes may, in turn, influence bacterial colonization dynamics and long-term tissue integration. A similar study on pellicle removal from polished titanium surfaces found that surfactants are more effective than hydrophilic solutions.^70^ This further supports the use of surfactants, such as the P407 hydrogels used in this study, as chemical decontamination agents, potentially more effective than widely used hydrogen peroxide and sodium hypochlorite hydrophilic solutions.

However, this study has some limitations that should be considered, including variability in saliva composition between donors and throughout the day, the short incubation time potentially affecting adsorption, and possible underestimation of protein content due to incomplete protein desorption. Furthermore, the study only focuses on OsseoSpeed titanium dental implant surfaces, and further research is needed using other, alternative surface materials.

## 5 Conclusions

The present study aimed to evaluate the impact of chemical decontamination on the proteomic profile of the acquired salivary pellicle and cell adhesion on OsseoSpeed-like titanium dental implant surfaces using a dental pellicle model. Four chemical decontamination agents were tested: hydrogen peroxide (H_2_O_2_), Poloxamer 407 (P407), P407 + H_2_O_2_ and sodium hypochlorite + amino acids (NaOCl + AA), examining their effects after chemical decontamination and subsequent recontamination with saliva.

Core salivary proteins - mucins, amylase, immunoglobulins, cystatins, and lactotransferrin - persisted across all surfaces, indicating a strong affinity for titanium. The concentration of adsorbed protein on the surface was reduced after decontamination with all treatments except NaOCl + AA; additionally, only the P407 + H_2_O_2_ hydrogel effectively limited recontamination. Each chemical agent produced a different proteomic profile postdecontamination and postrecontamination, highlighting their selective impact. Gene ontology revealed the highest enrichment in integrin- and cadherin-binding proteins with P407 + H_2_O_2_. Lastly, P407 and P407 + H_2_O_2_ were the chemical agents promoting the highest HGF and hBMSCs count, with progressively more stable adhesion. Comparatively, H_2_O_2_ and NaOCl + AA exhibited lower numbers of HGF and hBMSCs.

Overall, we demonstrated that different chemical decontamination agents distinctly affect the salivary pellicle proteome. P407 + H_2_O_2_ demonstrated the strongest decontamination effect, allowing efficient removal of the salivary pellicle and limiting surface recontamination compared to the other chemical agents. Additionally, P407 and P407 + H_2_O_2_ promoted initial cell attachment with progressively improved adhesion.

Future research should investigate different implant surfaces, combined decontamination strategies (e.g., mechanical and chemical) and validate the findings through *in vivo* and clinical studies to account for the complexity of the oral environment and patient-specific factors, ultimately confirming their clinical relevance.

## Supplementary Material



## Notes

The list of proteins identified in each experimental condition (PDF)
