# Supplementary material for: A Comparative Study of the Impact of Chemical Debridement Products on the Proteomic Profile of the Salivary Pellicle and Cell Adhesion on OsseoSpeed Titanium Dental Implant Surfaces
Source: ACS Appl Mater Interfaces. 2025 Sep 18;17(39):54579–92. doi: 10.1021/acsami.5c14570 (PMC12492328; doi:10.1021/acsami.5c14570)
Supplement: Supplementary file 1 [file am5c14570_si_001.pdf]

Supporting Information

A Comparative Study of the Impact of Chemical Debridement Products on the Proteomic Profile of the Salivary Pellicle and Cell Adhesion on OsseoSpeed® Titanium Dental Implant Surfaces

Angela De Lauretis[1,2], Qiang Wang[1], Marco Santacroce[1,3], Bernd Thiede[4], Qianli Ma[1], Ståle Petter Lyngstadaas[1], Jan Eirik Ellingsen[5], Dirk Linke[4], Håvard Jostein Haugen[1]*

1. Department of Biomaterials, Institute of Clinical Dentistry, Faculty of Dentistry, University of Oslo, 0455, Oslo, Norway

2. Corticalis AS, Oslo Science Park, Gaustadalléen 21, 0349, Oslo, Norway

3. Department of Biomedical and Dental Sciences and Morphofunctional Imaging, University of Messina, Via Consolare Valeria, 1, 98125 Messina, Italy

4. Department of Biosciences, University of Oslo, 0316, Oslo, Norway

5. Department of Prosthetics and Oral Function, Institute of Clinical Dentistry, University of Oslo, 0455, Oslo, Norway

*Corresponding author - email: h.j.haugen@odont.uio.no, mobile: +47 91641610

Table S1. Proteins identified in pooled saliva.

UniProt Accession Number	UniProt Entry Name
P02790	HEMO_HUMAN
Q9Y6U3	SCIN_HUMAN
P00390	GSHR_HUMAN
Q8WWY7	WFD12_HUMAN
Q8NCL4	GALT6_HUMAN
Q6ZN66	GBP6_HUMAN
P14923	PLAK_HUMAN
Q3LXA3	TKFC_HUMAN
P15309	PPAP_HUMAN
Q9BVM4	GGACT_HUMAN
Q8NCC3	PAG15_HUMAN
O00754	MA2B1_HUMAN
P04406	G3P_HUMAN
Q8NFZ8	CADM4_HUMAN
Q9NVS9	PNPO_HUMAN
A0A075B6K4	LV310_HUMAN
P06737	PYGL_HUMAN
O60814	H2B1K_HUMAN
P07437	TBB5_HUMAN
A0A087WSX0	LV545_HUMAN
A6NDG6	PGP_HUMAN
P09012	SNRPA_HUMAN
P25685	DNJB1_HUMAN
P51149	RAB7A_HUMAN
Q96G03	PGM2_HUMAN
P26583	HMGB2_HUMAN
P04114	APOB_HUMAN
P30085	KCY_HUMAN
P01033	TIMP1_HUMAN
P48163	MAOX_HUMAN
P19878	NCF2_HUMAN
Q9GZS9	CHST5_HUMAN
P22735	TGM1_HUMAN
P01718	LV327_HUMAN
P13987	CD59_HUMAN
P26599	PTBP1_HUMAN
Q96DR5	BPIA2_HUMAN
A0A0C4DH24	KV621_HUMAN
P14735	IDE_HUMAN
Q92841	DDX17_HUMAN
Q6MZM0	HPHL1_HUMAN
P19957	ELAF_HUMAN
P11215	ITAM_HUMAN
Q5TFE4	NT5D1_HUMAN
Q86X29	LSR_HUMAN
Q9NX46	ADPRS_HUMAN
Q96IU4	ABHEB_HUMAN
P26022	PTX3_HUMAN
P29401	TKT_HUMAN

P13489	RINI_HUMAN
P05164	PERM_HUMAN
P07858	CATB_HUMAN
Q07960	RHG01_HUMAN
Q96GG9	DCNL1_HUMAN
P04083	ANXA1_HUMAN
P68036	UB2L3_HUMAN
Q12792	TWF1_HUMAN
P32320	CDD_HUMAN
O60568	PLOD3_HUMAN
Q9NZH7	IL36B_HUMAN
P01624	KV315_HUMAN
Q96L46	CPNS2_HUMAN
P61160	ARP2_HUMAN
P10909	CLUS_HUMAN
Q9UNW1	MINP1_HUMAN
P80723	BASP1_HUMAN
Q9HC38	GLOD4_HUMAN
Q6UX06	OLFM4_HUMAN
P04004	VTNC_HUMAN
Q5D862	FILA2_HUMAN
P60660	MYL6_HUMAN
P35754	GLRX1_HUMAN
P26639	SYTC_HUMAN
P63000	RAC1_HUMAN
P60709	ACTB_HUMAN
P17931	LEG3_HUMAN
P28482	MK01_HUMAN
Q9NUQ9	CYRIB_HUMAN
P28066	PSA5_HUMAN
P21926	CD9_HUMAN
P07737	PROF1_HUMAN
O43708	MAAI_HUMAN
Q15782	CH3L2_HUMAN
P11142	HSP7C_HUMAN
P59665	DEF1_HUMAN
P48637	GSHB_HUMAN
Q9Y617	SERC_HUMAN
P0DTE7	AMY1B_HUMAN
Q08AI8	MB214_HUMAN
P55000	SLUR1_HUMAN
P04080	CYTB_HUMAN
P05107	ITB2_HUMAN
A0A0C4DH36	HV338_HUMAN
O43240	KLK10_HUMAN
P41250	GARS_HUMAN
Q07654	TFF3_HUMAN
P15692	VEGFA_HUMAN
Q01518	CAP1_HUMAN
P63010	AP2B1_HUMAN

P04155	TFF1_HUMAN
Q9UBH0	I36RA_HUMAN
Q14697	GANAB_HUMAN
Q14019	COTL1_HUMAN
P04217	A1BG_HUMAN
O75695	XRP2_HUMAN
P08185	CBG_HUMAN
O15145	ARPC3_HUMAN
P80188	NGAL_HUMAN
Q15843	NEDD8_HUMAN
O14773	TPP1_HUMAN
P15924	DESP_HUMAN
Q13838	DX39B_HUMAN
P00558	PGK1_HUMAN
O95479	G6PE_HUMAN
Q15063	POSTN_HUMAN
Q99439	CNN2_HUMAN
Q99536	VAT1_HUMAN
P61158	ARP3_HUMAN
P08311	CATG_HUMAN
Q86SF2	GALT7_HUMAN
A0A0B4J1X8	HV343_HUMAN
Q9GZM7	TINAL_HUMAN
P22528	SPR1B_HUMAN
Q7Z4W1	DCXR_HUMAN
P22392	NDKB_HUMAN
Q6ZMR5	TM11A_HUMAN
P08571	CD14_HUMAN
P28070	PSB4_HUMAN
P12110	CO6A2_HUMAN
P01834	IGKC_HUMAN
P34096	RNAS4_HUMAN
Q9NS68	TNR19_HUMAN
P25787	PSA2_HUMAN
P00352	AL1A1_HUMAN
Q96PD5	PGRP2_HUMAN
Q6UX73	CP089_HUMAN
P22234	PUR6_HUMAN
P06396	GELS_HUMAN
P23083	HV102_HUMAN
P25815	S100P_HUMAN
P00751	CFAB_HUMAN
P15144	AMPN_HUMAN
A0A0B4J1V6	HV373_HUMAN
P11021	BIP_HUMAN
Q06323	PSME1_HUMAN
P08493	MGP_HUMAN
Q8WXI7	MUC16_HUMAN
Q5SSG8	MUC21_HUMAN
Q01082	SPTB2_HUMAN

P15586	GNS_HUMAN
Q9NS98	SEM3G_HUMAN
P55786	PSA_HUMAN
Q13867	BLMH_HUMAN
Q9UBX5	FBLN5_HUMAN
P34932	HSP74_HUMAN
Q96MK3	FA20A_HUMAN
P04908	H2A1B_HUMAN
P28074	PSB5_HUMAN
P55327	TPD52_HUMAN
Q9UIV8	SPB13_HUMAN
P49189	AL9A1_HUMAN
P53611	PGTB2_HUMAN
P55145	MANF_HUMAN
P32119	PRDX2_HUMAN
P10619	PPGB_HUMAN
Q8IV08	PLD3_HUMAN
O15511	ARPC5_HUMAN
P04746	AMYP_HUMAN
P01861	IGHG4_HUMAN
P22079	PERL_HUMAN
P15121	ALDR_HUMAN
P62942	FKB1A_HUMAN
Q9UL52	TM11E_HUMAN
Q9HB71	CYBP_HUMAN
Q02413	DSG1_HUMAN
P55263	ADK_HUMAN
P01817	HV205_HUMAN
P02765	FETUA_HUMAN
P00736	C1R_HUMAN
P07602	SAP_HUMAN
P48594	SPB4_HUMAN
Q8WWA0	ITLN1_HUMAN
P03950	ANGI_HUMAN
P01034	CYTC_HUMAN
P54819	KAD2_HUMAN
A0A0B4J1Y9	HV372_HUMAN
P68032	ACTC_HUMAN
P00739	HPTR_HUMAN
Q14914	PTGR1_HUMAN
P23284	PPIB_HUMAN
Q6UX71	PXDC2_HUMAN
Q86VP6	CAND1_HUMAN
Q9UEY8	ADDG_HUMAN
P01714	LV319_HUMAN
P27216	ANX13_HUMAN
Q15008	PSMD6_HUMAN
P61769	B2MG_HUMAN
P02763	A1AG1_HUMAN
P99999	CYC_HUMAN

P07108	ACBP_HUMAN
P62258	1433E_HUMAN
P19823	ITIH2_HUMAN
P62906	RL10A_HUMAN
P02679	FIBG_HUMAN
P13010	XRCC5_HUMAN
P02768	ALBU_HUMAN
P26447	S10A4_HUMAN
P01701	LV151_HUMAN
Q92882	OSTF1_HUMAN
Q9H4F8	SMOC1_HUMAN
P24158	PRTN3_HUMAN
Q9ULZ3	ASC_HUMAN
P06310	KV230_HUMAN
P20930	FILA_HUMAN
P05090	APOD_HUMAN
P16885	PLCG2_HUMAN
P08195	4F2_HUMAN
P37837	TALDO_HUMAN
Q04760	LGUL_HUMAN
P58499	FAM3B_HUMAN
Q14515	SPRL1_HUMAN
A0A0C4DH34	HV428_HUMAN
O95837	GNA14_HUMAN
Q6YHK3	CD109_HUMAN
Q9Y3C8	UFC1_HUMAN
P49788	TIG1_HUMAN
O00584	RNT2_HUMAN
P36871	PGM1_HUMAN
Q9HB07	MYG1_HUMAN
P01031	CO5_HUMAN
P40763	STAT3_HUMAN
Q6ZVX7	FBX50_HUMAN
P31150	GDIA_HUMAN
P18510	IL1RA_HUMAN
O94985	CSTN1_HUMAN
P03952	KLKB1_HUMAN
P00441	SODC_HUMAN
Q92484	ASM3A_HUMAN
P01619	KV320_HUMAN
Q9BW91	NUDT9_HUMAN
Q9UGM3	DMBT1_HUMAN
O43707	ACTN4_HUMAN
Q14508	WFDC2_HUMAN
Q8N474	SFRP1_HUMAN
P02647	APOA1_HUMAN
O75368	SH3L1_HUMAN
P12830	CADH1_HUMAN
P0DOY3	IGLC3_HUMAN
P16035	TIMP2_HUMAN

P00450	CERU_HUMAN
P27361	MK03_HUMAN
A0A075B6S5	KV127_HUMAN
P26572	MGAT1_HUMAN
P09382	LEG1_HUMAN
P04839	CY24B_HUMAN
Q9UBQ5	EIF3K_HUMAN
A0A0B4J1X5	HV374_HUMAN
O00462	MANBA_HUMAN
O15144	ARPC2_HUMAN
P02042	HBD_HUMAN
P11940	PABP1_HUMAN
Q9NYQ8	FAT2_HUMAN
Q9UJC5	SH3L2_HUMAN
P80303	NUCB2_HUMAN
P02749	APOH_HUMAN
O14638	ENPP3_HUMAN
P13639	EF2_HUMAN
P01715	LV301_HUMAN
O95865	DDAH2_HUMAN
Q14103	HNRPD_HUMAN
Q9BWS9	CHID1_HUMAN
P15531	NDKA_HUMAN
Q9H4M9	EHD1_HUMAN
P61604	CH10_HUMAN
E9PAV3	NACAM_HUMAN
P25789	PSA4_HUMAN
P61586	RHOA_HUMAN
O00560	SDCB1_HUMAN
Q96DA0	PAUF_HUMAN
P17213	BPI_HUMAN
P22894	MMP8_HUMAN
P84077	ARF1_HUMAN
P61019	RAB2A_HUMAN
P07355	ANXA2_HUMAN
P09758	TACD2_HUMAN
P08697	A2AP_HUMAN
P07686	HEXB_HUMAN
P17050	NAGAB_HUMAN
Q9UL46	PSME2_HUMAN
P40121	CAPG_HUMAN
Q92876	KLK6_HUMAN
P17174	AATC_HUMAN
P41218	MNDA_HUMAN
Q96C19	EFHD2_HUMAN
P10644	KAP0_HUMAN
Q9UBX7	KLK11_HUMAN
Q9UBR2	CATZ_HUMAN
Q9HC84	MUC5B_HUMAN
O00764	PDXK_HUMAN

O43765	SGTA_HUMAN
Q14974	IMB1_HUMAN
Q96P44	COLA1_HUMAN
P42785	PCP_HUMAN
P30046	DOPD_HUMAN
O95274	LYPD3_HUMAN
Q9NZ08	ERAP1_HUMAN
Q16378	PROL4_HUMAN
P06733	ENOA_HUMAN
Q86UX7	URP2_HUMAN
P04439	HLAA_HUMAN
P15328	FOLR1_HUMAN
P05546	HEP2_HUMAN
Q00722	PLCB2_HUMAN
Q13217	DNJC3_HUMAN
Q13231	CHIT1_HUMAN
P41091	IF2G_HUMAN
Q9UKR3	KLK13_HUMAN
P01859	IGHG2_HUMAN
P32926	DSG3_HUMAN
Q9NY97	B3GN2_HUMAN
P00746	CFAD_HUMAN
P62993	GRB2_HUMAN
P06753	TPM3_HUMAN
P10153	RNAS2_HUMAN
O14818	PSA7_HUMAN
Q9GZZ8	LACRT_HUMAN
P08240	SRPRA_HUMAN
Q15172	2A5A_HUMAN
P25311	ZA2G_HUMAN
Q86VR7	VS10L_HUMAN
Q7Z4H8	PLGT3_HUMAN
A0M8Q6	IGLC7_HUMAN
P36952	SPB5_HUMAN
P08476	INHBA_HUMAN
P62328	TYB4_HUMAN
P0CF74	IGLC6_HUMAN
P11684	UTER_HUMAN
A0A075B6H9	LV469_HUMAN
Q16610	ECM1_HUMAN
P33176	KINH_HUMAN
P12081	HARS1_HUMAN
P09429	HMGB1_HUMAN
P06312	KV401_HUMAN
A6NIZ1	RP1BL_HUMAN
P01019	ANGT_HUMAN
P28838	AMPL_HUMAN
P35579	MYH9_HUMAN
Q96C90	PP14B_HUMAN
P54727	RD23B_HUMAN

Q9UBG3	CRNN_HUMAN
P08238	HS90B_HUMAN
A0A0C4DH42	HV366_HUMAN
O15247	CLIC2_HUMAN
P01009	A1AT_HUMAN
Q96BQ1	FAM3D_HUMAN
Q8NBJ4	GOLM1_HUMAN
P52209	6PGD_HUMAN
O95166	GBRAP_HUMAN
O43490	PROM1_HUMAN
Q16881	TRXR1_HUMAN
Q96LB8	PGRP4_HUMAN
P0DMV8	HS71A_HUMAN
Q9BQR3	PRS27_HUMAN
O14964	HGS_HUMAN
P22532	SPR2D_HUMAN
P23280	CAH6_HUMAN
P02766	TTHY_HUMAN
P54802	ANAG_HUMAN
A0A075B6S2	KVD29_HUMAN
Q2M2H8	MGAL_HUMAN
P08758	ANXA5_HUMAN
P61086	UBE2K_HUMAN
Q9NZH8	IL36G_HUMAN
Q13630	FCL_HUMAN
P30838	AL3A1_HUMAN
O75347	TBCA_HUMAN
P06702	S10A9_HUMAN
P07900	HS90A_HUMAN
P07384	CAN1_HUMAN
Q8NCW5	NNRE_HUMAN
P49862	KLK7_HUMAN
Q9BRR6	ADPGK_HUMAN
Q13510	ASAH1_HUMAN
Q15435	PP1R7_HUMAN
O00115	DNS2A_HUMAN
Q8N4A0	GALT4_HUMAN
Q92597	NDRG1_HUMAN
P04632	CPNS1_HUMAN
Q16769	QPCT_HUMAN
P49913	CAMP_HUMAN
P02753	RET4_HUMAN
P62253	UB2G1_HUMAN
P09228	CYTT_HUMAN
Q04446	GLGB_HUMAN
P49720	PSB3_HUMAN
P12429	ANXA3_HUMAN
P30041	PRDX6_HUMAN
Q9Y287	ITM2B_HUMAN
P20160	CAP7_HUMAN

Q8IXK2	GLT12_HUMAN
P01824	HV439_HUMAN
Q9HAT2	SIAE_HUMAN
Q9NZH0	GPC5B_HUMAN
P06870	KLK1_HUMAN
O14980	XPO1_HUMAN
O95881	TXD12_HUMAN
P01860	IGHG3_HUMAN
Q08380	LG3BP_HUMAN
Q9Y646	CBPQ_HUMAN
O00160	MYO1F_HUMAN
P36955	PEDF_HUMAN
P31948	STIP1_HUMAN
Q15833	STXB2_HUMAN
P12268	IMDH2_HUMAN
P01023	A2MG_HUMAN
P60900	PSA6_HUMAN
P35573	GDE_HUMAN
P49773	HINT1_HUMAN
O75882	ATRN_HUMAN
Q9H0B8	CRLD2_HUMAN
P08174	DAF_HUMAN
P01602	KV105_HUMAN
P31151	S10A7_HUMAN
Q7L2H7	EIF3M_HUMAN
P52566	GDIR2_HUMAN
Q92896	GSLG1_HUMAN
Q8WUA7	TB22A_HUMAN
P08582	TRFM_HUMAN
P05109	S10A8_HUMAN
P60174	TPIS_HUMAN
Q06828	FMOD_HUMAN
Q9Y4L1	HYOU1_HUMAN
Q8TAX7	MUC7_HUMAN
P00918	CAH2_HUMAN
P05089	ARGI1_HUMAN
P09467	F16P1_HUMAN
P54803	GALC_HUMAN
Q00169	PIPNA_HUMAN
P01772	HV333_HUMAN
P68431	H31_HUMAN
P34059	GALNS_HUMAN
P07358	CO8B_HUMAN
P21128	ENDOU_HUMAN
Q9P1F3	ABRAL_HUMAN
P60903	S10AA_HUMAN
Q9NRB3	CHSTC_HUMAN
Q9UJJ9	GNPTG_HUMAN
Q9NQ84	GPC5C_HUMAN
Q9UKK9	NUDT5_HUMAN

P26885	FKBP2_HUMAN
P00734	THRB_HUMAN
Q9UN36	NDRG2_HUMAN
O15335	CHAD_HUMAN
P13796	PLSL_HUMAN
P14598	NCF1_HUMAN
P58546	MTPN_HUMAN
P20061	TCO1_HUMAN
P02545	LMNA_HUMAN
P01857	IGHG1_HUMAN
Q6P5S2	LEG1H_HUMAN
Q07283	TRHY_HUMAN
P48723	HSP13_HUMAN
P03973	SLPI_HUMAN
Q10567	AP1B1_HUMAN
Q9UJ70	NAGK_HUMAN
Q9GZN4	BSSP4_HUMAN
P01871	IGHM_HUMAN
P15104	GLNA_HUMAN
Q06830	PRDX1_HUMAN
P30086	PEBP1_HUMAN
Q96S96	PEBP4_HUMAN
Q9UJ68	MSRA_HUMAN
P39687	AN32A_HUMAN
P35321	SPR1A_HUMAN
O14732	IMPA2_HUMAN
Q15404	RSU1_HUMAN
P10643	CO7_HUMAN
P02750	A2GL_HUMAN
Q7Z406	MYH14_HUMAN
P09958	FURIN_HUMAN
Q5T750	KPLCE_HUMAN
Q96HE7	ERO1A_HUMAN
P01764	HV323_HUMAN
Q9HB40	RISC_HUMAN
P63104	1433Z_HUMAN
Q3YEC7	RABL6_HUMAN
P61026	RAB10_HUMAN
P17405	ASM_HUMAN
P17900	SAP3_HUMAN
P01037	CYTN_HUMAN
P31947	1433S_HUMAN
P05155	IC1_HUMAN
P48739	PIPNB_HUMAN
P30101	PDIA3_HUMAN
O95373	IPO7_HUMAN
P06454	PTMA_HUMAN
P27487	DPP4_HUMAN
O00602	FCN1_HUMAN
P11413	G6PD_HUMAN

Q9Y490	TLN1_HUMAN
P01877	IGHA2_HUMAN
O60610	DIAP1_HUMAN
P53396	ACLY_HUMAN
Q6PCB0	VWA1_HUMAN
Q96TA1	NIBA2_HUMAN
Q9H4A4	AMPB_HUMAN
Q14435	GALT3_HUMAN
P40926	MDHM_HUMAN
P12273	PIP_HUMAN
P22676	CALB2_HUMAN
P52790	HXK3_HUMAN
Q6UW32	IGFL1_HUMAN
Q9BW30	TPPP3_HUMAN
Q9BYE4	SPR2G_HUMAN
P01040	CYTA_HUMAN
P17655	CAN2_HUMAN
Q04118	PRB3_HUMAN
O95867	LY66C_HUMAN
P69905	HBA_HUMAN
Q02809	PLOD1_HUMAN
P01780	HV307_HUMAN
P13284	GILT_HUMAN
P20290	BTF3_HUMAN
Q9UKR0	KLK12_HUMAN
Q9BS26	ERP44_HUMAN
Q9UM21	MGT4A_HUMAN
P19971	TYPH_HUMAN
Q00610	CLH1_HUMAN
P50552	VASP_HUMAN
P01042	KNG1_HUMAN
P12109	CO6A1_HUMAN
P02675	FIBB_HUMAN
P02746	C1QB_HUMAN
P08670	VIME_HUMAN
O95841	ANGL1_HUMAN
P01762	HV311_HUMAN
Q969H8	MYDGF_HUMAN
P08603	CFAH_HUMAN
Q9NZT1	CALL5_HUMAN
P04233	HG2A_HUMAN
Q14624	ITIH4_HUMAN
P14780	MMP9_HUMAN
Q5JWF2	GNAS1_HUMAN
P01601	KVD16_HUMAN
A0A075B6R9	KVD24_HUMAN
P40394	ADH7_HUMAN
P43652	AFAM_HUMAN
Q02487	DSC2_HUMAN
Q08554	DSC1_HUMAN

Q6P4A8	PLBL1_HUMAN
Q05707	COEA1_HUMAN
P0DJI8	SAA1_HUMAN
P02760	AMBP_HUMAN
Q9UHA7	IL36A_HUMAN
Q92520	FAM3C_HUMAN
Q8NES3	LFNG_HUMAN
Q15365	PCBP1_HUMAN
P61981	1433G_HUMAN
P08246	ELNE_HUMAN
P68871	HBB_HUMAN
P51858	HDGF_HUMAN
Q9Y6R7	FCGBP_HUMAN
P21217	FUT3_HUMAN
P02788	TRFL_HUMAN
P01876	IGHA1_HUMAN
P15311	EZRI_HUMAN
P40189	IL6RB_HUMAN
P13693	TCTP_HUMAN
Q13616	CUL1_HUMAN
Q86SQ4	AGRG6_HUMAN
P06681	CO2_HUMAN
P18206	VINC_HUMAN
O00244	ATOX1_HUMAN
O43488	ARK72_HUMAN
P01782	HV309_HUMAN
P04040	CATA_HUMAN
P49327	FAS_HUMAN
P48147	PPCE_HUMAN
Q9BRA2	TXD17_HUMAN
P28325	CYTD_HUMAN
O60218	AK1BA_HUMAN
A0A075B6K5	LV39_HUMAN
A0A075B6I0	LV861_HUMAN
P07954	FUMH_HUMAN
P07195	LDHB_HUMAN
O75594	PGRP1_HUMAN
P59998	ARPC4_HUMAN
P01593	KVD33_HUMAN
O43866	CD5L_HUMAN
P05156	CFAI_HUMAN
P29373	RABP2_HUMAN
Q8N4F0	BPIB2_HUMAN
Q15631	TSN_HUMAN
P28676	GRAN_HUMAN
O00391	QSOX1_HUMAN
P15559	NQO1_HUMAN
P30040	ERP29_HUMAN
P68366	TBA4A_HUMAN
Q9NQ38	ISK5_HUMAN

Q9NY33	DPP3_HUMAN
P30044	PRDX5_HUMAN
P16278	BGAL_HUMAN
P27824	CALX_HUMAN
Q05315	LEG10_HUMAN
P0DP57	SLUR2_HUMAN
P14207	FOLR2_HUMAN
P01833	PIGR_HUMAN
P15153	RAC2_HUMAN
Q13232	NDK3_HUMAN
P58062	ISK7_HUMAN
Q01459	DIAC_HUMAN
P62879	GBB2_HUMAN
Q9HA64	KT3K_HUMAN
Q12906	ILF3_HUMAN
O94760	DDAH1_HUMAN
Q06124	PTN11_HUMAN
P06744	G6PI_HUMAN
Q13438	OS9_HUMAN
P01036	CYTS_HUMAN
P09960	LKHA4_HUMAN
P19961	AMY2B_HUMAN
A0A0B4J1U7	HV601_HUMAN
P61626	LYSC_HUMAN
Q16706	MA2A1_HUMAN
Q99685	MGLL_HUMAN
P12814	ACTN1_HUMAN
Q9H0W9	CK054_HUMAN
P31025	LCN1_HUMAN
Q08188	TGM3_HUMAN
P31146	COR1A_HUMAN
P27169	PON1_HUMAN
Q16787	LAMA3_HUMAN
P01700	LV147_HUMAN
P00750	TPA_HUMAN
P46779	RL28_HUMAN
P47929	LEG7_HUMAN
P61088	UBE2N_HUMAN
P43490	NAMPT_HUMAN
P0DP23	CALM1_HUMAN
P52565	GDIR1_HUMAN
Q9UMX0	UBQL1_HUMAN
A0A0B4J1V0	HV315_HUMAN
Q53FA7	QORX_HUMAN
Q24JP5	T132A_HUMAN
Q9BYC5	FUT8_HUMAN
A0A0J9YX35	HV64D_HUMAN
P09525	ANXA4_HUMAN
Q9H299	SH3L3_HUMAN
Q15582	BGH3_HUMAN

P01721	LV657_HUMAN
P23526	SAHH_HUMAN
P62136	PP1A_HUMAN
P48595	SPB10_HUMAN
P01591	IGJ_HUMAN
O15143	ARC1B_HUMAN
Q9Y446	PKP3_HUMAN
Q8TDL5	BPIB1_HUMAN
P43251	BTD_HUMAN
O75503	CLN5_HUMAN
O75874	IDHC_HUMAN
Q14677	EPN4_HUMAN
P31949	S10AB_HUMAN
Q6BCY4	NB5R2_HUMAN
P14618	KPYM_HUMAN
P0DTE1	HV383_HUMAN
P55268	LAMB2_HUMAN
P13667	PDIA4_HUMAN
Q96NY8	NECT4_HUMAN
Q96RM1	SPR2F_HUMAN
O43776	SYNC_HUMAN
Q9NSC7	SIA7A_HUMAN
Q14CN2	CLCA4_HUMAN
P13797	PLST_HUMAN
P31946	1433B_HUMAN
P02774	VTDB_HUMAN
Q9NRA1	PDGFC_HUMAN
P63167	DYL1_HUMAN
Q96FQ6	S10AG_HUMAN
Q9Y376	CAB39_HUMAN
O60259	KLK8_HUMAN
Q01469	FABP5_HUMAN
P61978	HNRPK_HUMAN
Q86T26	TM11B_HUMAN
Q13183	S13A2_HUMAN
O75223	GGCT_HUMAN
P16152	CBR1_HUMAN
O60293	ZC3H1_HUMAN
Q16851	UGPA_HUMAN
P20618	PSB1_HUMAN
P19652	A1AG2_HUMAN
P22792	CPN2_HUMAN
O75888	TNF13_HUMAN
Q9H173	SIL1_HUMAN
Q13813	SPTN1_HUMAN
P06731	CEAM5_HUMAN
A0A0B4J1Y8	LV949_HUMAN
P28072	PSB6_HUMAN
P11279	LAMP1_HUMAN
P07738	PMGE_HUMAN

Q9NYU2	UGGG1_HUMAN
P05067	A4_HUMAN
P00492	HPRT_HUMAN
P20810	ICAL_HUMAN
Q8WUM4	PDC6I_HUMAN
P27797	CALR_HUMAN
P24821	TENA_HUMAN
Q12841	FSTL1_HUMAN
O75326	SEM7A_HUMAN
P60953	CDC42_HUMAN
P01743	HV146_HUMAN
A0A075B6K2	LV312_HUMAN
P01024	CO3_HUMAN
P22314	UBA1_HUMAN
P14625	ENPL_HUMAN
P22061	PIMT_HUMAN
Q96QR1	SG3A1_HUMAN
Q9NQ88	TIGAR_HUMAN
P30043	BLVRB_HUMAN
O00469	PLOD2_HUMAN
Q9UBC9	SPRR3_HUMAN
P35611	ADDA_HUMAN
O60235	TM11D_HUMAN
P80511	S10AC_HUMAN
P51993	FUT6_HUMAN
P49908	SEPP1_HUMAN
Q9H190	SDCB2_HUMAN
P80748	LV321_HUMAN
P0C0L4	CO4A_HUMAN
P25398	RS12_HUMAN
Q93008	USP9X_HUMAN
Q8WVQ1	CANT1_HUMAN
Q9UNM6	PSD13_HUMAN
P16083	NQO2_HUMAN
Q9BUF5	TBB6_HUMAN
P07339	CATD_HUMAN
Q15181	IPYR_HUMAN
Q9BS40	LXN_HUMAN
P04433	KV311_HUMAN
P46459	NSF_HUMAN
Q14574	DSC3_HUMAN
O00748	EST2_HUMAN
P29034	S10A2_HUMAN
Q9UHL4	DPP2_HUMAN
Q15417	CNN3_HUMAN
Q6XPR3	RPTN_HUMAN
A0A0C4DH67	KV108_HUMAN
A0A0J9YXX1	HV5X1_HUMAN
P19827	ITIH1_HUMAN
Q14703	MBTP1_HUMAN

P29508	SPB3_HUMAN
Q8N6Q3	CD177_HUMAN
P0C870	JMJD7_HUMAN
Q9ULV4	COR1C_HUMAN
Q99497	PARK7_HUMAN
P16070	CD44_HUMAN
Q9Y274	SIA10_HUMAN
Q04941	PLP2_HUMAN
P61916	NPC2_HUMAN
P30405	PPIF_HUMAN
P30740	ILEU_HUMAN
Q02818	NUCB1_HUMAN
Q8NFT8	DNER_HUMAN
P62937	PPIA_HUMAN
P00338	LDHA_HUMAN
P49589	SYCC_HUMAN
P01008	ANT3_HUMAN
P15289	ARSA_HUMAN
P07237	PDIA1_HUMAN
P00738	HPT_HUMAN
P27482	CALL3_HUMAN
Q9NP55	BPIA1_HUMAN
Q8NBS9	TXND5_HUMAN
O00299	CLIC1_HUMAN
P62826	RAN_HUMAN
P05543	THBG_HUMAN
P61077	UB2D3_HUMAN
P52895	AK1C2_HUMAN
P53634	CATC_HUMAN
P51888	PRELP_HUMAN
P53004	BIEA_HUMAN
P0CG47	UBB_HUMAN
O75165	DJC13_HUMAN
Q16658	FSCN1_HUMAN
P47756	CAPZB_HUMAN
P27105	STOM_HUMAN
O95336	6PGL_HUMAN
Q9Y5Z4	HEBP2_HUMAN
O75015	FCG3B_HUMAN
Q96P63	SPB12_HUMAN
P09417	DHPR_HUMAN
A0A0C4DH31	HV118_HUMAN
P09651	ROA1_HUMAN
P02751	FINC_HUMAN
P04280	PRP1_HUMAN
Q92820	GGH_HUMAN
Q9NZD2	GLTP_HUMAN
P04899	GNAI2_HUMAN
P0DTE2	HV511_HUMAN
P00491	PNPH_HUMAN

P55058	PLTP_HUMAN
O43852	CALU_HUMAN
O95164	UBL3_HUMAN
P40925	MDHC_HUMAN
P18669	PGAM1_HUMAN
P06280	AGAL_HUMAN
P21246	PTN_HUMAN
Q13885	TBB2A_HUMAN
O95834	EMAL2_HUMAN
P02671	FIBA_HUMAN
Q9Y262	EIF3L_HUMAN
O95466	FMNL1_HUMAN
A0A0C4DH38	HV551_HUMAN
P54108	CRIS3_HUMAN
P35326	SPR2A_HUMAN
P07360	CO8G_HUMAN
P46926	GNPI1_HUMAN
Q15828	CYTM_HUMAN
Q16651	PRSS8_HUMAN
P36222	CH3L1_HUMAN
P04066	FUCO_HUMAN
P49902	5NTC_HUMAN
P0CG04	IGLC1_HUMAN
P55072	TERA_HUMAN
P18085	ARF4_HUMAN
O00159	MYO1C_HUMAN
Q99935	PROL1_HUMAN
P26038	MOES_HUMAN
O75131	CPNE3_HUMAN
Q14956	GPNMB_HUMAN
P09211	GSTP1_HUMAN
P01880	IGHD_HUMAN
A0A0B4J1V2	HV226_HUMAN
P09972	ALDOC_HUMAN
P0DP02	HVC33_HUMAN
P10599	THIO_HUMAN
P04430	KV116_HUMAN
Q9P0G3	KLK14_HUMAN
P01704	LV214_HUMAN
P00747	PLMN_HUMAN
Q13740	CD166_HUMAN
P04792	HSPB1_HUMAN
P19021	AMD_HUMAN
P16870	CBPE_HUMAN
O95436	NPT2B_HUMAN
P12956	XRCC6_HUMAN
P09668	CATH_HUMAN
Q6UWP8	SBSN_HUMAN
Q9HD89	RETN_HUMAN
P49721	PSB2_HUMAN

Q9ULC6	PADI1_HUMAN
P28799	GRN_HUMAN
P78380	OLR1_HUMAN
P07910	HNRPC_HUMAN
P46940	IQGA1_HUMAN
Q99538	LGMN_HUMAN
Q16777	H2A2C_HUMAN
O60784	TOM1_HUMAN
Q13421	MSLN_HUMAN
Q96JB3	HIC2_HUMAN
P01763	HV348_HUMAN
P51665	PSMD7_HUMAN
Q99102	MUC4_HUMAN
Q86U17	SPA11_HUMAN
Q9UKM7	MA1B1_HUMAN
P21333	FLNA_HUMAN
A8K2U0	A2ML1_HUMAN
O15217	GSTA4_HUMAN
P07996	TSP1_HUMAN
P27348	1433T_HUMAN
Q96JY6	PDLI2_HUMAN
Q14210	LY6D_HUMAN
P01717	LV325_HUMAN
P78417	GSTO1_HUMAN
P01597	KV139_HUMAN
Q13162	PRDX4_HUMAN
Q13404	UB2V1_HUMAN
P04075	ALDOA_HUMAN
P08294	SODE_HUMAN
P07711	CATL1_HUMAN
P50395	GDIB_HUMAN
P14314	GLU2B_HUMAN
P09488	GSTM1_HUMAN
P68104	EF1A1_HUMAN
Q9UBD6	RHCG_HUMAN
P62873	GBB1_HUMAN
P62805	H4_HUMAN
Q92688	AN32B_HUMAN
Q7Z7M9	GALT5_HUMAN
P01703	LV140_HUMAN
P16930	FAAA_HUMAN
O60841	IF2P_HUMAN
Q99523	SORT_HUMAN
P04062	GBA1_HUMAN
P31944	CASPE_HUMAN
P21741	MK_HUMAN
O95833	CLIC3_HUMAN
O75083	WDR1_HUMAN
Q9BXJ4	C1QT3_HUMAN
Q9Y2V2	CHSP1_HUMAN

P37802	TAGL2_HUMAN
P06703	S10A6_HUMAN
Q10472	GALT1_HUMAN
P33908	MA1A1_HUMAN
A0A0C4DH55	KVD07_HUMAN
Q6IBS0	TWF2_HUMAN
Q6UWT4	CE046_HUMAN
P23141	EST1_HUMAN
O75390	CISY_HUMAN
Q9BRF8	CPPED_HUMAN
O00204	ST2B1_HUMAN
P02787	TRFE_HUMAN
Q9UIQ6	LCAP_HUMAN
O15020	SPTN2_HUMAN
Q04637	IF4G1_HUMAN
O75976	CBPD_HUMAN
O43278	SPIT1_HUMAN
P15291	B4GT1_HUMAN
P01599	KV117_HUMAN
P25786	PSA1_HUMAN
P26641	EF1G_HUMAN
Q99574	NEUS_HUMAN
P14550	AK1A1_HUMAN
Q8TE68	ES8L1_HUMAN
O60888	CUTA_HUMAN
P51812	KS6A3_HUMAN
P98160	PGBM_HUMAN

Table S2. Proteins identified in the salivary pellicle acquired on the OsseoSpeed®-like titanium dental implant surface.

UniProt Accession Number	UniProt Entry Name
P02790	HEMO_HUMAN
P03973	SLPI_HUMAN
Q9UJ70	NAGK_HUMAN
P01871	IGHM_HUMAN
P14923	PLAK_HUMAN
Q06830	PRDX1_HUMAN
P30086	PEBP1_HUMAN
Q96S96	PEBP4_HUMAN
P15309	PPAP_HUMAN
P35321	SPR1A_HUMAN
P04406	G3P_HUMAN
P02750	A2GL_HUMAN
P09958	FURIN_HUMAN
P06737	PYGL_HUMAN
Q5T750	KPLCE_HUMAN
Q96HE7	ERO1A_HUMAN
P25705	ATPA_HUMAN
P01764	HV323_HUMAN
P07477	TRY1_HUMAN
Q96G03	PGM2_HUMAN
P63104	1433Z_HUMAN
A0A0C4DH35	HV335_HUMAN
P01033	TIMP1_HUMAN
P48163	MAOX_HUMAN
P17900	SAP3_HUMAN
P01037	CYTN_HUMAN
P31947	1433S_HUMAN
P22735	TGM1_HUMAN
O76031	CLPX_HUMAN
P13987	CD59_HUMAN
Q9Y697	NFS1_HUMAN
O75449	KTNA1_HUMAN
Q96DR5	BPIA2_HUMAN
P14174	MIF_HUMAN
Q92841	DDX17_HUMAN
P11413	G6PD_HUMAN
P01877	IGHA2_HUMAN
P29401	TKT_HUMAN
P05164	PERM_HUMAN
P07858	CATB_HUMAN
P40926	MDHM_HUMAN
Q13576	IQGA2_HUMAN
O75071	EFC14_HUMAN
P12273	PIP_HUMAN
P01040	CYTA_HUMAN
P04083	ANXA1_HUMAN
P17655	CAN2_HUMAN
P69905	HBA_HUMAN
P10909	CLUS_HUMAN

P01780	HV307_HUMAN
Q6UX06	OLFM4_HUMAN
P04004	VTNC_HUMAN
Q9UKR0	KLK12_HUMAN
Q5D862	FILA2_HUMAN
P60660	MYL6_HUMAN
P60709	ACTB_HUMAN
P17931	LEG3_HUMAN
P12109	CO6A1_HUMAN
P02675	FIBB_HUMAN
P21926	CD9_HUMAN
P07737	PROF1_HUMAN
Q15782	CH3L2_HUMAN
P11142	HSP7C_HUMAN
P59665	DEF1_HUMAN
P08670	VIME_HUMAN
Q92673	SORL_HUMAN
P0DTE7	AMY1B_HUMAN
Q9NZT1	CALL5_HUMAN
P55000	SLUR1_HUMAN
P04080	CYTB_HUMAN
Q14624	ITIH4_HUMAN
A0A0C4DH36	HV338_HUMAN
P14780	MMP9_HUMAN
A0A075B6R9	KVD24_HUMAN
P40394	ADH7_HUMAN
Q07654	TFF3_HUMAN
Q02487	DSC2_HUMAN
Q08554	DSC1_HUMAN
Q05707	COEA1_HUMAN
Q01518	CAP1_HUMAN
Q9UBH0	I36RA_HUMAN
P61981	1433G_HUMAN
Q14697	GANAB_HUMAN
P04217	A1BG_HUMAN
P08246	ELNE_HUMAN
P68871	HBB_HUMAN
P80188	NGAL_HUMAN
Q9Y6R7	FCGBP_HUMAN
P02788	TRFL_HUMAN
P01876	IGHA1_HUMAN
P15311	EZRI_HUMAN
P40189	IL6RB_HUMAN
O14773	TPP1_HUMAN
P15924	DESP_HUMAN
P61970	NTF2_HUMAN
P18206	VINC_HUMAN
P01782	HV309_HUMAN
P04040	CATA_HUMAN
O43592	XPOT_HUMAN

P61158	ARP3_HUMAN
P08311	CATG_HUMAN
Q969S9	RRF2M_HUMAN
Q9GZM7	TINAL_HUMAN
P28325	CYTD_HUMAN
O60218	AK1BA_HUMAN
Q9UKQ9	KLK9_HUMAN
P22392	NDKB_HUMAN
P08571	CD14_HUMAN
P07195	LDHB_HUMAN
P01834	IGKC_HUMAN
P48729	KC1A_HUMAN
P59998	ARPC4_HUMAN
P05156	CFAI_HUMAN
P25787	PSA2_HUMAN
P29373	RABP2_HUMAN
Q8N4F0	BPIB2_HUMAN
O00391	QSOX1_HUMAN
Q9NQ38	ISK5_HUMAN
Q9NY33	DPP3_HUMAN
P06396	GELS_HUMAN
P30044	PRDX5_HUMAN
P23083	HV102_HUMAN
P25815	S100P_HUMAN
P00751	CFAB_HUMAN
P01833	PIGR_HUMAN
P15153	RAC2_HUMAN
O14522	PTPRT_HUMAN
P11021	BIP_HUMAN
Q06323	PSME1_HUMAN
P06744	G6PI_HUMAN
P01036	CYTS_HUMAN
Q5SSG8	MUC21_HUMAN
P09960	LKHA4_HUMAN
P19961	AMY2B_HUMAN
P61626	LYSC_HUMAN
P55786	PSA_HUMAN
Q13867	BLMH_HUMAN
P12814	ACTN1_HUMAN
P31025	LCN1_HUMAN
Q08188	TGM3_HUMAN
P31146	COR1A_HUMAN
P53597	SUCA_HUMAN
P01700	LV147_HUMAN
P25929	NPY1R_HUMAN
Q9UIV8	SPB13_HUMAN
P47929	LEG7_HUMAN
P61088	UBE2N_HUMAN
P43490	NAMPT_HUMAN
P0DP23	CALM1_HUMAN

P52565	GDIR1_HUMAN
P01861	IGHG4_HUMAN
P22079	PERL_HUMAN
P62942	FKB1A_HUMAN
Q9H299	SH3L3_HUMAN
Q02413	DSG1_HUMAN
P01721	LV657_HUMAN
P01817	HV205_HUMAN
P02765	FETUA_HUMAN
P07602	SAP_HUMAN
P48594	SPB4_HUMAN
Q8WWA0	ITLN1_HUMAN
P01034	CYTC_HUMAN
P01591	IGJ_HUMAN
A0A0B4J1Y9	HV372_HUMAN
P68032	ACTC_HUMAN
Q07812	BAX_HUMAN
Q8TDL5	BPIB1_HUMAN
O75874	IDHC_HUMAN
P31949	S10AB_HUMAN
Q14914	PTGR1_HUMAN
P23284	PPIB_HUMAN
P14618	KPYM_HUMAN
P55064	AQP5_HUMAN
P61769	B2MG_HUMAN
P02763	A1AG1_HUMAN
P99999	CYC_HUMAN
P07108	ACBP_HUMAN
Q96RM1	SPR2F_HUMAN
Q14CN2	CLCA4_HUMAN
P13797	PLST_HUMAN
P31946	1433B_HUMAN
P02774	VTDB_HUMAN
P62258	1433E_HUMAN
P06865	HEXA_HUMAN
P19823	ITIH2_HUMAN
P02679	FIBG_HUMAN
P02768	ALBU_HUMAN
P26447	S10A4_HUMAN
P24158	PRTN3_HUMAN
P63261	ACTG_HUMAN
Q9ULZ3	ASC_HUMAN
Q96FQ6	S10AG_HUMAN
Q9Y376	CAB39_HUMAN
Q01469	FABP5_HUMAN
P05090	APOD_HUMAN
O75223	GGCT_HUMAN
P37837	TALDO_HUMAN
O60293	ZC3H1_HUMAN
P58499	FAM3B_HUMAN

P19652	A1AG2_HUMAN
Q14515	SPRL1_HUMAN
O00584	RNT2_HUMAN
P36871	PGM1_HUMAN
Q6ZVX7	FBX50_HUMAN
P31150	GDIA_HUMAN
P18510	IL1RA_HUMAN
P05067	A4_HUMAN
P00441	SODC_HUMAN
P00492	HPRT_HUMAN
P01619	KV320_HUMAN
P27797	CALR_HUMAN
Q9UGM3	DMBT1_HUMAN
O43707	ACTN4_HUMAN
Q14508	WFDC2_HUMAN
Q8N474	SFRP1_HUMAN
P02647	APOA1_HUMAN
O75368	SH3L1_HUMAN
P12830	CADH1_HUMAN
P35222	CTNB1_HUMAN
P00450	CERU_HUMAN
P01743	HV146_HUMAN
P01024	CO3_HUMAN
Q96QR1	SG3A1_HUMAN
Q9UBC9	SPRR3_HUMAN
O60235	TM11D_HUMAN
P80511	S10AC_HUMAN
O00462	MANBA_HUMAN
Q9UJC5	SH3L2_HUMAN
P80748	LV321_HUMAN
P80303	NUCB2_HUMAN
P0C0L4	CO4A_HUMAN
P02749	APOH_HUMAN
P13639	EF2_HUMAN
Q9BWS9	CHID1_HUMAN
P15531	NDKA_HUMAN
Q16696	CP2AD_HUMAN
P61586	RHOA_HUMAN
P07339	CATD_HUMAN
Q96DA0	PAUF_HUMAN
P17213	BPI_HUMAN
P22894	MMP8_HUMAN
P07355	ANXA2_HUMAN
Q14574	DSC3_HUMAN
O00748	EST2_HUMAN
P29034	S10A2_HUMAN
Q9UHL4	DPP2_HUMAN
Q10588	BST1_HUMAN
O75629	CREG1_HUMAN
Q9UL46	PSME2_HUMAN

P19827	ITIH1_HUMAN
P40121	CAPG_HUMAN
Q92876	KLK6_HUMAN
P29508	SPB3_HUMAN
P17174	AATC_HUMAN
P0C870	JMJD7_HUMAN
Q6NUJ1	SAPL1_HUMAN
Q9UBX7	KLK11_HUMAN
Q9UBR2	CATZ_HUMAN
Q9HC84	MUC5B_HUMAN
P16070	CD44_HUMAN
P61916	NPC2_HUMAN
P42785	PCP_HUMAN
P30046	DOPD_HUMAN
P30740	ILEU_HUMAN
Q02818	NUCB1_HUMAN
Q8NFT8	DNER_HUMAN
P62937	PPIA_HUMAN
P00338	LDHA_HUMAN
O95274	LYPD3_HUMAN
Q16378	PROL4_HUMAN
P15289	ARSA_HUMAN
P00738	HPT_HUMAN
P06733	ENOA_HUMAN
P07237	PDIA1_HUMAN
P06576	ATPB_HUMAN
P27482	CALL3_HUMAN
Q9NP55	BPIA1_HUMAN
P15328	FOLR1_HUMAN
Q07955	SRSF1_HUMAN
Q9UKR3	KLK13_HUMAN
P01859	IGHG2_HUMAN
P32926	DSG3_HUMAN
P53634	CATC_HUMAN
P06753	TPM3_HUMAN
P0CG47	UBB_HUMAN
Q9GZZ8	LACRT_HUMAN
P47756	CAPZB_HUMAN
P25311	ZA2G_HUMAN
Q9BPY8	HOP_HUMAN
Q9Y5Z4	HEBP2_HUMAN
O95336	6PGL_HUMAN
O75015	FCG3B_HUMAN
P36952	SPB5_HUMAN
Q96P63	SPB12_HUMAN
P62328	TYB4_HUMAN
P0CF74	IGLC6_HUMAN
O60522	TDRD6_HUMAN
Q16610	ECM1_HUMAN
O60437	PEPL_HUMAN

P04899	GNAI2_HUMAN
P01133	EGF_HUMAN
P00491	PNPH_HUMAN
P55058	PLTP_HUMAN
P06312	KV401_HUMAN
O43852	CALU_HUMAN
A0A0A0MS15	HV349_HUMAN
P40925	MDHC_HUMAN
P18669	PGAM1_HUMAN
P29320	EPHA3_HUMAN
P35579	MYH9_HUMAN
Q96C90	PP14B_HUMAN
P02671	FIBA_HUMAN
Q14118	DAG1_HUMAN
A0A0C4DH38	HV551_HUMAN
Q9UBG3	CRNN_HUMAN
P54108	CRIS3_HUMAN
P35326	SPR2A_HUMAN
A0A075B6H7	KV37_HUMAN
P01009	A1AT_HUMAN
Q96BQ1	FAM3D_HUMAN
Q8NBJ4	GOLM1_HUMAN
P52209	6PGD_HUMAN
P0CG04	IGLC1_HUMAN
O43490	PROM1_HUMAN
Q16881	TRXR1_HUMAN
Q99436	PSB7_HUMAN
P0DMV8	HS71A_HUMAN
Q9BQR3	PRS27_HUMAN
P35030	TRY3_HUMAN
P22532	SPR2D_HUMAN
P23280	CAH6_HUMAN
Q99935	PROL1_HUMAN
P26038	MOES_HUMAN
P02766	TTHY_HUMAN
P54802	ANAG_HUMAN
P22626	ROA2_HUMAN
P51649	SSDH_HUMAN
P09211	GSTP1_HUMAN
P01880	IGHD_HUMAN
P30838	AL3A1_HUMAN
P06702	S10A9_HUMAN
P07900	HS90A_HUMAN
P07384	CAN1_HUMAN
P10599	THIO_HUMAN
P00747	PLMN_HUMAN
P04792	HSPB1_HUMAN
P19021	AMD_HUMAN
P16870	CBPE_HUMAN
Q13510	ASAH1_HUMAN

Q15435	PP1R7_HUMAN
P09668	CATH_HUMAN
P49721	PSB2_HUMAN
Q04446	GLGB_HUMAN
P49913	CAMP_HUMAN
P28799	GRN_HUMAN
P09228	CYTT_HUMAN
P49720	PSB3_HUMAN
P12429	ANXA3_HUMAN
P30041	PRDX6_HUMAN
Q9Y287	ITM2B_HUMAN
P20160	CAP7_HUMAN
P01824	HV439_HUMAN
Q99538	LGMN_HUMAN
P06870	KLK1_HUMAN
Q13421	MSLN_HUMAN
P01860	IGHG3_HUMAN
Q08380	LG3BP_HUMAN
P36955	PEDF_HUMAN
A8K2U0	A2ML1_HUMAN
P01023	A2MG_HUMAN
Q9BX40	LS14B_HUMAN
P60981	DEST_HUMAN
P08174	DAF_HUMAN
P04075	ALDOA_HUMAN
P07711	CATL1_HUMAN
P31151	S10A7_HUMAN
P50395	GDIB_HUMAN
P14314	GLU2B_HUMAN
P52566	GDIR2_HUMAN
P68104	EF1A1_HUMAN
Q15084	PDIA6_HUMAN
P62805	H4_HUMAN
P16930	FAAA_HUMAN
P05109	S10A8_HUMAN
P60174	TPIS_HUMAN
P31944	CASPE_HUMAN
O75083	WDR1_HUMAN
O14950	ML12B_HUMAN
P05089	ARGI1_HUMAN
P37802	TAGL2_HUMAN
P06703	S10A6_HUMAN
P33908	MA1A1_HUMAN
P01772	HV333_HUMAN
Q6XQN6	PNCB_HUMAN
P23141	EST1_HUMAN
O00204	ST2B1_HUMAN
P02787	TRFE_HUMAN
P21128	ENDOU_HUMAN
Q9P1F3	ABRAL_HUMAN

O75976	CBPD_HUMAN
O43278	SPIT1_HUMAN
P01599	KV117_HUMAN
P26641	EF1G_HUMAN
P26885	FKBP2_HUMAN
P13796	PLSL_HUMAN
O60888	CUTA_HUMAN
P20061	TCO1_HUMAN
P01857	IGHG1_HUMAN
Q13423	NNTM_HUMAN
Q6P5S2	LEG1H_HUMAN

Table S3. Proteins identified in RIPA buffer.

UniProt Accession Number	UniProt Entry Name
P01011	AACT_HUMAN
P81605	DCD_HUMAN
Q13835	PKP1_HUMAN
O95968	SG1D1_HUMAN
O75556	SG2A1_HUMAN
Q9HCY8	S10AE_HUMAN
P58107	EPIPL_HUMAN
O75342	LX12B_HUMAN
Q86SG5	S1A7A_HUMAN
Q15517	CDSN_HUMAN

Table S4. Proteins identified on the OsseoSpeed®-like titanium dental implant surface after chemical decontamination with H_2O_2.

UniProt Accession Number	UniProt Entry Name
P02790	HEMO_HUMAN
O75223	GGCT_HUMAN
P00491	PNPH_HUMAN
P37837	TALDO_HUMAN
P55058	PLTP_HUMAN
P06312	KV401_HUMAN
P01871	IGHM_HUMAN
P14923	PLAK_HUMAN
Q06830	PRDX1_HUMAN
P28325	CYTD_HUMAN
Q9UBG3	CRNN_HUMAN
P54108	CRIS3_HUMAN
P04406	G3P_HUMAN
Q6ZVX7	FBX50_HUMAN
Q5T750	KPLCE_HUMAN
P01834	IGKC_HUMAN
P07477	TRY1_HUMAN
P52209	6PGD_HUMAN
P0CG04	IGLC1_HUMAN
P63104	1433Z_HUMAN
Q9UGM3	DMBT1_HUMAN
O43707	ACTN4_HUMAN
Q8N4F0	BPIB2_HUMAN
P01857	IGHG1_HUMAN
P23280	CAH6_HUMAN
P01037	CYTN_HUMAN
P31947	1433S_HUMAN
P01024	CO3_HUMAN
P22735	TGM1_HUMAN
P09211	GSTP1_HUMAN
P01833	PIGR_HUMAN
Q96DR5	BPIA2_HUMAN
P10599	THIO_HUMAN
P06702	S10A9_HUMAN
P11413	G6PD_HUMAN
P80511	S10AC_HUMAN
P11021	BIP_HUMAN
P01877	IGHA2_HUMAN
Q13510	ASAH1_HUMAN
P49721	PSB2_HUMAN
P06744	G6PI_HUMAN
P49913	CAMP_HUMAN
P01036	CYTS_HUMAN
P09228	CYTT_HUMAN
P19961	AMY2B_HUMAN
P30041	PRDX6_HUMAN
P61626	LYSC_HUMAN
Q13867	BLMH_HUMAN
P40926	MDHM_HUMAN

P31025	LCN1_HUMAN
Q08188	TGM3_HUMAN
P07339	CATD_HUMAN
P12273	PIP_HUMAN
Q96DA0	PAUF_HUMAN
A8K2U0	A2ML1_HUMAN
P01040	CYTA_HUMAN
P07355	ANXA2_HUMAN
P22079	PERL_HUMAN
Q01469	FABP5_HUMAN
P31151	S10A7_HUMAN
P68104	EF1A1_HUMAN
P29508	SPB3_HUMAN
Q02413	DSG1_HUMAN
Q5D862	FILA2_HUMAN
P62805	H4_HUMAN
P60709	ACTB_HUMAN
Q9HC84	MUC5B_HUMAN
P05109	S10A8_HUMAN
P04062	GBA1_HUMAN
P30740	ILEU_HUMAN
P31944	CASPE_HUMAN
P01591	IGJ_HUMAN
P01034	CYTC_HUMAN
Q8TDL5	BPIB1_HUMAN
P00338	LDHA_HUMAN
O95274	LYPD3_HUMAN
P59665	DEF1_HUMAN
P08670	VIME_HUMAN
P0DTE7	AMY1B_HUMAN
P68032	ACTC_HUMAN
P31949	S10AB_HUMAN
Q6P5S2	LEG1H_HUMAN
P05089	ARGI1_HUMAN
Q16378	PROL4_HUMAN
P04080	CYTB_HUMAN
P15289	ARSA_HUMAN
P06733	ENOA_HUMAN
P55064	AQP5_HUMAN
P06703	S10A6_HUMAN
P61769	B2MG_HUMAN
Q9NP55	BPIA1_HUMAN
P02763	A1AG1_HUMAN
P01859	IGHG2_HUMAN
P02787	TRFE_HUMAN
Q86YZ3	HORN_HUMAN
P13797	PLST_HUMAN
Q08554	DSC1_HUMAN
P62258	1433E_HUMAN
Q9GZZ8	LACRT_HUMAN

P25311	ZA2G_HUMAN
P02768	ALBU_HUMAN
P68871	HBB_HUMAN
P36952	SPB5_HUMAN
Q96P63	SPB12_HUMAN
P80188	NGAL_HUMAN
P13796	PLSL_HUMAN
P02788	TRFL_HUMAN
P01876	IGHA1_HUMAN
P0CF74	IGLC6_HUMAN
P24158	PRTN3_HUMAN
O14773	TPP1_HUMAN
P15924	DESP_HUMAN
P20061	TCO1_HUMAN
Q96FQ6	S10AG_HUMAN

Table S5. Proteins identified on the OsseoSpeed®-like titanium dental implant surface after chemical decontamination with P407.

UniProt Accession Number	UniProt Entry Name
P0DTE7	AMY1B_HUMAN
P02768	ALBU_HUMAN
P01036	CYTS_HUMAN
Q9HC84	MUC5B_HUMAN
P01876	IGHA1_HUMAN
P12273	PIP_HUMAN
P01037	CYTN_HUMAN
P01833	PIGR_HUMAN
P60709	ACTB_HUMAN
P02788	TRFL_HUMAN
P23280	CAH6_HUMAN
P22079	PERL_HUMAN
P0CG04	IGLC1_HUMAN
P31025	LCN1_HUMAN
P01834	IGKC_HUMAN
P09228	CYTT_HUMAN
Q96DR5	BPIA2_HUMAN
Q6P5S2	LEG1H_HUMAN
P28325	CYTD_HUMAN
P61626	LYSC_HUMAN
P02787	TRFE_HUMAN
P31151	S10A7_HUMAN
Q8N4F0	BPIB2_HUMAN
P25311	ZA2G_HUMAN
P06703	S10A6_HUMAN
O75556	SG2A1_HUMAN
P10599	THIO_HUMAN
P06702	S10A9_HUMAN
Q8TDL5	BPIB1_HUMAN
P01877	IGHA2_HUMAN
P01024	CO3_HUMAN
Q9UGM3	DMBT1_HUMAN
P06733	ENOA_HUMAN
P01857	IGHG1_HUMAN
P01591	IGJ_HUMAN
Q9GZZ8	LACRT_HUMAN
P20061	TCO1_HUMAN
Q96DA0	PAUF_HUMAN
P05109	S10A8_HUMAN
P15924	DESP_HUMAN
P01023	A2MG_HUMAN
P63104	1433Z_HUMAN
P01034	CYTC_HUMAN
Q16378	PROL4_HUMAN
P31947	1433S_HUMAN
P01871	IGHM_HUMAN
P09211	GSTP1_HUMAN
P01859	IGHG2_HUMAN
P13796	PLSL_HUMAN

P61769	B2MG_HUMAN
P00338	LDHA_HUMAN
P55058	PLTP_HUMAN
O95274	LYPD3_HUMAN
P29508	SPB3_HUMAN
P62937	PPIA_HUMAN
Q08188	TGM3_HUMAN
P01009	A1AT_HUMAN
P00450	CERU_HUMAN
P04080	CYTB_HUMAN
P31949	S10AB_HUMAN
P02790	HEMO_HUMAN
Q9UBG3	CRNN_HUMAN
Q01518	CAP1_HUMAN
P30740	ILEU_HUMAN
P07339	CATD_HUMAN
P04406	G3P_HUMAN
P11021	BIP_HUMAN
P68104	EF1A1_HUMAN
Q01469	FABP5_HUMAN
P30041	PRDX6_HUMAN
Q02413	DSG1_HUMAN
Q99935	PROL1_HUMAN
O95968	SG1D1_HUMAN
P01011	AACT_HUMAN
P62258	1433E_HUMAN
P14923	PLAK_HUMAN
P02763	A1AG1_HUMAN
P24158	PRTN3_HUMAN
Q96P63	SPB12_HUMAN
Q9NP55	BPIA1_HUMAN
P80511	S10AC_HUMAN
P81605	DCD_HUMAN
P07355	ANXA2_HUMAN
P35326	SPR2A_HUMAN
P59665	DEF1_HUMAN
P80188	NGAL_HUMAN
P62805	H4_HUMAN
Q13835	PKP1_HUMAN
Q06830	PRDX1_HUMAN
Q96FQ6	S10AG_HUMAN
Q5D862	FILA2_HUMAN
P05089	ARGI1_HUMAN
P68871	HBB_HUMAN
Q13510	ASAH1_HUMAN
P31944	CASPE_HUMAN
Q08554	DSC1_HUMAN
Q6ZVX7	FBX50_HUMAN
P40926	MDHM_HUMAN
P06312	KV401_HUMAN

P00491	PNPH_HUMAN
P47929	LEG7_HUMAN
O00299	CLIC1_HUMAN
Q5T750	KPLCE_HUMAN
P20930	FILA_HUMAN
P68366	TBA4A_HUMAN
Q86YZ3	HORN_HUMAN
Q15517	CDSN_HUMAN
Q14574	DSC3_HUMAN
O75342	LX12B_HUMAN
P01040	CYTA_HUMAN
P20618	PSB1_HUMAN
P58107	EPIPL_HUMAN
Q14956	GPNMB_HUMAN
Q6P4A8	PLBL1_HUMAN

Table S6. Proteins identified on the OsseoSpeed®-like titanium dental implant surface after chemical decontamination with P407 + H_2O_2.

UniProt Accession Number	UniProt Entry Name
P0DTE7	AMY1B_HUMAN
P02768	ALBU_HUMAN
P01036	CYTS_HUMAN
Q9HC84	MUC5B_HUMAN
P01876	IGHA1_HUMAN
P12273	PIP_HUMAN
P01037	CYTN_HUMAN
P01833	PIGR_HUMAN
P60709	ACTB_HUMAN
P02788	TRFL_HUMAN
P23280	CAH6_HUMAN
P22079	PERL_HUMAN
P0CG04	IGLC1_HUMAN
P31025	LCN1_HUMAN
P01834	IGKC_HUMAN
P09228	CYTT_HUMAN
Q96DR5	BPIA2_HUMAN
Q6P5S2	LEG1H_HUMAN
P28325	CYTD_HUMAN
P61626	LYSC_HUMAN
P31151	S10A7_HUMAN
Q8N4F0	BPIB2_HUMAN
P25311	ZA2G_HUMAN
P06703	S10A6_HUMAN
O75556	SG2A1_HUMAN
P10599	THIO_HUMAN
P06702	S10A9_HUMAN
Q8TDL5	BPIB1_HUMAN
P01877	IGHA2_HUMAN
Q9UGM3	DMBT1_HUMAN
P01857	IGHG1_HUMAN
A8K2U0	A2ML1_HUMAN
Q9GZZ8	LACRT_HUMAN
P20061	TCO1_HUMAN
Q96DA0	PAUF_HUMAN
P05109	S10A8_HUMAN
P15924	DESP_HUMAN
P63104	1433Z_HUMAN
P01034	CYTC_HUMAN
Q16378	PROL4_HUMAN
P31947	1433S_HUMAN
P01871	IGHM_HUMAN
P09211	GSTP1_HUMAN
P01859	IGHG2_HUMAN
P13796	PLSL_HUMAN
P61769	B2MG_HUMAN
P00338	LDHA_HUMAN
P55058	PLTP_HUMAN
O95274	LYPD3_HUMAN

P29508	SPB3_HUMAN
P04080	CYTB_HUMAN
P31949	S10AB_HUMAN
P02790	HEMO_HUMAN
Q9UBG3	CRNN_HUMAN
Q01518	CAP1_HUMAN
P30740	ILEU_HUMAN
P07339	CATD_HUMAN
P04406	G3P_HUMAN
P68104	EF1A1_HUMAN
Q01469	FABP5_HUMAN
Q02413	DSG1_HUMAN
P54108	CRIS3_HUMAN
Q99935	PROL1_HUMAN
O95968	SG1D1_HUMAN
P04083	ANXA1_HUMAN
P62258	1433E_HUMAN
P14923	PLAK_HUMAN
P02763	A1AG1_HUMAN
P24158	PRTN3_HUMAN
Q96P63	SPB12_HUMAN
Q9NP55	BPIA1_HUMAN
P81605	DCD_HUMAN
P07355	ANXA2_HUMAN
P13797	PLST_HUMAN
P59665	DEF1_HUMAN
P80188	NGAL_HUMAN
P62805	H4_HUMAN
Q13835	PKP1_HUMAN
Q96FQ6	S10AG_HUMAN
Q5D862	FILA2_HUMAN
P05089	ARGI1_HUMAN
P68871	HBB_HUMAN
Q13510	ASAH1_HUMAN
P31944	CASPE_HUMAN
O75223	GGCT_HUMAN
P49913	CAMP_HUMAN
Q6ZVX7	FBX50_HUMAN
P08571	CD14_HUMAN
P06312	KV401_HUMAN
P15289	ARSA_HUMAN
Q13867	BLMH_HUMAN
O00299	CLIC1_HUMAN
Q5T750	KPLCE_HUMAN
P20930	FILA_HUMAN
Q96QR1	SG3A1_HUMAN
Q15517	CDSN_HUMAN
P07477	TRY1_HUMAN
P04062	GBA1_HUMAN
P22735	TGM1_HUMAN

O75342	LX12B_HUMAN
P01040	CYTA_HUMAN
P20618	PSB1_HUMAN
P58107	EPIPL_HUMAN
Q6P4A8	PLBL1_HUMAN
Q99536	VAT1_HUMAN

Table S7. Proteins identified on the OsseoSpeed®-like titanium dental implant surface after chemical decontamination with NaOCl + AA.

UniProt Accession Number	UniProt Entry Name
P0DTE7	AMY1B_HUMAN
P02768	ALBU_HUMAN
P01036	CYTS_HUMAN
Q9HC84	MUC5B_HUMAN
P01876	IGHA1_HUMAN
P12273	PIP_HUMAN
P01037	CYTN_HUMAN
P01833	PIGR_HUMAN
P60709	ACTB_HUMAN
P02788	TRFL_HUMAN
P23280	CAH6_HUMAN
P22079	PERL_HUMAN
P0CG04	IGLC1_HUMAN
P31025	LCN1_HUMAN
P01834	IGKC_HUMAN
P09228	CYTT_HUMAN
Q96DR5	BPIA2_HUMAN
Q6P5S2	LEG1H_HUMAN
P28325	CYTD_HUMAN
P61626	LYSC_HUMAN
P31151	S10A7_HUMAN
Q8N4F0	BPIB2_HUMAN
P25311	ZA2G_HUMAN
P06703	S10A6_HUMAN
O75556	SG2A1_HUMAN
P10599	THIO_HUMAN
P06702	S10A9_HUMAN
Q8TDL5	BPIB1_HUMAN
P01877	IGHA2_HUMAN
Q9UGM3	DMBT1_HUMAN
P06733	ENOA_HUMAN
P01857	IGHG1_HUMAN
A8K2U0	A2ML1_HUMAN
P01591	IGJ_HUMAN
Q9GZZ8	LACRT_HUMAN
P20061	TCO1_HUMAN
Q96DA0	PAUF_HUMAN
P05109	S10A8_HUMAN
P15924	DESP_HUMAN
P63104	1433Z_HUMAN
P68032	ACTC_HUMAN
Q16378	PROL4_HUMAN
P31947	1433S_HUMAN
P01871	IGHM_HUMAN
P09211	GSTP1_HUMAN
P01859	IGHG2_HUMAN
P13796	PLSL_HUMAN
P61769	B2MG_HUMAN
P00338	LDHA_HUMAN

P55058	PLTP_HUMAN
O95274	LYPD3_HUMAN
P29508	SPB3_HUMAN
Q08188	TGM3_HUMAN
P31949	S10AB_HUMAN
P02790	HEMO_HUMAN
Q9UBG3	CRNN_HUMAN
P07339	CATD_HUMAN
P04406	G3P_HUMAN
P11021	BIP_HUMAN
P68104	EF1A1_HUMAN
Q01469	FABP5_HUMAN
P30041	PRDX6_HUMAN
Q02413	DSG1_HUMAN
Q99935	PROL1_HUMAN
O95968	SG1D1_HUMAN
P13639	EF2_HUMAN
P01011	AACT_HUMAN
P04083	ANXA1_HUMAN
P62258	1433E_HUMAN
P14923	PLAK_HUMAN
P02763	A1AG1_HUMAN
P24158	PRTN3_HUMAN
Q96P63	SPB12_HUMAN
Q9NP55	BPIA1_HUMAN
P81605	DCD_HUMAN
P07355	ANXA2_HUMAN
P35326	SPR2A_HUMAN
P59665	DEF1_HUMAN
O00391	QSOX1_HUMAN
P80188	NGAL_HUMAN
Q13835	PKP1_HUMAN
Q06830	PRDX1_HUMAN
Q96FQ6	S10AG_HUMAN
Q5D862	FILA2_HUMAN
P05089	ARGI1_HUMAN
P68871	HBB_HUMAN
Q13510	ASAH1_HUMAN
P31944	CASPE_HUMAN
Q08554	DSC1_HUMAN
O75223	GGCT_HUMAN
P17931	LEG3_HUMAN
Q6ZVX7	FBX50_HUMAN
O60814	H2B1K_HUMAN
P40926	MDHM_HUMAN
P00491	PNPH_HUMAN
Q13867	BLMH_HUMAN
Q5T750	KPLCE_HUMAN
Q9HCY8	S10AE_HUMAN
O14773	TPP1_HUMAN

P20930	FILA_HUMAN
P68366	TBA4A_HUMAN
Q86YZ3	HORN_HUMAN
Q15517	CDSN_HUMAN
P07477	TRY1_HUMAN
P04062	GBA1_HUMAN
P49721	PSB2_HUMAN
P22735	TGM1_HUMAN
O75342	LX12B_HUMAN
P01040	CYTA_HUMAN
P05120	PAI2_HUMAN
P20618	PSB1_HUMAN
P32119	PRDX2_HUMAN
P35573	GDE_HUMAN
P49720	PSB3_HUMAN
P58107	EPIPL_HUMAN
P60903	S10AA_HUMAN
P63167	DYL1_HUMAN
Q53RT3	APRV1_HUMAN
Q6P4A8	PLBL1_HUMAN
Q8WVV4	POF1B_HUMAN
Q99536	VAT1_HUMAN
Q9BYJ1	LOXE3_HUMAN

Table S8. Proteins identified on the OsseoSpeed®-like titanium dental implant surface after chemical decontamination with H_2O_2 and recontamination with saliva.

UniProt Accession Number	UniProt Entry Name
P0DTE7	AMY1B_HUMAN
P02768	ALBU_HUMAN
P01036	CYTS_HUMAN
Q9HC84	MUC5B_HUMAN
P01876	IGHA1_HUMAN
P12273	PIP_HUMAN
P01037	CYTN_HUMAN
P01833	PIGR_HUMAN
P60709	ACTB_HUMAN
P02788	TRFL_HUMAN
P23280	CAH6_HUMAN
P22079	PERL_HUMAN
P0CG04	IGLC1_HUMAN
P31025	LCN1_HUMAN
P01834	IGKC_HUMAN
P09228	CYTT_HUMAN
Q96DR5	BPIA2_HUMAN
Q6P5S2	LEG1H_HUMAN
P28325	CYTD_HUMAN
P61626	LYSC_HUMAN
P02787	TRFE_HUMAN
P31151	S10A7_HUMAN
Q8N4F0	BPIB2_HUMAN
P40925	MDHC_HUMAN
P25311	ZA2G_HUMAN
P06703	S10A6_HUMAN
O75556	SG2A1_HUMAN
P10599	THIO_HUMAN
P06702	S10A9_HUMAN
Q8TDL5	BPIB1_HUMAN
P01877	IGHA2_HUMAN
P01024	CO3_HUMAN
Q9UGM3	DMBT1_HUMAN
P06733	ENOA_HUMAN
P01857	IGHG1_HUMAN
A8K2U0	A2ML1_HUMAN
P01591	IGJ_HUMAN
Q9GZZ8	LACRT_HUMAN
P02647	APOA1_HUMAN
P20061	TCO1_HUMAN
Q96DA0	PAUF_HUMAN
P05109	S10A8_HUMAN
P15924	DESP_HUMAN
P01023	A2MG_HUMAN
P63104	1433Z_HUMAN
P01034	CYTC_HUMAN
O43707	ACTN4_HUMAN
P68032	ACTC_HUMAN
Q16378	PROL4_HUMAN

P31947	1433S_HUMAN
Q9Y6R7	FCGBP_HUMAN
P0CF74	IGLC6_HUMAN
P80303	NUCB2_HUMAN
P52209	6PGD_HUMAN
P01871	IGHM_HUMAN
P09211	GSTP1_HUMAN
P01859	IGHG2_HUMAN
P13796	PLSL_HUMAN
P61769	B2MG_HUMAN
P06396	GELS_HUMAN
P00338	LDHA_HUMAN
P55058	PLTP_HUMAN
P06744	G6PI_HUMAN
P60174	TPIS_HUMAN
O95274	LYPD3_HUMAN
P29401	TKT_HUMAN
P29508	SPB3_HUMAN
P07237	PDIA1_HUMAN
P62937	PPIA_HUMAN
Q02487	DSC2_HUMAN
P00738	HPT_HUMAN
Q08188	TGM3_HUMAN
P01861	IGHG4_HUMAN
P00450	CERU_HUMAN
P04080	CYTB_HUMAN
P31949	S10AB_HUMAN
P04066	FUCO_HUMAN
Q96HE7	ERO1A_HUMAN
P02790	HEMO_HUMAN
O00584	RNT2_HUMAN
P09960	LKHA4_HUMAN
Q9UBG3	CRNN_HUMAN
P27482	CALL3_HUMAN
Q6S8J3	POTEE_HUMAN
P07602	SAP_HUMAN
P02774	VTDB_HUMAN
Q01518	CAP1_HUMAN
P30740	ILEU_HUMAN
P04075	ALDOA_HUMAN
Q969S9	RRF2M_HUMAN
P07339	CATD_HUMAN
P04406	G3P_HUMAN
P11021	BIP_HUMAN
P68104	EF1A1_HUMAN
A0A075B6H7	KV37_HUMAN
P25815	S100P_HUMAN
Q08380	LG3BP_HUMAN
Q01469	FABP5_HUMAN
P19021	AMD_HUMAN

P30041	PRDX6_HUMAN
Q02413	DSG1_HUMAN
O60437	PEPL_HUMAN
P54108	CRIS3_HUMAN
P37802	TAGL2_HUMAN
P05164	PERM_HUMAN
P0DMV8	HS71A_HUMAN
Q99935	PROL1_HUMAN
O95968	SG1D1_HUMAN
P07108	ACBP_HUMAN
P50395	GDIB_HUMAN
P13639	EF2_HUMAN
P01033	TIMP1_HUMAN
P32926	DSG3_HUMAN
P01011	AACT_HUMAN
P27797	CALR_HUMAN
P02675	FIBB_HUMAN
P04083	ANXA1_HUMAN
P11142	HSP7C_HUMAN
P04217	A1BG_HUMAN
P62258	1433E_HUMAN
P15311	EZRI_HUMAN
P0C0L4	CO4A_HUMAN
P01860	IGHG3_HUMAN
Q15782	CH3L2_HUMAN
P36952	SPB5_HUMAN
P14923	PLAK_HUMAN
P02763	A1AG1_HUMAN
Q14515	SPRL1_HUMAN
P35321	SPR1A_HUMAN
P48729	KC1A_HUMAN
O60218	AK1BA_HUMAN
P33908	MA1A1_HUMAN
P07900	HS90A_HUMAN
P14780	MMP9_HUMAN
P06870	KLK1_HUMAN
Q9UBC9	SPRR3_HUMAN
P37837	TALDO_HUMAN
P12814	ACTN1_HUMAN
P04040	CATA_HUMAN
Q07654	TFF3_HUMAN
P14618	KPYM_HUMAN
P31946	1433B_HUMAN
P24158	PRTN3_HUMAN
P18669	PGAM1_HUMAN
Q9NZT1	CALL5_HUMAN
P05067	A4_HUMAN
P30086	PEBP1_HUMAN
Q96P63	SPB12_HUMAN
Q9NP55	BPIA1_HUMAN

Q9NQ38	ISK5_HUMAN
P80511	S10AC_HUMAN
P18510	IL1RA_HUMAN
P02765	FETUA_HUMAN
P21128	ENDOU_HUMAN
P81605	DCD_HUMAN
P07355	ANXA2_HUMAN
P17213	BPI_HUMAN
P25705	ATPA_HUMAN
P02750	A2GL_HUMAN
P35326	SPR2A_HUMAN
P13797	PLST_HUMAN
P01619	KV320_HUMAN
P02679	FIBG_HUMAN
P35579	MYH9_HUMAN
Q13421	MSLN_HUMAN
P53634	CATC_HUMAN
Q13838	DX39B_HUMAN
P06737	PYGL_HUMAN
P59665	DEF1_HUMAN
P30838	AL3A1_HUMAN
O00391	QSOX1_HUMAN
P80188	NGAL_HUMAN
P61916	NPC2_HUMAN
Q14508	WFDC2_HUMAN
P62805	H4_HUMAN
P17174	AATC_HUMAN
Q9UIV8	SPB13_HUMAN
P07858	CATB_HUMAN
P03973	SLPI_HUMAN
P00558	PGK1_HUMAN
P07384	CAN1_HUMAN
O00462	MANBA_HUMAN
Q16610	ECM1_HUMAN
P0DP23	CALM1_HUMAN
P18206	VINC_HUMAN
Q14914	PTGR1_HUMAN
P16870	CBPE_HUMAN
P22392	NDKB_HUMAN
Q07812	BAX_HUMAN
Q06323	PSME1_HUMAN
P08246	ELNE_HUMAN
Q06830	PRDX1_HUMAN
Q96FQ6	S10AG_HUMAN
Q9Y5Z4	HEBP2_HUMAN
P12429	ANXA3_HUMAN
P40121	CAPG_HUMAN
Q5D862	FILA2_HUMAN
Q05639	EF1A2_HUMAN
Q9ULZ3	ASC_HUMAN

Q9UKG9	OCTC_HUMAN
P01782	HV309_HUMAN
P00441	SODC_HUMAN
Q96RM1	SPR2F_HUMAN
P05089	ARGI1_HUMAN
P68871	HBB_HUMAN
Q13510	ASAH1_HUMAN
P29320	EPHA3_HUMAN
P46940	IQGA1_HUMAN
P43490	NAMPT_HUMAN
O00748	EST2_HUMAN
P07711	CATL1_HUMAN
Q07955	SRSF1_HUMAN
Q9P1F3	ABRAL_HUMAN
P10909	CLUS_HUMAN
P31944	CASPE_HUMAN
P48163	MAOX_HUMAN
P31146	COR1A_HUMAN
P07195	LDHB_HUMAN
P0CG47	UBB_HUMAN
P01764	HV323_HUMAN
P00751	CFAB_HUMAN
Q14624	ITIH4_HUMAN
Q96BQ1	FAM3D_HUMAN
P30044	PRDX5_HUMAN
Q9Y376	CAB39_HUMAN
Q16696	CP2AD_HUMAN
P52566	GDIR2_HUMAN
P31150	GDIA_HUMAN
Q08554	DSC1_HUMAN
Q9UKR3	KLK13_HUMAN
P62328	TYB4_HUMAN
O75223	GGCT_HUMAN
P21926	CD9_HUMAN
P49913	CAMP_HUMAN
P47756	CAPZB_HUMAN
P07737	PROF1_HUMAN
P01772	HV333_HUMAN
Q02818	NUCB1_HUMAN
Q15435	PP1R7_HUMAN
P19961	AMY2B_HUMAN
O75368	SH3L1_HUMAN
P55000	SLUR1_HUMAN
P23141	EST1_HUMAN
P15328	FOLR1_HUMAN
P62136	PP1A_HUMAN
Q9UHL4	DPP2_HUMAN
P63261	ACTG_HUMAN
Q9Y4L1	HYOU1_HUMAN
P17900	SAP3_HUMAN

P06865	HEXA_HUMAN
P80748	LV321_HUMAN
A0A0C4DH38	HV551_HUMAN
P28799	GRN_HUMAN
P17931	LEG3_HUMAN
Q9UBX7	KLK11_HUMAN
O43490	PROM1_HUMAN
Q6ZVX7	FBX50_HUMAN
P01133	EGF_HUMAN
P08571	CD14_HUMAN
P61088	UBE2N_HUMAN
P52565	GDIR1_HUMAN
Q96G03	PGM2_HUMAN
Q9BPY8	HOP_HUMAN
Q15080	NCF4_HUMAN
P40926	MDHM_HUMAN
P22894	MMP8_HUMAN
P02766	TTHY_HUMAN
P29034	S10A2_HUMAN
O75449	KTNA1_HUMAN
P99999	CYC_HUMAN
P62942	FKB1A_HUMAN
Q12841	FSTL1_HUMAN
P26038	MOES_HUMAN
P13987	CD59_HUMAN
P19823	ITIH2_HUMAN
P17655	CAN2_HUMAN
Q8NFT8	DNER_HUMAN
A0A0C4DH36	HV338_HUMAN
P11413	G6PD_HUMAN
Q99538	LGMN_HUMAN
P06312	KV401_HUMAN
P15289	ARSA_HUMAN
Q9BX40	LS14B_HUMAN
P48594	SPB4_HUMAN
Q6XQN6	PNCB_HUMAN
P09668	CATH_HUMAN
P00491	PNPH_HUMAN
O60235	TM11D_HUMAN
Q13867	BLMH_HUMAN
Q8N474	SFRP1_HUMAN
P22532	SPR2D_HUMAN
P42785	PCP_HUMAN
Q92841	DDX17_HUMAN
P12830	CADH1_HUMAN
P47929	LEG7_HUMAN
O43852	CALU_HUMAN
Q9Y697	NFS1_HUMAN
Q6UX06	OLFM4_HUMAN
Q9UKR0	KLK12_HUMAN

P01700	LV147_HUMAN
Q9H299	SH3L3_HUMAN
P26641	EF1G_HUMAN
P11684	UTER_HUMAN
P61586	RHOA_HUMAN
P00747	PLMN_HUMAN
Q13423	NNTM_HUMAN
P23284	PPIB_HUMAN
Q9NY33	DPP3_HUMAN
P08311	CATG_HUMAN
P51649	SSDH_HUMAN
Q9UBR2	CATZ_HUMAN
Q92520	FAM3C_HUMAN
P09958	FURIN_HUMAN
O14950	ML12B_HUMAN
P22314	UBA1_HUMAN
Q15084	PDIA6_HUMAN
Q05707	COEA1_HUMAN
P06576	ATPB_HUMAN
O60293	ZC3H1_HUMAN
P01780	HV307_HUMAN
P84077	ARF1_HUMAN
O60888	CUTA_HUMAN
P36955	PEDF_HUMAN
P30046	DOPD_HUMAN
Q8WWA0	ITLN1_HUMAN
A0A075B6R9	KVD24_HUMAN
O75071	EFC14_HUMAN
P53597	SUCA_HUMAN
Q9UL46	PSME2_HUMAN
P61158	ARP3_HUMAN
A0A0C4DH35	HV335_HUMAN
Q5SSG8	MUC21_HUMAN
P23083	HV102_HUMAN
O75976	CBPD_HUMAN
Q5T750	KPLCE_HUMAN
P60981	DEST_HUMAN
P01817	HV205_HUMAN
Q92876	KLK6_HUMAN
P01743	HV146_HUMAN
Q9HCY8	S10AE_HUMAN
P69905	HBA_HUMAN
O14773	TPP1_HUMAN
P22626	ROA2_HUMAN
P02671	FIBA_HUMAN
O75083	WDR1_HUMAN
Q96QR1	SG3A1_HUMAN
O75629	CREG1_HUMAN
O76031	CLPX_HUMAN
P25787	PSA2_HUMAN

P60660	MYL6_HUMAN
Q96JB3	HIC2_HUMAN
Q9Y287	ITM2B_HUMAN
P40189	IL6RB_HUMAN
P01880	IGHD_HUMAN
Q9UKQ9	KLK9_HUMAN
Q9GZM7	TINAL_HUMAN
P26447	S10A4_HUMAN
P07477	TRY1_HUMAN
P04899	GNAI2_HUMAN
P05090	APOD_HUMAN
P00492	HPRT_HUMAN
P08670	VIME_HUMAN
P01721	LV657_HUMAN
O75594	PGRP1_HUMAN
P30101	PDIA3_HUMAN
P68036	UB2L3_HUMAN
Q14574	DSC3_HUMAN
A0A0A0MS15	HV349_HUMAN
O00204	ST2B1_HUMAN
Q14118	DAG1_HUMAN
P35052	GPC1_HUMAN
Q9BXJ4	C1QT3_HUMAN
Q8NBJ4	GOLM1_HUMAN
Q01082	SPTB2_HUMAN
P26885	FKBP2_HUMAN
Q14697	GANAB_HUMAN
Q9BQR3	PRS27_HUMAN
Q96S96	PEBP4_HUMAN
P01599	KV117_HUMAN
Q15843	NEDD8_HUMAN
P33176	KINH_HUMAN
O95479	G6PE_HUMAN
P15309	PPAP_HUMAN
P35030	TRY3_HUMAN
P59998	ARPC4_HUMAN
P04062	GBA1_HUMAN
O75874	IDHC_HUMAN
P49721	PSB2_HUMAN
P22735	TGM1_HUMAN
P16930	FAAA_HUMAN

Table S9. Proteins identified on the OsseoSpeed®-like titanium dental implant surface after chemical decontamination with P407 and recontamination with saliva.

UniProt Accession Number	UniProt Entry Name
P0DTE7	AMY1B_HUMAN
P02768	ALBU_HUMAN
P01036	CYTS_HUMAN
Q9HC84	MUC5B_HUMAN
P01876	IGHA1_HUMAN
P12273	PIP_HUMAN
P01037	CYTN_HUMAN
P01833	PIGR_HUMAN
P60709	ACTB_HUMAN
P02788	TRFL_HUMAN
P23280	CAH6_HUMAN
P22079	PERL_HUMAN
P0CG04	IGLC1_HUMAN
P31025	LCN1_HUMAN
P01834	IGKC_HUMAN
P09228	CYTT_HUMAN
Q96DR5	BPIA2_HUMAN
Q6P5S2	LEG1H_HUMAN
P28325	CYTD_HUMAN
P61626	LYSC_HUMAN
P02787	TRFE_HUMAN
P31151	S10A7_HUMAN
Q8N4F0	BPIB2_HUMAN
P40925	MDHC_HUMAN
P25311	ZA2G_HUMAN
P06703	S10A6_HUMAN
O75556	SG2A1_HUMAN
P10599	THIO_HUMAN
P06702	S10A9_HUMAN
Q8TDL5	BPIB1_HUMAN
P01877	IGHA2_HUMAN
P01024	CO3_HUMAN
Q9UGM3	DMBT1_HUMAN
P06733	ENOA_HUMAN
P01857	IGHG1_HUMAN
A8K2U0	A2ML1_HUMAN
P01591	IGJ_HUMAN
Q9GZZ8	LACRT_HUMAN
P02647	APOA1_HUMAN
P20061	TCO1_HUMAN
Q96DA0	PAUF_HUMAN
P05109	S10A8_HUMAN
P15924	DESP_HUMAN
P01023	A2MG_HUMAN
P63104	1433Z_HUMAN
P01034	CYTC_HUMAN
O43707	ACTN4_HUMAN
P68032	ACTC_HUMAN
Q16378	PROL4_HUMAN

P31947	1433S_HUMAN
Q9Y6R7	FCGBP_HUMAN
P0CF74	IGLC6_HUMAN
P80303	NUCB2_HUMAN
P52209	6PGD_HUMAN
P01871	IGHM_HUMAN
P09211	GSTP1_HUMAN
P01859	IGHG2_HUMAN
P13796	PLSL_HUMAN
P61769	B2MG_HUMAN
P06396	GELS_HUMAN
P00338	LDHA_HUMAN
P55058	PLTP_HUMAN
P06744	G6PI_HUMAN
P60174	TPIS_HUMAN
O95274	LYPD3_HUMAN
P29401	TKT_HUMAN
P29508	SPB3_HUMAN
P07237	PDIA1_HUMAN
P62937	PPIA_HUMAN
Q02487	DSC2_HUMAN
P00738	HPT_HUMAN
Q08188	TGM3_HUMAN
P01861	IGHG4_HUMAN
P00450	CERU_HUMAN
P04080	CYTB_HUMAN
P31949	S10AB_HUMAN
P04066	FUCO_HUMAN
Q96HE7	ERO1A_HUMAN
P02790	HEMO_HUMAN
O00584	RNT2_HUMAN
P09960	LKHA4_HUMAN
Q9UBG3	CRNN_HUMAN
P27482	CALL3_HUMAN
Q6S8J3	POTEE_HUMAN
P07602	SAP_HUMAN
P02774	VTDB_HUMAN
Q01518	CAP1_HUMAN
P30740	ILEU_HUMAN
P04075	ALDOA_HUMAN
Q969S9	RRF2M_HUMAN
P07339	CATD_HUMAN
P04406	G3P_HUMAN
P11021	BIP_HUMAN
P68104	EF1A1_HUMAN
A0A075B6H7	KV37_HUMAN
P25815	S100P_HUMAN
Q08380	LG3BP_HUMAN
Q01469	FABP5_HUMAN
P19021	AMD_HUMAN

P30041	PRDX6_HUMAN
Q02413	DSG1_HUMAN
O60437	PEPL_HUMAN
P54108	CRIS3_HUMAN
P37802	TAGL2_HUMAN
P05164	PERM_HUMAN
P0DMV8	HS71A_HUMAN
Q99935	PROL1_HUMAN
O95968	SG1D1_HUMAN
P07108	ACBP_HUMAN
P50395	GDIB_HUMAN
P13639	EF2_HUMAN
P01033	TIMP1_HUMAN
P32926	DSG3_HUMAN
P27797	CALR_HUMAN
P02675	FIBB_HUMAN
P04083	ANXA1_HUMAN
P11142	HSP7C_HUMAN
P04217	A1BG_HUMAN
P62258	1433E_HUMAN
P15311	EZRI_HUMAN
P0C0L4	CO4A_HUMAN
P01860	IGHG3_HUMAN
Q15782	CH3L2_HUMAN
P36952	SPB5_HUMAN
P14923	PLAK_HUMAN
P02763	A1AG1_HUMAN
Q14515	SPRL1_HUMAN
P35321	SPR1A_HUMAN
P48729	KC1A_HUMAN
O60218	AK1BA_HUMAN
P33908	MA1A1_HUMAN
P07900	HS90A_HUMAN
Q13576	IQGA2_HUMAN
P14780	MMP9_HUMAN
P06870	KLK1_HUMAN
Q9UBC9	SPRR3_HUMAN
P37837	TALDO_HUMAN
P12814	ACTN1_HUMAN
P04040	CATA_HUMAN
Q07654	TFF3_HUMAN
Q562R1	ACTBL_HUMAN
P14618	KPYM_HUMAN
P31946	1433B_HUMAN
P24158	PRTN3_HUMAN
P18669	PGAM1_HUMAN
Q9NZT1	CALL5_HUMAN
P05067	A4_HUMAN
P30086	PEBP1_HUMAN
Q96P63	SPB12_HUMAN

Q9NP55	BPIA1_HUMAN
Q9NQ38	ISK5_HUMAN
P80511	S10AC_HUMAN
P18510	IL1RA_HUMAN
P02765	FETUA_HUMAN
P21128	ENDOU_HUMAN
P81605	DCD_HUMAN
P07355	ANXA2_HUMAN
P17213	BPI_HUMAN
P25705	ATPA_HUMAN
P02750	A2GL_HUMAN
P35326	SPR2A_HUMAN
P13797	PLST_HUMAN
P01619	KV320_HUMAN
P02679	FIBG_HUMAN
P35579	MYH9_HUMAN
Q13421	MSLN_HUMAN
P53634	CATC_HUMAN
P06737	PYGL_HUMAN
P59665	DEF1_HUMAN
P30838	AL3A1_HUMAN
O00391	QSOX1_HUMAN
P80188	NGAL_HUMAN
P61916	NPC2_HUMAN
Q14508	WFDC2_HUMAN
P62805	H4_HUMAN
P17174	AATC_HUMAN
Q9UIV8	SPB13_HUMAN
P07858	CATB_HUMAN
P03973	SLPI_HUMAN
P00558	PGK1_HUMAN
P07384	CAN1_HUMAN
O00462	MANBA_HUMAN
Q16610	ECM1_HUMAN
P0DP23	CALM1_HUMAN
Q13835	PKP1_HUMAN
P18206	VINC_HUMAN
P02749	APOH_HUMAN
Q14914	PTGR1_HUMAN
P16870	CBPE_HUMAN
P22392	NDKB_HUMAN
Q07812	BAX_HUMAN
Q06323	PSME1_HUMAN
P08246	ELNE_HUMAN
Q06830	PRDX1_HUMAN
Q96FQ6	S10AG_HUMAN
Q9Y5Z4	HEBP2_HUMAN
P12429	ANXA3_HUMAN
P40121	CAPG_HUMAN
Q5D862	FILA2_HUMAN

O00764	PDXK_HUMAN
Q9ULZ3	ASC_HUMAN
Q9UKG9	OCTC_HUMAN
P01782	HV309_HUMAN
P00441	SODC_HUMAN
P01008	ANT3_HUMAN
Q96RM1	SPR2F_HUMAN
P05089	ARGI1_HUMAN
P68871	HBB_HUMAN
Q13510	ASAH1_HUMAN
P29320	EPHA3_HUMAN
P46940	IQGA1_HUMAN
P43490	NAMPT_HUMAN
O00748	EST2_HUMAN
P07711	CATL1_HUMAN
Q07955	SRSF1_HUMAN
Q9P1F3	ABRAL_HUMAN
P10909	CLUS_HUMAN
P31944	CASPE_HUMAN
P48163	MAOX_HUMAN
P31146	COR1A_HUMAN
P07195	LDHB_HUMAN
P0CG47	UBB_HUMAN
P01764	HV323_HUMAN
P00751	CFAB_HUMAN
Q14624	ITIH4_HUMAN
Q96BQ1	FAM3D_HUMAN
P30044	PRDX5_HUMAN
Q9Y376	CAB39_HUMAN
Q16696	CP2AD_HUMAN
P52566	GDIR2_HUMAN
P31150	GDIA_HUMAN
Q08554	DSC1_HUMAN
Q9UKR3	KLK13_HUMAN
P62328	TYB4_HUMAN
O75223	GGCT_HUMAN
P21926	CD9_HUMAN
P49913	CAMP_HUMAN
P47756	CAPZB_HUMAN
P07737	PROF1_HUMAN
P01772	HV333_HUMAN
Q02818	NUCB1_HUMAN
Q15435	PP1R7_HUMAN
P19961	AMY2B_HUMAN
O75368	SH3L1_HUMAN
P55000	SLUR1_HUMAN
P23141	EST1_HUMAN
P15328	FOLR1_HUMAN
P62136	PP1A_HUMAN
Q9UHL4	DPP2_HUMAN

P63261	ACTG_HUMAN
Q9Y4L1	HYOU1_HUMAN
P17900	SAP3_HUMAN
P06865	HEXA_HUMAN
P80748	LV321_HUMAN
A0A0C4DH38	HV551_HUMAN
P28799	GRN_HUMAN
Q9UBX7	KLK11_HUMAN
O43490	PROM1_HUMAN
Q6ZVX7	FBX50_HUMAN
P01133	EGF_HUMAN
P08571	CD14_HUMAN
O60814	H2B1K_HUMAN
P61088	UBE2N_HUMAN
P52565	GDIR1_HUMAN
Q96G03	PGM2_HUMAN
P19827	ITIH1_HUMAN
Q9BPY8	HOP_HUMAN
Q15080	NCF4_HUMAN
P40926	MDHM_HUMAN
P22894	MMP8_HUMAN
P02766	TTHY_HUMAN
P06753	TPM3_HUMAN
P29034	S10A2_HUMAN
O75449	KTNA1_HUMAN
P99999	CYC_HUMAN
P62942	FKB1A_HUMAN
Q12841	FSTL1_HUMAN
P26038	MOES_HUMAN
P13987	CD59_HUMAN
P17655	CAN2_HUMAN
Q8NFT8	DNER_HUMAN
A0A0C4DH36	HV338_HUMAN
P11413	G6PD_HUMAN
Q99538	LGMN_HUMAN
P06312	KV401_HUMAN
P15289	ARSA_HUMAN
P48594	SPB4_HUMAN
Q6XQN6	PNCB_HUMAN
Q9UBH0	I36RA_HUMAN
P09668	CATH_HUMAN
P00491	PNPH_HUMAN
O60235	TM11D_HUMAN
P04908	H2A1B_HUMAN
Q13867	BLMH_HUMAN
Q8N474	SFRP1_HUMAN
P22532	SPR2D_HUMAN
P42785	PCP_HUMAN
Q92841	DDX17_HUMAN
P47929	LEG7_HUMAN

O43852	CALU_HUMAN
Q9Y697	NFS1_HUMAN
Q6UX06	OLFM4_HUMAN
Q9UKR0	KLK12_HUMAN
P01700	LV147_HUMAN
Q9H299	SH3L3_HUMAN
P26641	EF1G_HUMAN
P61586	RHOA_HUMAN
P00747	PLMN_HUMAN
Q13423	NNTM_HUMAN
P23284	PPIB_HUMAN
Q9NY33	DPP3_HUMAN
P55786	PSA_HUMAN
P08311	CATG_HUMAN
P51649	SSDH_HUMAN
Q9UBR2	CATZ_HUMAN
P14314	GLU2B_HUMAN
P09958	FURIN_HUMAN
O14950	ML12B_HUMAN
Q15084	PDIA6_HUMAN
P14174	MIF_HUMAN
P02808	STAT_HUMAN
O60293	ZC3H1_HUMAN
P01780	HV307_HUMAN
P84077	ARF1_HUMAN
O60888	CUTA_HUMAN
P36955	PEDF_HUMAN
P30046	DOPD_HUMAN
Q8WWA0	ITLN1_HUMAN
A0A075B6R9	KVD24_HUMAN
O75071	EFC14_HUMAN
P53597	SUCA_HUMAN
Q9UL46	PSME2_HUMAN
P61158	ARP3_HUMAN
A0A0C4DH35	HV335_HUMAN
Q5SSG8	MUC21_HUMAN
P23083	HV102_HUMAN
O75976	CBPD_HUMAN
P60981	DEST_HUMAN
P01817	HV205_HUMAN
Q92876	KLK6_HUMAN
P01743	HV146_HUMAN
Q9HCY8	S10AE_HUMAN
P69905	HBA_HUMAN
O14773	TPP1_HUMAN
O75015	FCG3B_HUMAN
P02671	FIBA_HUMAN
O75083	WDR1_HUMAN
Q96QR1	SG3A1_HUMAN
O75629	CREG1_HUMAN

O76031	CLPX_HUMAN
P25929	NPY1R_HUMAN
P12109	CO6A1_HUMAN
P25787	PSA2_HUMAN
P60660	MYL6_HUMAN
A0A0B4J1V0	HV315_HUMAN
P29373	RABP2_HUMAN
Q9Y287	ITM2B_HUMAN
Q86YZ3	HORN_HUMAN
P15153	RAC2_HUMAN
P40189	IL6RB_HUMAN
P01880	IGHD_HUMAN
Q9UKQ9	KLK9_HUMAN
Q9GZM7	TINAL_HUMAN
P07477	TRY1_HUMAN
P04899	GNAI2_HUMAN
P05090	APOD_HUMAN
P00492	HPRT_HUMAN
P01721	LV657_HUMAN
Q14574	DSC3_HUMAN
A0A0A0MS15	HV349_HUMAN
O00204	ST2B1_HUMAN
Q9BXJ4	C1QT3_HUMAN
Q8NBJ4	GOLM1_HUMAN
P26885	FKBP2_HUMAN
Q14697	GANAB_HUMAN
Q9BQR3	PRS27_HUMAN
P19652	A1AG2_HUMAN
P01599	KV117_HUMAN
Q15843	NEDD8_HUMAN
O75874	IDHC_HUMAN
P49721	PSB2_HUMAN
O43592	XPOT_HUMAN
O60522	TDRD6_HUMAN
O75347	TBCA_HUMAN
P01042	KNG1_HUMAN
P01824	HV439_HUMAN
P04179	SODM_HUMAN
P04433	KV311_HUMAN
P04792	HSPB1_HUMAN
P05156	CFAI_HUMAN
P08174	DAF_HUMAN
P15531	NDKA_HUMAN
P16070	CD44_HUMAN
P36871	PGM1_HUMAN
P40394	ADH7_HUMAN
P49720	PSB3_HUMAN
Q14CN2	CLCA4_HUMAN
Q15691	MARE1_HUMAN
Q6NUJ1	SAPL1_HUMAN

Q9BWS9	CHID1_HUMAN
Q9NZD2	GLTP_HUMAN
Q9UJ70	NAGK_HUMAN

Table S10. Proteins identified on the OsseoSpeed®-like titanium dental implant surface after chemical decontamination with P407 + H_2O_2 and recontamination with saliva.

UniProt Accession Number	UniProt Entry Name
P0DTE7	AMY1B_HUMAN
P02768	ALBU_HUMAN
P01036	CYTS_HUMAN
Q9HC84	MUC5B_HUMAN
P01876	IGHA1_HUMAN
P12273	PIP_HUMAN
P01037	CYTN_HUMAN
P01833	PIGR_HUMAN
P60709	ACTB_HUMAN
P02788	TRFL_HUMAN
P23280	CAH6_HUMAN
P22079	PERL_HUMAN
P0CG04	IGLC1_HUMAN
P31025	LCN1_HUMAN
P01834	IGKC_HUMAN
P09228	CYTT_HUMAN
Q96DR5	BPIA2_HUMAN
Q6P5S2	LEG1H_HUMAN
P28325	CYTD_HUMAN
P61626	LYSC_HUMAN
P02787	TRFE_HUMAN
P31151	S10A7_HUMAN
Q8N4F0	BPIB2_HUMAN
P40925	MDHC_HUMAN
P25311	ZA2G_HUMAN
P06703	S10A6_HUMAN
O75556	SG2A1_HUMAN
P10599	THIO_HUMAN
P06702	S10A9_HUMAN
Q8TDL5	BPIB1_HUMAN
P01877	IGHA2_HUMAN
P01024	CO3_HUMAN
Q9UGM3	DMBT1_HUMAN
P06733	ENOA_HUMAN
P01857	IGHG1_HUMAN
A8K2U0	A2ML1_HUMAN
P01591	IGJ_HUMAN
Q9GZZ8	LACRT_HUMAN
P02647	APOA1_HUMAN
P20061	TCO1_HUMAN
Q96DA0	PAUF_HUMAN
P05109	S10A8_HUMAN
P15924	DESP_HUMAN
P01023	A2MG_HUMAN
P63104	1433Z_HUMAN
P01034	CYTC_HUMAN
O43707	ACTN4_HUMAN
P68032	ACTC_HUMAN
Q16378	PROL4_HUMAN

P31947	1433S_HUMAN
Q9Y6R7	FCGBP_HUMAN
P0CF74	IGLC6_HUMAN
P80303	NUCB2_HUMAN
P52209	6PGD_HUMAN
P01871	IGHM_HUMAN
P09211	GSTP1_HUMAN
Q92673	SORL_HUMAN
P01859	IGHG2_HUMAN
P13796	PLSL_HUMAN
P61769	B2MG_HUMAN
P06396	GELS_HUMAN
P00338	LDHA_HUMAN
P55058	PLTP_HUMAN
P06744	G6PI_HUMAN
P60174	TPIS_HUMAN
O95274	LYPD3_HUMAN
P29401	TKT_HUMAN
P29508	SPB3_HUMAN
P07237	PDIA1_HUMAN
P62937	PPIA_HUMAN
Q02487	DSC2_HUMAN
P00738	HPT_HUMAN
Q08188	TGM3_HUMAN
P01861	IGHG4_HUMAN
P00450	CERU_HUMAN
P04080	CYTB_HUMAN
P31949	S10AB_HUMAN
Q96HE7	ERO1A_HUMAN
P02790	HEMO_HUMAN
O00584	RNT2_HUMAN
P09960	LKHA4_HUMAN
Q9UBG3	CRNN_HUMAN
P27482	CALL3_HUMAN
Q6S8J3	POTEE_HUMAN
P07602	SAP_HUMAN
Q01518	CAP1_HUMAN
P30740	ILEU_HUMAN
P04075	ALDOA_HUMAN
Q969S9	RRF2M_HUMAN
P07339	CATD_HUMAN
P04406	G3P_HUMAN
P11021	BIP_HUMAN
P68104	EF1A1_HUMAN
P25815	S100P_HUMAN
Q08380	LG3BP_HUMAN
Q01469	FABP5_HUMAN
P60842	IF4A1_HUMAN
P19021	AMD_HUMAN
P30041	PRDX6_HUMAN

Q02413	DSG1_HUMAN
O60437	PEPL_HUMAN
P54108	CRIS3_HUMAN
P37802	TAGL2_HUMAN
P05164	PERM_HUMAN
P0DMV8	HS71A_HUMAN
Q99935	PROL1_HUMAN
O95968	SG1D1_HUMAN
P07108	ACBP_HUMAN
P50395	GDIB_HUMAN
P13639	EF2_HUMAN
P01033	TIMP1_HUMAN
P32926	DSG3_HUMAN
P01011	AACT_HUMAN
P27797	CALR_HUMAN
P02675	FIBB_HUMAN
P04083	ANXA1_HUMAN
P11142	HSP7C_HUMAN
P62258	1433E_HUMAN
P15311	EZRI_HUMAN
P0C0L4	CO4A_HUMAN
P01860	IGHG3_HUMAN
Q15782	CH3L2_HUMAN
P36952	SPB5_HUMAN
P14923	PLAK_HUMAN
P02763	A1AG1_HUMAN
P35321	SPR1A_HUMAN
P48729	KC1A_HUMAN
O60218	AK1BA_HUMAN
P33908	MA1A1_HUMAN
P07900	HS90A_HUMAN
Q13576	IQGA2_HUMAN
P14780	MMP9_HUMAN
P06870	KLK1_HUMAN
Q9UBC9	SPRR3_HUMAN
P37837	TALDO_HUMAN
P12814	ACTN1_HUMAN
P04040	CATA_HUMAN
Q07654	TFF3_HUMAN
P14618	KPYM_HUMAN
P31946	1433B_HUMAN
P24158	PRTN3_HUMAN
P18669	PGAM1_HUMAN
Q9NZT1	CALL5_HUMAN
P30086	PEBP1_HUMAN
Q96P63	SPB12_HUMAN
Q9NP55	BPIA1_HUMAN
Q9NQ38	ISK5_HUMAN
P80511	S10AC_HUMAN
P18510	IL1RA_HUMAN

P02765	FETUA_HUMAN
P21128	ENDOU_HUMAN
P81605	DCD_HUMAN
P07355	ANXA2_HUMAN
P17213	BPI_HUMAN
P25705	ATPA_HUMAN
P35326	SPR2A_HUMAN
P13797	PLST_HUMAN
P01619	KV320_HUMAN
P02679	FIBG_HUMAN
P35579	MYH9_HUMAN
P53634	CATC_HUMAN
Q13838	DX39B_HUMAN
P06737	PYGL_HUMAN
P59665	DEF1_HUMAN
P30838	AL3A1_HUMAN
O00391	QSOX1_HUMAN
P80188	NGAL_HUMAN
P61916	NPC2_HUMAN
Q14508	WFDC2_HUMAN
P62805	H4_HUMAN
P17174	AATC_HUMAN
Q9UIV8	SPB13_HUMAN
P07858	CATB_HUMAN
P03973	SLPI_HUMAN
P00558	PGK1_HUMAN
P07384	CAN1_HUMAN
Q16610	ECM1_HUMAN
P0DP23	CALM1_HUMAN
Q13835	PKP1_HUMAN
P02749	APOH_HUMAN
P16870	CBPE_HUMAN
P22392	NDKB_HUMAN
Q06323	PSME1_HUMAN
P08246	ELNE_HUMAN
Q06830	PRDX1_HUMAN
Q96FQ6	S10AG_HUMAN
P12429	ANXA3_HUMAN
Q5D862	FILA2_HUMAN
Q9ULZ3	ASC_HUMAN
P01782	HV309_HUMAN
P00441	SODC_HUMAN
Q96RM1	SPR2F_HUMAN
P05089	ARGI1_HUMAN
P68871	HBB_HUMAN
Q13510	ASAH1_HUMAN
P29320	EPHA3_HUMAN
P43490	NAMPT_HUMAN
Q07955	SRSF1_HUMAN
Q9P1F3	ABRAL_HUMAN

P31944	CASPE_HUMAN
P48163	MAOX_HUMAN
P31146	COR1A_HUMAN
P07195	LDHB_HUMAN
P0CG47	UBB_HUMAN
P01764	HV323_HUMAN
P68431	H31_HUMAN
P30044	PRDX5_HUMAN
Q9Y376	CAB39_HUMAN
Q16696	CP2AD_HUMAN
P52566	GDIR2_HUMAN
Q08554	DSC1_HUMAN
Q9UKR3	KLK13_HUMAN
O75223	GGCT_HUMAN
P21926	CD9_HUMAN
P49913	CAMP_HUMAN
P47756	CAPZB_HUMAN
P07737	PROF1_HUMAN
P01772	HV333_HUMAN
P01019	ANGT_HUMAN
P19961	AMY2B_HUMAN
P55000	SLUR1_HUMAN
P23528	COF1_HUMAN
P63261	ACTG_HUMAN
P17900	SAP3_HUMAN
P06865	HEXA_HUMAN
P80748	LV321_HUMAN
Q16769	QPCT_HUMAN
A0A0C4DH38	HV551_HUMAN
P28799	GRN_HUMAN
P17931	LEG3_HUMAN
Q9UBX7	KLK11_HUMAN
O43490	PROM1_HUMAN
Q6ZVX7	FBX50_HUMAN
P01133	EGF_HUMAN
P08571	CD14_HUMAN
O60814	H2B1K_HUMAN
P61088	UBE2N_HUMAN
P52565	GDIR1_HUMAN
Q96G03	PGM2_HUMAN
P19827	ITIH1_HUMAN
P40926	MDHM_HUMAN
P06753	TPM3_HUMAN
P29034	S10A2_HUMAN
O75449	KTNA1_HUMAN
P62942	FKB1A_HUMAN
P13987	CD59_HUMAN
P17655	CAN2_HUMAN
P11413	G6PD_HUMAN
Q99538	LGMN_HUMAN

P15289	ARSA_HUMAN
P48594	SPB4_HUMAN
P00491	PNPH_HUMAN
Q13867	BLMH_HUMAN
P22532	SPR2D_HUMAN
P42785	PCP_HUMAN
Q92841	DDX17_HUMAN
P47929	LEG7_HUMAN
Q9UKR0	KLK12_HUMAN
P01700	LV147_HUMAN
Q9H299	SH3L3_HUMAN
P26641	EF1G_HUMAN
Q96C90	PP14B_HUMAN
Q13423	NNTM_HUMAN
Q9NY33	DPP3_HUMAN
P51649	SSDH_HUMAN
O14950	ML12B_HUMAN
Q15084	PDIA6_HUMAN
P06576	ATPB_HUMAN
P84077	ARF1_HUMAN
P36955	PEDF_HUMAN
P53597	SUCA_HUMAN
Q9UL46	PSME2_HUMAN
P61158	ARP3_HUMAN
P23083	HV102_HUMAN
Q5T750	KPLCE_HUMAN
P01743	HV146_HUMAN
Q9HCY8	S10AE_HUMAN
P69905	HBA_HUMAN
O14773	TPP1_HUMAN
Q96QA5	GSDMA_HUMAN
O75083	WDR1_HUMAN
Q96QR1	SG3A1_HUMAN
O75629	CREG1_HUMAN
O76031	CLPX_HUMAN
P25787	PSA2_HUMAN
Q99436	PSB7_HUMAN
P28070	PSB4_HUMAN
P29373	RABP2_HUMAN
Q86YZ3	HORN_HUMAN
Q15517	CDSN_HUMAN
P07477	TRY1_HUMAN
P04899	GNAI2_HUMAN
P05090	APOD_HUMAN
P00492	HPRT_HUMAN
P30101	PDIA3_HUMAN
Q14574	DSC3_HUMAN
O00204	ST2B1_HUMAN
Q9BXJ4	C1QT3_HUMAN
Q9UI42	CBPA4_HUMAN

Q14697	GANAB_HUMAN
Q15843	NEDD8_HUMAN
P15309	PPAP_HUMAN
P35030	TRY3_HUMAN
P04062	GBA1_HUMAN
P49721	PSB2_HUMAN
P22735	TGM1_HUMAN
O14818	PSA7_HUMAN
P01040	CYTA_HUMAN
P01824	HV439_HUMAN
P04179	SODM_HUMAN
P04433	KV311_HUMAN
P04792	HSPB1_HUMAN
P14735	IDE_HUMAN
P32119	PRDX2_HUMAN
P42357	HUTH_HUMAN
P48637	GSHB_HUMAN
P49720	PSB3_HUMAN
P60903	S10AA_HUMAN
P61970	NTF2_HUMAN
Q14956	GPNMB_HUMAN
Q6NUJ1	SAPL1_HUMAN
Q8WUM4	PDC6I_HUMAN
Q8WVV4	POF1B_HUMAN
Q99536	VAT1_HUMAN
Q9BWS9	CHID1_HUMAN
Q9H4M9	EHD1_HUMAN
Q9NZD2	GLTP_HUMAN
Q9UJ70	NAGK_HUMAN

Table S11. Proteins identified on the OsseoSpeed®-like titanium dental implant surface after chemical decontamination with NaOCl + AA and recontamination with saliva.

UniProt Accession Number	UniProt Entry Name
P0DTE7	AMY1B_HUMAN
P02768	ALBU_HUMAN
P01036	CYTS_HUMAN
Q9HC84	MUC5B_HUMAN
P01876	IGHA1_HUMAN
P12273	PIP_HUMAN
P01037	CYTN_HUMAN
P01833	PIGR_HUMAN
P60709	ACTB_HUMAN
P02788	TRFL_HUMAN
P23280	CAH6_HUMAN
P22079	PERL_HUMAN
P0CG04	IGLC1_HUMAN
P31025	LCN1_HUMAN
P01834	IGKC_HUMAN
P09228	CYTT_HUMAN
Q96DR5	BPIA2_HUMAN
Q6P5S2	LEG1H_HUMAN
P28325	CYTD_HUMAN
P61626	LYSC_HUMAN
P02787	TRFE_HUMAN
P31151	S10A7_HUMAN
Q8N4F0	BPIB2_HUMAN
P40925	MDHC_HUMAN
P25311	ZA2G_HUMAN
P06703	S10A6_HUMAN
O75556	SG2A1_HUMAN
P10599	THIO_HUMAN
P06702	S10A9_HUMAN
Q8TDL5	BPIB1_HUMAN
P01877	IGHA2_HUMAN
P01024	CO3_HUMAN
Q9UGM3	DMBT1_HUMAN
P06733	ENOA_HUMAN
P01857	IGHG1_HUMAN
A8K2U0	A2ML1_HUMAN
P01591	IGJ_HUMAN
Q9GZZ8	LACRT_HUMAN
P02647	APOA1_HUMAN
P20061	TCO1_HUMAN
Q96DA0	PAUF_HUMAN
P05109	S10A8_HUMAN
P15924	DESP_HUMAN
P01023	A2MG_HUMAN
P63104	1433Z_HUMAN
P01034	CYTC_HUMAN
O43707	ACTN4_HUMAN
P68032	ACTC_HUMAN
Q16378	PROL4_HUMAN

P31947	1433S_HUMAN
Q9Y6R7	FCGBP_HUMAN
P0CF74	IGLC6_HUMAN
P80303	NUCB2_HUMAN
P52209	6PGD_HUMAN
P01871	IGHM_HUMAN
P09211	GSTP1_HUMAN
P01859	IGHG2_HUMAN
P13796	PLSL_HUMAN
P61769	B2MG_HUMAN
P06396	GELS_HUMAN
P00338	LDHA_HUMAN
P55058	PLTP_HUMAN
P06744	G6PI_HUMAN
P60174	TPIS_HUMAN
O95274	LYPD3_HUMAN
P29401	TKT_HUMAN
P29508	SPB3_HUMAN
P07237	PDIA1_HUMAN
P62937	PPIA_HUMAN
Q02487	DSC2_HUMAN
P00738	HPT_HUMAN
Q08188	TGM3_HUMAN
P01861	IGHG4_HUMAN
P00450	CERU_HUMAN
P04080	CYTB_HUMAN
P31949	S10AB_HUMAN
P04066	FUCO_HUMAN
Q96HE7	ERO1A_HUMAN
P02790	HEMO_HUMAN
O00584	RNT2_HUMAN
P09960	LKHA4_HUMAN
Q9UBG3	CRNN_HUMAN
P27482	CALL3_HUMAN
Q6S8J3	POTEE_HUMAN
P07602	SAP_HUMAN
P02774	VTDB_HUMAN
Q01518	CAP1_HUMAN
P30740	ILEU_HUMAN
P04075	ALDOA_HUMAN
Q969S9	RRF2M_HUMAN
P07339	CATD_HUMAN
P04406	G3P_HUMAN
P11021	BIP_HUMAN
P68104	EF1A1_HUMAN
A0A075B6H7	KV37_HUMAN
P25815	S100P_HUMAN
Q08380	LG3BP_HUMAN
Q01469	FABP5_HUMAN
P19021	AMD_HUMAN

P30041	PRDX6_HUMAN
Q02413	DSG1_HUMAN
O60437	PEPL_HUMAN
P54108	CRIS3_HUMAN
P37802	TAGL2_HUMAN
P05164	PERM_HUMAN
P0DMV8	HS71A_HUMAN
O95968	SG1D1_HUMAN
P07108	ACBP_HUMAN
P50395	GDIB_HUMAN
P13639	EF2_HUMAN
P01033	TIMP1_HUMAN
P32926	DSG3_HUMAN
P27797	CALR_HUMAN
P02675	FIBB_HUMAN
P04083	ANXA1_HUMAN
P11142	HSP7C_HUMAN
P04217	A1BG_HUMAN
P62258	1433E_HUMAN
P15311	EZRI_HUMAN
P0C0L4	CO4A_HUMAN
P01860	IGHG3_HUMAN
Q15782	CH3L2_HUMAN
P36952	SPB5_HUMAN
P14923	PLAK_HUMAN
P02763	A1AG1_HUMAN
Q14515	SPRL1_HUMAN
P35321	SPR1A_HUMAN
O60218	AK1BA_HUMAN
P33908	MA1A1_HUMAN
P07900	HS90A_HUMAN
Q13576	IQGA2_HUMAN
P14780	MMP9_HUMAN
P06870	KLK1_HUMAN
Q9UBC9	SPRR3_HUMAN
P37837	TALDO_HUMAN
P12814	ACTN1_HUMAN
P04040	CATA_HUMAN
Q07654	TFF3_HUMAN
P14618	KPYM_HUMAN
P31946	1433B_HUMAN
P24158	PRTN3_HUMAN
P18669	PGAM1_HUMAN
Q9NZT1	CALL5_HUMAN
P05067	A4_HUMAN
P30086	PEBP1_HUMAN
Q96P63	SPB12_HUMAN
Q9NP55	BPIA1_HUMAN
Q9NQ38	ISK5_HUMAN
P80511	S10AC_HUMAN

P18510	IL1RA_HUMAN
P02765	FETUA_HUMAN
P21128	ENDOU_HUMAN
P81605	DCD_HUMAN
P07355	ANXA2_HUMAN
P17213	BPI_HUMAN
P25705	ATPA_HUMAN
P02750	A2GL_HUMAN
P35326	SPR2A_HUMAN
P13797	PLST_HUMAN
P01619	KV320_HUMAN
P02679	FIBG_HUMAN
P35579	MYH9_HUMAN
Q13421	MSLN_HUMAN
P53634	CATC_HUMAN
P06737	PYGL_HUMAN
P59665	DEF1_HUMAN
P30838	AL3A1_HUMAN
O00391	QSOX1_HUMAN
P80188	NGAL_HUMAN
P61916	NPC2_HUMAN
Q14508	WFDC2_HUMAN
P62805	H4_HUMAN
P17174	AATC_HUMAN
Q9UIV8	SPB13_HUMAN
P07858	CATB_HUMAN
P03973	SLPI_HUMAN
P00558	PGK1_HUMAN
P07384	CAN1_HUMAN
O00462	MANBA_HUMAN
Q16610	ECM1_HUMAN
P0DP23	CALM1_HUMAN
Q13835	PKP1_HUMAN
P18206	VINC_HUMAN
P02749	APOH_HUMAN
Q14914	PTGR1_HUMAN
P16870	CBPE_HUMAN
P22392	NDKB_HUMAN
Q07812	BAX_HUMAN
Q06323	PSME1_HUMAN
P08246	ELNE_HUMAN
Q06830	PRDX1_HUMAN
Q96FQ6	S10AG_HUMAN
Q9Y5Z4	HEBP2_HUMAN
P12429	ANXA3_HUMAN
P40121	CAPG_HUMAN
Q5D862	FILA2_HUMAN
O00764	PDXK_HUMAN
Q05639	EF1A2_HUMAN
Q9ULZ3	ASC_HUMAN

Q9UKG9	OCTC_HUMAN
P01782	HV309_HUMAN
P00441	SODC_HUMAN
P01008	ANT3_HUMAN
Q96RM1	SPR2F_HUMAN
P05089	ARGI1_HUMAN
P68871	HBB_HUMAN
Q13510	ASAH1_HUMAN
P29320	EPHA3_HUMAN
P43490	NAMPT_HUMAN
O00748	EST2_HUMAN
P07711	CATL1_HUMAN
Q07955	SRSF1_HUMAN
Q9P1F3	ABRAL_HUMAN
P10909	CLUS_HUMAN
P31944	CASPE_HUMAN
P48163	MAOX_HUMAN
P31146	COR1A_HUMAN
P07195	LDHB_HUMAN
P0CG47	UBB_HUMAN
P01764	HV323_HUMAN
P00751	CFAB_HUMAN
Q14624	ITIH4_HUMAN
Q96BQ1	FAM3D_HUMAN
P30044	PRDX5_HUMAN
Q9Y376	CAB39_HUMAN
Q16696	CP2AD_HUMAN
P52566	GDIR2_HUMAN
P31150	GDIA_HUMAN
Q08554	DSC1_HUMAN
Q9UKR3	KLK13_HUMAN
P62328	TYB4_HUMAN
O75223	GGCT_HUMAN
P21926	CD9_HUMAN
P49913	CAMP_HUMAN
P47756	CAPZB_HUMAN
P07737	PROF1_HUMAN
P01772	HV333_HUMAN
Q02818	NUCB1_HUMAN
Q15435	PP1R7_HUMAN
P19961	AMY2B_HUMAN
O75368	SH3L1_HUMAN
P55000	SLUR1_HUMAN
P23141	EST1_HUMAN
P15328	FOLR1_HUMAN
P62136	PP1A_HUMAN
Q9UHL4	DPP2_HUMAN
P63261	ACTG_HUMAN
Q9Y4L1	HYOU1_HUMAN
P17900	SAP3_HUMAN

P06865	HEXA_HUMAN
P80748	LV321_HUMAN
A0A0C4DH38	HV551_HUMAN
P28799	GRN_HUMAN
P17931	LEG3_HUMAN
Q9UBX7	KLK11_HUMAN
O43490	PROM1_HUMAN
Q6ZVX7	FBX50_HUMAN
P01133	EGF_HUMAN
P08571	CD14_HUMAN
P61088	UBE2N_HUMAN
P52565	GDIR1_HUMAN
Q96G03	PGM2_HUMAN
P19827	ITIH1_HUMAN
Q9BPY8	HOP_HUMAN
Q15080	NCF4_HUMAN
P40926	MDHM_HUMAN
P22894	MMP8_HUMAN
P02766	TTHY_HUMAN
P06753	TPM3_HUMAN
P29034	S10A2_HUMAN
O75449	KTNA1_HUMAN
P99999	CYC_HUMAN
P54802	ANAG_HUMAN
P62942	FKB1A_HUMAN
Q12841	FSTL1_HUMAN
P26038	MOES_HUMAN
P13987	CD59_HUMAN
P19823	ITIH2_HUMAN
P17655	CAN2_HUMAN
Q8NFT8	DNER_HUMAN
A0A0C4DH36	HV338_HUMAN
P11413	G6PD_HUMAN
Q99538	LGMN_HUMAN
P06312	KV401_HUMAN
P15289	ARSA_HUMAN
Q9BX40	LS14B_HUMAN
P48594	SPB4_HUMAN
Q6XQN6	PNCB_HUMAN
P04004	VTNC_HUMAN
Q9UBH0	I36RA_HUMAN
P09668	CATH_HUMAN
P00491	PNPH_HUMAN
O60235	TM11D_HUMAN
Q13867	BLMH_HUMAN
Q8N474	SFRP1_HUMAN
P22532	SPR2D_HUMAN
P42785	PCP_HUMAN
Q92841	DDX17_HUMAN
P12830	CADH1_HUMAN

P47929	LEG7_HUMAN
O43852	CALU_HUMAN
Q9Y697	NFS1_HUMAN
Q6UX06	OLFM4_HUMAN
Q9UKR0	KLK12_HUMAN
P01700	LV147_HUMAN
Q9H299	SH3L3_HUMAN
P26641	EF1G_HUMAN
Q13423	NNTM_HUMAN
P23284	PPIB_HUMAN
Q9NY33	DPP3_HUMAN
P55786	PSA_HUMAN
P08311	CATG_HUMAN
P51649	SSDH_HUMAN
Q9UBR2	CATZ_HUMAN
P14314	GLU2B_HUMAN
P09958	FURIN_HUMAN
O14950	ML12B_HUMAN
Q15084	PDIA6_HUMAN
Q05707	COEA1_HUMAN
P14174	MIF_HUMAN
P06576	ATPB_HUMAN
O60293	ZC3H1_HUMAN
P01780	HV307_HUMAN
P84077	ARF1_HUMAN
O60888	CUTA_HUMAN
P36955	PEDF_HUMAN
P30046	DOPD_HUMAN
Q8WWA0	ITLN1_HUMAN
A0A075B6R9	KVD24_HUMAN
O75071	EFC14_HUMAN
P53597	SUCA_HUMAN
Q9UL46	PSME2_HUMAN
P61158	ARP3_HUMAN
A0A0C4DH35	HV335_HUMAN
Q5SSG8	MUC21_HUMAN
P23083	HV102_HUMAN
O75976	CBPD_HUMAN
P48723	HSP13_HUMAN
Q5T750	KPLCE_HUMAN
P60981	DEST_HUMAN
P01817	HV205_HUMAN
Q92876	KLK6_HUMAN
P01743	HV146_HUMAN
Q9HCY8	S10AE_HUMAN
P69905	HBA_HUMAN
O14773	TPP1_HUMAN
O75015	FCG3B_HUMAN
P22626	ROA2_HUMAN
P02671	FIBA_HUMAN

O75083	WDR1_HUMAN
Q96QR1	SG3A1_HUMAN
O75629	CREG1_HUMAN
O76031	CLPX_HUMAN
P25929	NPY1R_HUMAN
P12109	CO6A1_HUMAN
P25787	PSA2_HUMAN
P28070	PSB4_HUMAN
P60660	MYL6_HUMAN
A6NIZ1	RP1BL_HUMAN
A0A0B4J1V0	HV315_HUMAN
P29373	RABP2_HUMAN
Q9Y287	ITM2B_HUMAN
Q86YZ3	HORN_HUMAN
P15153	RAC2_HUMAN
P40189	IL6RB_HUMAN
P01880	IGHD_HUMAN
Q9GZM7	TINAL_HUMAN
P07477	TRY1_HUMAN
P04899	GNAI2_HUMAN
P05090	APOD_HUMAN
P00492	HPRT_HUMAN
P08670	VIME_HUMAN
P01721	LV657_HUMAN
Q10588	BST1_HUMAN
O75594	PGRP1_HUMAN
P68036	UB2L3_HUMAN
A0A0A0MS15	HV349_HUMAN
O00204	ST2B1_HUMAN
Q14118	DAG1_HUMAN
P35052	GPC1_HUMAN
Q8NBJ4	GOLM1_HUMAN
Q9UI42	CBPA4_HUMAN
P26885	FKBP2_HUMAN
Q14697	GANAB_HUMAN
Q9BQR3	PRS27_HUMAN
P19652	A1AG2_HUMAN
Q96S96	PEBP4_HUMAN
P01599	KV117_HUMAN
A0A0B4J1Y9	HV372_HUMAN
Q15843	NEDD8_HUMAN
O95479	G6PE_HUMAN
P15309	PPAP_HUMAN
P35030	TRY3_HUMAN
P59998	ARPC4_HUMAN
P04062	GBA1_HUMAN
O75874	IDHC_HUMAN
P49721	PSB2_HUMAN
P22735	TGM1_HUMAN
E9PAV3	NACAM_HUMAN

O14522	PTPRT_HUMAN
O43592	XPOT_HUMAN
O60522	TDRD6_HUMAN
P01824	HV439_HUMAN
P04433	KV311_HUMAN
P04792	HSPB1_HUMAN
P05156	CFAI_HUMAN
P08174	DAF_HUMAN
P08582	TRFM_HUMAN
P15531	NDKA_HUMAN
P36871	PGM1_HUMAN
P40394	ADH7_HUMAN
P42357	HUTH_HUMAN
P48637	GSHB_HUMAN
P49720	PSB3_HUMAN
P49902	5NTC_HUMAN
P60903	S10AA_HUMAN
P61970	NTF2_HUMAN
Q00610	CLH1_HUMAN
Q04446	GLGB_HUMAN
Q12907	LMAN2_HUMAN
Q14CN2	CLCA4_HUMAN
Q15691	MARE1_HUMAN
Q6NUJ1	SAPL1_HUMAN
Q6UW32	IGFL1_HUMAN
Q8WUM4	PDC6I_HUMAN
Q99536	VAT1_HUMAN
Q9BRA2	TXD17_HUMAN
Q9BWS9	CHID1_HUMAN
Q9NZD2	GLTP_HUMAN
Q9UJ70	NAGK_HUMAN
Q9UJC5	SH3L2_HUMAN
